# Identification of potential biomarkers in donor cows for *in vitro* embryo production by granulosa cell transcriptomics

**DOI:** 10.1371/journal.pone.0175464

**Published:** 2017-04-12

**Authors:** Gianluca Mazzoni, Suraya M. Salleh, Kristine Freude, Hanne S. Pedersen, Lotte Stroebech, Henrik Callesen, Poul Hyttel, Haja N. Kadarmideen

**Affiliations:** 1 Animal Breeding, Quantitative Genetics & Systems Biology (AQS) Group, Department of Veterinary and Animal Sciences, Faculty of Health and Medical Sciences, University of Copenhagen, Frederiksberg C, Denmark; 2 Department of Veterinary and Animal Sciences, Faculty of Health and Medical Sciences, University of Copenhagen, Frederiksberg C, Denmark; 3 Department of Animal Science, Aarhus University, Tjele, Denmark; 4 EmbryoTrans Biotech A/S, Haslev, Denmark; 5 Department of Bio and Health Informatics, Technical University of Denmark, Kongens Lyngby, Denmark; INIA, SPAIN

## Abstract

The Ovum Pick Up-*In vitro* Production (OPU-IVP) of embryos is an advanced reproductive technology used in cattle production but the complex biological mechanisms behind IVP outcomes are not fully understood. In this study we sequenced RNA of granulosa cells collected from Holstein cows at oocyte aspiration prior to IVP, to identify candidate genes and biological mechanisms for favourable IVP-related traits in donor cows where IVP was performed separately for each animal. We identified 56 genes significantly associated with IVP scores (BL rate, kinetic and morphology). Among these, *BEX2*, *HEY2*, *RGN*, *TNFAIP6* and *TXNDC11* were negatively associated while *Mx1* and *STC1* were positively associated with all IVP scores. Functional analysis highlighted a wide range of biological mechanisms including apoptosis, cell development and proliferation and four key upstream regulators (COX2, IL1, PRL, TRIM24) involved in these mechanisms. We found a range of evidence that good IVP outcome is positively correlated with early follicular atresia. Furthermore we showed that high genetic index bulls can be used in breeding without reducing the IVP performances. These findings can contribute to the development of biomarkers from follicular fluid content and to improving Genomic Selection (GS) methods that utilize functional information in cattle breeding, allowing a widespread large scale application of GS-IVP.

## Introduction

The need for increased efficiency of food production calls for more widespread implementation of novel precision breeding strategies. In this context, Genomic Selection (GS), which is based on estimating breeding values using genome-wide markers identified using high-density SNP chips, can have a huge impact, as reviewed in [1, 2]. This technology enables rapid genetic improvement via a significant reduction in generation interval, increased accuracy of estimated breeding values and high intensity of selection. GS has made a substantial economic impact due to reduction in the cost of traditional progeny and performance tests in livestock [1, 2].

The combination of GS with artificial reproductive techniques such as ultrasound-guided ovum pick up (OPU) and subsequent *In vitro* Production (IVP) of embryos can further accelerate and increase genetic improvements. The combined use of GS, OPU and IVP (GS-OPU-IVP) offers several advantages: selected oocyte donor animals can produce many calves, GS on embryo biopsies can increase efficiency in breeding and shorten the generation interval significantly; an effect that can be further substantiated by the effect of harvesting oocytes from even prepubertal heifers.

Unfortunately, despite huge effort, the IVP procedures are not fully optimized and their efficiency is still relatively low. In cattle, maturation, fertilization and culture *in vitro* of cumulus-oocyte complexes (COCs) of good morphology result in only 35–45% developing to the blastocyst (BL) stage [3, 4]. These drawbacks have a major impact on the implementation of the technology.

The outcome of IVP measured in terms of embryo quality and pregnancy rate has been attributed mainly to the oocyte [3, 5, 6], but other studies showed that the sperm also plays a role from the first days of embryo development [7–9]. Effects of the sperm on the timing of first cleavage, the BL morphology and the pregnancy rate have been noted [7]. In the context of IVP combined with GS, the possibility of using sperm from bulls of high genetic merit without compromising the IVP outcome is of fundamental importance, but has not been examined.

The poor IVP efficiency has been mainly attributed to the lower competence of the oocytes, which are aspirated from growing antral follicles and forces to mature *in vitro* over a 24 hour period as compared with the superior *in vivo* development in the dominant follicle culminating in oocytes maturation and ovulation [10, 11]. Developmental competence is defined as the ability of the oocytes to develop into BLs that are suitable for transfer [3, 11, 12]. Huge efforts have been invested in the identification of biomarkers of oocyte competence in different domestic species as well as in humans. Oocyte competence is probably related to the synthesis and storage of transcripts and proteins during oocyte growth (for a review, see [13]). These molecules are of fundamental importance, because they support development through oocyte maturation and fertilization to the activation of the embryonic genome [5, 6], which occurs at the 8-cell stage in cattle [14].

Transcriptomics can help in identifying biomarkers of oocyte and embryo competence [15]. In cattle, many studies have exploited the power of Next-Generation Sequencing (NGS) technologies to identify biomarkers in the follicular compartments and small tissue biopsies [16]. Cumulus and granulosa cells are intimately coupled to the oocyte through paracrine and intercellular communication systems and play major roles in oocyte competence [17]. Moreover, these cellular compartments reflect the characteristics of the oocytes and represent assessable targets for analyses, as they are aspirated together with the COCs. The cumulus cells attached to the oocyte play a fundamental role during *in vitro* oocyte maturation and also have effects during *in vitro* fertilization [18, 19]. For these reasons, removal of cumulus cells from the COCs before *in vitro* fertilization can negatively affect the IVP outcome. On the other hand, collection of granulosa cells and cumulus cells found in the follicular fluid is a less invasive method, as these cells are a by-product of COC aspiration. Many studies have focused on identification of biomarkers of oocyte competence in the follicular fluid. A complex picture of a variety of molecules from different families and their biochemical pathways associated with oocyte competence is now emerging [20]. Midkines showed a positive effect exerted through granulosa and cumulus cells on bovine oocyte competence during *in vitro* maturation [21]. A similar effect on the oocyte competence exerted through granulosa cells has been identified for proline when added to the media during maturation [22]. The expression levels for five genes (*FDX1*, *CYP19A1*, *CDC42*, *SERPINE2*, *3bHSD1*) have been positively associated with oocyte competence in analysing human granulosa cells from aspirated fluid [23]. In a study based on rat granulosa cells, the expression level of 13 genes correlated with oocyte developmental competence. Among these, 12 genes were overexpressed in normal developmental competence group versus poor developmental competence group. The genes were primarily involved in: copper ion binding (*Lox*), apoptosis induction (*Ngfrap1*) while the underexpressed gene was involved in regulation of extracellular matrix (ECM) organization (*Ggbt2*)[24]. The expression of 40 transcripts (18 overexpressed and 22 underexpressed) has been associated with developmental competence in oocytes from follicles aspirated before the luteinizing hormone (LH) surge that precedes the ovulation phase. The pre-LH surge phase was defined as the best period for studying biomarkers of oocyte competence in granulosa cells [25]. Follicle-stimulating hormone (FSH) withdrawal after super stimulation, defined as FSH coasting, was analysed to see the effect on the gene expression in granulosa cells. FSH coasting is particularly interesting, because it has a positive effect on oocyte competence in bovines [26]. A positive association with the FSH coasting has been confirmed with real-time PCR for 11 genes (*KCNJ8*, *NRP1*, *IGF2*, *GFPT2*, *TF*, *RELN*, *ANKRD1*, *ANXA1*, *BMPR1B*, *TFPI2*, *VNN1*) [27]. An increase in the expression of gene for the luteinizing hormone receptor (*LHCGR*) has also been associated with oocyte competence [28]. Many studies have focused on understanding how the granulosa cell profile varies between follicles with different characteristics, for example follicles at different developmental stages [29] and follicles of different sizes [30], and between healthy and atretic follicles [31].

None of the previous studies focus on the single animal level. Usually the oocytes and the RNA samples are pooled together within the experimental condition; otherwise, analyses are conducted performing case and control studies to test the effect of the substances or hormones of interest on gene expression.

Earlier studies have, through repeated OPU sessions, demonstrated that certain donor cows are superior for OPU-IVP [32]. Thus, the possibility of identifying biomarkers in follicular cells at the single animal level is of extreme interest in order to improve GS-OPU-IVP and enable more widespread application. This would enable selection of the best oocyte donor cows based on biomarker levels. Furthermore, eQTL studies can identify genetic variants controlling for the expression of these biomarkers and this new information can be included in GS exploiting the medium to high heritability of IVP-related traits in cows [33].

The main objectives of this study were i) to investigate the possible effect of using bulls with high versus low breeding values (Nordic Total Merit (NTM)) on IVP outcome, and ii) to investigate the effect of the individual cow granulosa cell transcriptomic profiles on IVP outcome. In order to achieve these objectives, we performed RNA sequencing of granulosa cells isolated from follicular fluid to identify genes correlated with IVP outcome measured as BL rate, morphology and kinetics.

To the best of our knowledge, this is the first study to analyse the effect of the genetic merit of bulls on IVP outcome and to focus on transcriptomic profiles of pools of follicular cells at the single animal level. The results have provided proof that high NTM bulls do not have inferior IVP performance and revealed which candidate genes might be used for further biomarkers discoveries for donor cow selection in this species.

## Materials and methods

### Sample collection and IVP procedure

Ovaries from 67 Danish cows were collected immediately after slaughter from a local abattoir (Mogens Nielsen Kreaturslagteri A/S; 55°18’, 11°44’). The sampling was performed in six rounds collecting 10 to 12 cows per round.

Each pair of ovaries was kept separate from the others and labelled with the cow’s Central Husbandry Register (CHR) number, which is the national system for all registered cows in Denmark. Immature COCs from each animal were retrieved from antral follicles using a vacuum pump. The COCs from each animal were collected and kept separately.

Denuded oocytes were discarded. The aspirated fluids containing only follicular cells were collected in 15 ml TPP centrifuge tubes, one for each animal, and they immediately underwent purification and freezing procedures. (see S1 Text).

The COCs were *in vitro* matured (IVM), inseminated (IVF) and cultured (IVC) until the BL stage (day eight). Insemination was performed according to the experimental design: each bull was used for insemination of oocytes from three cows, and for each experimental round, oocytes from half of the cows were inseminated with bulls with high NTM [34] and half with low-NTM bulls.

NTM is the Nordic Total genetic Merit index this is calculated by the NAV (Nordic Cattle Genetic Evaluations) taking in consideration animal trait groups for production, functionality and conformation weighted based on economic calculations [34].

The COC’s and blastocysts were made with media from IVF Bioscience, Falmouth, United Kingdom, and the procedure according to their IVP protocol. A detailed description of the IVP protocol can be found in S1 Text.

### Measurement of IVP scores

On day eight, embryos from all the animals were scored with regard to three parameters:

BL rate was computed for each animal as the number of BLs over the total number of inseminated COCs. Kinetic score was obtained by visual classification of each BL as non-expanded, expanded or hatching/hatched. The three classes were scored respectively 1, 2 or 3, and finally, for each animal we computed the average score. Morphology score was obtained by visual classification of each BL as poor, good or excellent. Poor morphology is defined as the presence of diffuse or absence of inner cell mass (ICM), degenerated trophoblast cells or much fragmentation, or irregular or uneven trophoblast morphology. Good morphology was assigned where there was presence of smaller or less distinct ICM, few degenerated trophoblast cells, slight fragmentation, or slightly irregular or slightly uneven trophoblast morphology. Excellent morphology is defined by the presence of compact, large and distinct ICM, or regular and even trophoblast morphology. To each class respectively we assigned the scores 1, 2 or 3 and computed the average for each animal.

The animals were not synchronized before collecting COCs, therefore the number of COCs can vary in relation to these effects. However, all the IVP outcomes or scores (such as BL rate, morphology and kinetic) used in the analysis are independent of the total number of COCs for each cow.

### RNA extraction

RNA was isolated from the frozen samples using an RNeasy Plus Micro Kit (Qiagen, Hilden, Germany), according to the manufacturer’s instructions. RNA samples were immediately frozen and stored at -80°C. All the RNA samples were tested and quantified with a NanoDrop ND-1000 Spectrophotometer (Saveen Werner) and with an Agilent 2100 Bioanalyzer.

### Sample selection

Information about the animals—breed, age at slaughter (days), calving number and herd of production—was retrieved from the SEGES Dairy & Beef Research Centre database using the CHR identification number. Only Holstein cows were used in the rest of the analysis.

We ended up with a total of 34 Holstein samples, from 14 different herds, with an average age at slaughter of 1,594 days (SD = 401.36, min = 928, max = 2,930). Their oocytes were inseminated with semen from 16 bulls, seven with low and nine with high NTM index. Oocyte pools of twenty cows were fertilized with high-NTM bulls and oocyte pools of 14 cows were fertilized with low-NTM bulls. Only RNA samples from first- or multiple-lactation Holstein cows were used and samples with an RNA Integrity Number (RIN) lower than 5.8 were excluded in order to select the top 24 samples. The RNA samples selected had an average RIN value of 7.21 (min = 5.8 and max = 8.6), all derived from granulosa cells (mural granulosa and granulosa from the cumulus) collected from multiparous Holstein cows (from 1 to 6 calvings) all at the luteal phase of the oestrous cycle (day 3 to day 17). The average age at slaughter was 1638 days (SD = 417, min = 928, max = 2,930). Data represented 10 herds and six different experimental dates.

### RNA sequencing

The selected samples were paired-end sequenced with the Illumina HiSeq 2500 platform with a read length of 100 nt, obtaining an average of 84,481,280 reads per sample (min = 71,735,902, max = 98,870,402). The libraries for the sequencing were generated with an Illumina TruSeq-stranded totalRNA RiboZero Gold Sample Prep Kit.

All RNA-Seq data have been deposited in NCBI’s Gene Expression Omnibus and are accessible through GEO accession number GSE94541. (https://www.ncbi.nlm.nih.gov/geo/query/acc.cgi?acc=GSE94541).

### Real-time PCR

Validation of RNA-Seq data was performed by real-time PCR on 4 of the top 7 candidate genes (*BEX2*, *Mx1*, *STC1* and *TXNDC11*) and using *GAPDH* as a reference gene.

The genes were tested on 21 samples out of the original 23 samples, which have been subjected to RNA sequencing. Two samples could not be included because all the RNA collected was used for the RNA sequencing experiment. The primers for *BEX2*, *Mx1*, *STC1*, *TXNDC11* and *GAPDH* were designed using the version UMD3.1 of *Bos taurus* assembly and the Primer3 program as a primer design tool. Primer sequences and transcript accession numbers are provided in **Table A in** S1 File.

Briefly, for each sample 200 ng of total RNA was reverse transcribed to cDNA using RevertAid First Strand cDNA Synthesis Kit (Thermo Scientific) following manufacturer's recommendations and using both oligo-dT and random primers. Real-time PCR was performed using LightCycler 480 and SYBR Green I Master (Roche) following manufacturer’s instruction. The PCR conditions used for all genes were as follows: 5 min preincubation at 95°C followed by 45 PCR cycles (denaturation 95°C for 10 s; annealing 60° for 10 s; extension 72°C for 20 s), a melting curve (95°C for 5 s, 65°C for 1 min, from 65°C up to 97°C at 0.11°C/s), and a final cooling step at 40°C.

Real-time PCR reactions for the same genes were run all in one plate. We checked for across plate variation including a calibrator that consisted of a pool of equal concentrations of cDNA from all the samples. Each sample including the calibrator was run in triplicate.

#### Real-time PCR data analysis

The average crossing point-PCR-cycle (Cp) for each reaction was computed with LightCycler^®^ 480 Software version 1.5.1) using the second-derivative maximum method.

No relevant variation was observed across plates. In order to validate the RNA-Seq data we computed the Pearson correlation between target gene and GAPDH ratios from RNA-Seq raw counts vs. the qPCR average Cp.

### Bioinformatic analysis

#### RNA-Seq preprocessing, alignment and gene expression quantification

All the steps from the raw reads to the gene expression quantification were performed on our group’s high-performance computing platforms (Operative system: openSUSE 13.1 Bottle (x86_64), Linux version: 3.11.10-7-desktop, RAM: 504 GB, #CPUs: 64).

The quality of the reads was checked with *FastQC* (v. 0.11.2) [35] for both forward and reverse reads before proceeding with the pre-mapping quality control of the reads. Adapters were removed using *cutadapt* (v. 1.6) [36].

Raw reads were filtered and trimmed by quality using *PRINSEQ* (v. 0.20.4) [37] to compute the: (1) trimming of the 3'-end with a quality threshold of 20, (2) filtering of the sequence with an average quality lower than 20, (3) filtering reads with a final length of less than 25 nucleotides. The remaining reads were mapped to the bovine reference genome (*Bos taurus* UMD3.1) [38] with *STAR aligner* (v. 2.3.0) [39], using the two-pass method and including the gene annotation file. We allowed for a maximum of five mismatches, while setting the other parameters to *STAR* default values. Splice junction files obtained from the first step were filtered. We excluded previously annotated junctions, junctions identified in the mitochondrial genome and not significant junctions [39].

Read counts were estimated at gene level using *HTSeq-count* (v. 0.6.0) [40], setting the model of intersection as “intersection-nonempty”, library type as “reverse” and using the same annotation file (Ensemble *Bos taurus* 10.2.83).Further quality controls on count data were performed with *Noiseq* (v.2.14.0) [41] in order to identify the presence of transcript length bias or GC content biases.

PCA and hierarchical clustering were then applied on normalized data, to identify if the main variation could be explained by one or more potential confounders and to check for outliers.

#### Filtering low-count genes and normalization

We filtered all the genes with less than 1 count per million (cpm) in more than 90% of the samples using the function *cpm* from *edgeR* v. 3.10.5. Length and GC biases identified with NOISeq were reduced using the conditional quantile normalization methods *cqn* function from cqn (R package v.1.14.0) [42].

The library size factors were computed with function *size factors* of DESeq2 v.1.8.2 [43]. The length of each gene was computed as the median of the lengths of its transcript. Lengths and GC percentages were retrieved from Ensemble *Bos taurus* 10.2.83 using Biomart [44]

#### Analysis of the sperm effect

A simple linear model was fitted to test the effect of the genetic merit index of the bulls’ sperm on the IVP outcome. The basic linear model is:

$$y_{i}=\beta_{0}+\beta_{age}{\text{age}}_{i}+\sum_{z=1}^{Z-1}\beta_{date,z}date_{i,z}+\beta_{NTM}NTM_{i}+\xi_{i}$$ (1)

where y_i_ is the IVP score (BL rate or morphology or kinetic scores) for the *i*^th^ animal, β_0_ is a fixed intercept term, NTM was fitted as a dummy variable distinguishing between two classes of high and low NTM index, and β_NTM_ is the corresponding regression coefficient.

Age at slaughter (age) was included as an explanatory variable and fitted as a covariate, whereas sampling date (date) was fitted as a fixed effect with classes in the model where date _i(z,…Z-1)_ is a set of (Z-1) dummy variables representing the sampling date for the *i*^th^ animal, and β_date,z_ and β_age_ are the solutions for date classes and regression coefficients, respectively.

In total, we fitted three univariate linear models, one for each IVP outcome variable that we tested (BL rate, morphology and kinetic) on the complete data set (34 Holstein cows). All the models were fitted using the *lm* function (R package v. 3.2.2).

#### Residuals computation

In order to avoid biases due to sperm quality effect on the IVP, we adjusted the phenotype data (IVP outcome) for the sperm effect and used the “adjusted phenotype” for our differential gene expression analyses.

The effect of the bulls’ sperm on the IVP phenotype was first estimated by using the simple linear model:

$$y_{i}=\beta_{0}+\sum_{k=1}^{K-1}\beta_{bull,k}bull_{ik}+\xi_{i}$$ (2)

where y_i_ is the IVP score for the *i*^th^ animal, β_0_ is a fixed intercept term, bull_(k…K-1)_ is a set of (K-1) dummy variables distinguishing between the bulls used during the IVF step, and β_bull,k_ is the corresponding regression coefficient.

The adjusted phenotype was then defined as residuals that correspond to the IVP outcome after correcting for estimated mean and sperm effects from (Eq 2):

$${\hat{e}}_{i}=y_{i}-\left({\hat{\beta }}_{0}+\sum_{k=1}^{K-1}{\hat{\beta }}_{bull,k}bull_{i,k}\right)$$ (3)

where ${\hat{\beta }}_{0}$ and ${\hat{\beta }}_{\text{bull,}\left(\text{k, }\ldots \text{, K}-1\right)}$ are the least squares estimates obtained using the *lm* function from *stats* (R package v. 3.2.2) and e_ij_ are the residuals computed with the function *residuals* (R package v. 3.2.2).

The adjusted phenotype (the residuals in (Eq 3)) represents part of the IVP scores that cannot be explained by the effect of sperm.

Gene expression analysis for all the IVP scores was performed using the adjusted phenotype computed in (Eq 3) instead of the raw IVP scores.

#### Gene expression analysis

The genes whose expressions were associated with the IVP outcome were identified with DESeq2 v. 3.2.2. DESeq2 performs a logistic regression of the gene counts (modelled by a negative binomial distribution) with the IVP scores. The statistical significance of the regression model was evaluated by Wald test statistics [43].

We included in the model the following: age at slaughter and the RIN values as continuous variables and the sampling date as a set of dummy variables in order to adjust the expression data for these explanatory variables. The RIN values observed are probably due mainly to technical issues (processing time of the samples) occurred during the samples collection.

Considering that our main objective is to find candidate genes for IVP traits, the RIN was included in the model to account for these biases. During the independent filtering step, 545 and 3267 other genes were filtered out for the analysis of the BL rate and morphology, respectively. Significantly associated genes were called at a FDR of 5%.

#### Comparison with gene expression profiles related to follicle size and atresia

We compared our patterns of gene expression to previous analyses that reported direction or FCs of differential gene expression for healthy versus early antral atretic follicles by (Hatzirodos *et al*.(b)) [31] as well as for small (3–5 mm) versus medium and large follicles (> 9 mm) by (Hatzirodos *et al*.(a)) [30].

We considered all the significant genes identified in (Hatzirodos *et al*.(a, b)) [30, 31] for comparison with expression profiling results from our analyses. A gene was considered to have the same trend when it showed the same direction of change. We performed the same comparison selecting only the genes with log2 FCs for BL rate with an absolute value higher than 0.10, resulting in the top 25%. This comparison ensured that the most significant results from our study conformed to those reported in (Hatzirodos *et al*.(a,b)) [30, 31].

#### Enrichment analysis

We performed gene set enrichment analysis using the *GseaPreranked* tool from GSEA v2.2.2 (Broad Institute) [45] based on gene sets from Molecular Signatures Database (MSigDB Collections) v5.1. GSEA was able to map 9831 genes. We considered an FDR threshold of 5% as significant and an FDR of 25% as suggestive, as recommended by the GSEA manual [46].

We performed a second functional analysis using QIAGEN’s Ingenuity^®^ Pathway Analysis (IPA^®^, QIAGEN Redwood City,www.qiagen.com/ingenuity). The analysis was performed using all the genes with a 10% FDR. In total, 8911 genes were mapped (**Tables B, C and D in** S1 File).

## Results

### RNA-Seq statistics and preliminary analysis

From the alignment, we obtained on average of 79.36% of uniquely mapped reads per sample, which corresponds to an average of 33,448,723 uniquely mapped read pairs. The alignment files were analysed with *Qualimap* (v.2.0.2) [47] to count the number of reads mapping in exonic, intronic and intergenic regions. Among the uniquely mapped read pairs, on average 43.80% mapped to exonic, 16.63% to intergenic and 18.93% to intronic regions (**Fig A in** S2 File). Sample 8 showed a very low fraction of uniquely mapped read pairs compared to the other samples (50.78% of uniquely mapped reads, with only 27.73% mapped to exonic regions).

The hierarchical clustering and the Principal Component Analysis (PCA) plots confirmed that Sample 8 was an outlier. Thus, it was excluded from the rest of the analysis. We filtered genes with low counts in the remaining 23 samples; we ended up with an expression count matrix for 10,891 genes (10,617 coding genes, 206 non-coding genes, 68 pseudogenes).

We inspected the PCA plot to determine whether some of the main variation in the data set could be explained by one or more of these potential confounders. The variation captured by the first component correlated with the RNA Integrity Number (RIN) values (**Figs B and C in** S2 File). RIN was included in the model for gene expression analysis as a confounding effect.

### Evaluation of theca cell contamination

We checked for the presence of theca contamination by looking at the expression of the gene *CYP17A1*. CYP17A1 i*s* synthesized in theca cells and only at low level in granulosa cells [48]. By checking the level of expression of *CYP17A1* we can identify the presence of theca cell contamination [31]. In our samples, *CYP17A1* is mildly expressed and it was filtered out during the filtering step. We can conclude that the concentration of theca cells is not sufficient to significantly alter the gene expression profiles. We expect that the RNA samples that we analysed belonged mainly to mural granulosa cells and partially to granulosa cells of the cumulus oophorus.

### IVP outcome

The results of the IVP procedures are summarized in Table 1. The average number of COCs that underwent IVP from each animal was 11.60 and the average BL rate obtained was 37%, which was consistent with results from previous studies. The 23 RNA samples chosen for sequencing showed statistics similar to the complete data set, and this subset of animals is representative of the entire set. All the IVP scores that we computed showed positive pairwise Pearson Correlation Coefficients (PCC). Morphology and kinetic showed the strongest correlation (PCC = 0.79). BL rate showed a better correlation with kinetic (PCC = 0.73) than with morphology (PCC = 0.55).

**Table 1:** Summary of the IVP scores for the sample sets.

	Average	SD	Min	Max
COCs (34 animals)	11.60	7.38	3.00	30.00
COCs (23 animals)	11.13	7.88	3.00	30.00
BL rate (34 animals)	37%	18%	0%	71%
BL rate (23 animals)	37%	19%	0%	71%
Kinetic (34 animals)	1.40	0.42	0.00	1.88
Kinetic (23 animals)	1.31	0.48	0.00	1.88
Morphology (34 animals)	2.17	0.73	0.00	3.00
Morphology (23 animals)	2.11	0.85	0.00	3.00

The table shows the statistics computed for the set of 34 Holstein cows used to estimate the sperm effect and for the 23 Holstein cows included in the RNA analysis for the identification of biomarkers. **SD** = standard deviation, **Min** = minimum value, **Max** = maximum value.

The correlations computed pairwise among the IVP scores for the 23 animals after removing the effect of the bulls (using the residuals) increased for all scores. The correlation between kinetic and BL rate increased (PCC = 0.79). The same occurred for morphology and kinetic (PCC = 0.92), while PCC between morphology and BL rate increased to 0.60.

### Genetic merit index effect

**Fig 1:**
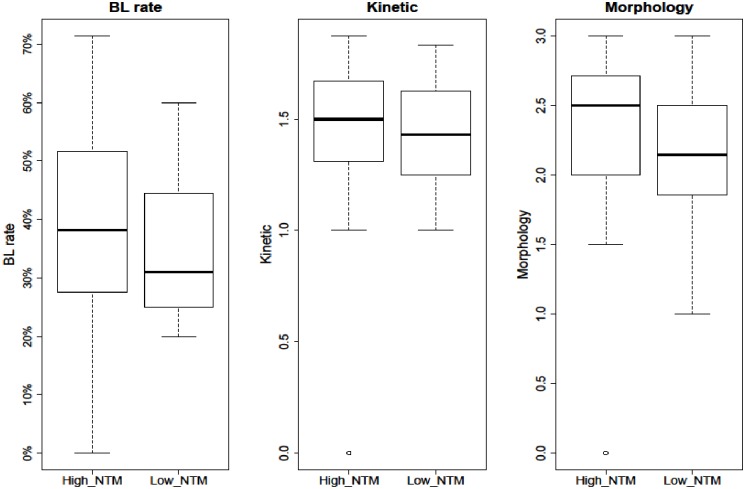
Box plots representing the differences in the distribution of IVP scores between samples inseminated with high-NTM and low-NTM index bulls. The thick line represents the median values. The box represents the lower and upper quartiles. The thin lines are the maximum and minimum values excluding outliers. Outliers are represented by empty circles.

The phenotypic means of BL rate and morphology tended to be lower when sperm from low-NTM bulls was used for IVP; in other words, high-NTM bulls tended to have more favourable IVP outcomes (Fig 1). However, when we fitted the linear models to formally test the effect of the bull genetic index while taking into consideration possible confounding effects, no statistically significant effect could be observed at a P value of 0.05.

For the morphology score (β = -0.31, P value = 0.20) we obtained a 10.33% decrease on the entire scale, followed by a BL rate (β = -0.31, P value = 0.59) that showed a 3.8% decrease using low-NTM index bull sperm. Conversely, we obtained a 3% increase of the kinetic score (β = 0.09, P value = 0.58).

### Genes related to IVP outcome

We identified 45 genes significantly related to the BL rate (False Discovery Rate (FDR) < 5%) of which 10 showed a positive correlation (their expressions were increased in cows with higher BL rates). For the kinetic score we identified 30 genes (11 positively correlated and 19 negatively correlated). Ten genes were significantly related to morphology, including three that were positively correlated (**Table E, F and G in** S1 File). Seven genes resulted in being common among all the IVP scores: *BEX2*, *HEY2*, *Mx1*, *RGN*, *STC1*, *TNFAIP6* and *TXNDC11*, and two of these (*Mx1*, *STC1*) were positively correlated (Table 2). The scatterplots for these 7 genes are shown in **Fig D in** S2 File.

**Table 2:** Summary of the Fold Change (FC) and the corresponding FDR for the genes in common among all the IVP scores.

Gene Name	BL rate		Kinetic		Morphology	
	FC	FDR	FC	FDR	FC	FDR
*BEX2*	-0.18	6.96E-04	-0.25	3.17E-02	-0.18	4.50E-02
*HEY2*	-0.17	1.44E-02	-0.30	1.67E-02	-0.22	4.40E-02
*Mx1*	+0.16	4.65E-02	+0.55	1.90E-04	+0.26	4.40E-02
*RGN*	-0.17	1.74E-03	-0.26	2.18E-02	-0.18	4.50E-02
*STC1*	+0.15	4.65E-02	+0.57	5.63E-05	+0.27	2.47E-02
*TNFAIP6*	-0.15	4.03E-06	-0.20	2.80E-03	-0.12	4.50E-02
*TXNDC11*	-0.16	5.91E-03	-0.29	1.81E-02	-0.20	4.50E-02

**FC** = change in gene expression for a 1 unit change in kinetic and morphology, while for BL rate it represents the amount of change in gene expression for each 20% increase in BL rate. The plus sign (+) indicates the presence of a positive correlation, while minus (–) indicates a negative correlation, **FDR** = Benjamini-Hochberg (B-H) adjustment for multiple testing. Genes are listed in alphabetical order.

### Profile comparisons

**Fig 2:**
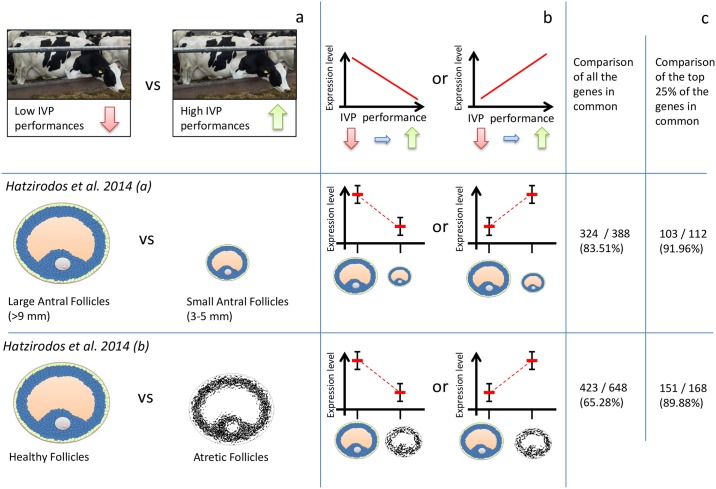
Comparison of expression pattern with previous studies by (Hatzirodos *et al*.(a, b)) [30, 31]. (**a**) Description of our analysis with respect to the differential expression analyses in (Hatzirodos *et al*.(a, b)) [30, 31]. The comparison from (Hatzirodos *et al*.(a)) [30] is inverted in this picture in order to show the similarity of the patterns that we observed. (**b**) Explanation of the comparisons. A gene is considered to have the same trend when its expression decreases or increases in both the studies for each comparison. (**c**) Number of genes observed with similar trend during the comparisons. The numbers refer to the genes with a similar trend with respect to the order described in Fig 2a and 2b.

We compared our gene expression pattern with direction or FCs of a published entire set of differentially expressed genes between healthy and early antral atretic follicles by (Hatzirodos *et al*.(b)) [31] regardless the statistical significance. In total, 423 out of 648 genes (65.28%) included in the comparison showed the same trend in our study and (Hatzirodos *et al*.(b)) [31]. In other words, we observed that 423 genes had increased (or decreased) expression profiles between healthy and atretic follicles corresponding to increased (or decreased) expression in cows with good IVP outcome demonstrating a positive relationship between early atresia and good IVP outcome. When selecting the subset of our gene list (only the top 25% of our gene list), we observed that 151 out of 168 genes show very high similarity in expression patterns or trend (89.88%) (Fig 2a, 2b and 2c).

When we compared our gene expression patterns for cows with good IVP outcome with the gene expression profiles of small versus medium large follicles as reported by (Hatzirodos *et al*.(a)) [30], 324 out of 388 genes (83.51%) showed the opposite trend, as explained in the methods and in the illustration below (Fig 2a, 2b and 2c). When considering 25% of our gene list, the percentage of genes with the opposite trend increased to 91.96% (103 out of 112 genes) (**Tables H and I in** S1 File).

In our experimental design, we compare IVP outcome from bad to good, whereas in the study by (Hatzirodos *et al*.(b)) [31], they compare profile changes from healthy versus atretic follicles. The relationship between these two studies is that IVP outcome is expected to be better for cows with a higher number of early atretic follicles. Based on this, Table 3 shows the candidate genes that can be used as potential biomarkers, as they have the same expression patterns with respect to good IVP outcome. In particular, when we looked into the significant lists of genes, three followed the same trend described in [31], while three showed opposite results (Table 3).

**Table 3:** Comparison of FCs of significant associated genes with expression changes identified between healthy and small atretic follicles.

Gene Name	FC in atretic follicles	BL rate (FC)	Kinetic (FC)	Morphology (FC)	Trend
*MEX3C*	-5	-0.12	-0.18	-0.10	Same
*GPRC5B*	+3.3	+0.14	+0.39	+0.24	Same
*ODF2L*	+5.8	+0.24	+0.33	+0.16	Same
*PRSS23*	+10.9	-0.11	-0.19	-0.12	Opposite
*DUSP7*	+3.6	-0.16	-0.21	-0.12	Opposite
*TNFAIP6*	+7.1	-0.15	-0.20	-0.12	Opposite

**Gene Name** = name of the gene, **FC** = fold change identified in [31], a positive change corresponds to upregulation in small atretic follicles, while negative values correspond to downregulation. **FC (BL rate, Kinetic, Morphology)** = FC obtained in our study for the corresponding IVP score, **Trend** = results of the comparison, same trend indicates that the gene follows the same trend as that described in (Hatzirodos *et al*.(b)) [31] (genes with the same trend resemble the presence of atretic follicles in cows with better IVP performance).

Contrarily, the comparison between our study and the gene expression analysis between small and medium or large follicles (Hatzirodos *et al*.(a)) [30] showed that most of the genes had the opposite trend. The relationship between these studies is that IVP outcome is expected to be better for cows with a higher number of small follicles. Table 4 shows the candidate genes that can be used as potential biomarkers, as they have opposite expression patterns with respect to good IVP outcome. Among the significant genes, 12 were associated with the follicle size (Table 4), including 11 that followed the opposite trend and one that showed the same trend.

**Table 4:** Comparison of FCs of significant genes with expression changes identified between small and medium or large antral follicles.

Gene Name	FC in large follicles	BL rate (FC)	Kinetic (FC)	Morphology (FC)	Trend
*RGN*	+9.8	-0.17	-0.26	-0.18	Opposite
*TNFAIP6*	+279.6	-0.15	-0.20	-0.12	Opposite
*SLMAP*	+5.1	-0.15	-0.25	-0.18	Opposite
*BTBD7*	+4.3	-0.16	-0.24	-0.15	Opposite
*BEX2*	+9	-0.18	-0.25	-0.18	Opposite
*SLC25A28*	+5.8	-0.15	-0.26	-0.18	Opposite
*CHCHD10*	+3.3	-0.15	-0.28	-0.19	Opposite
*PRSS23*	+48.5	-0.11	-0.19	-0.12	Opposite
*MAOA*	+3.1	-0.14	-0.24	-0.15	Opposite
*ITGB5*	+11.4	-0.15	-0.25	-0.16	Opposite
*ARHGAP18*	+20.5	-0.16	-0.24	-0.15	Opposite
*ODF2L*	+4.4	+0.24	+0.33	+0.16	Same

**Gene Name** = name of the gene, **FC** = fold change identified in [30], a positive change corresponds to upregulation in medium and large follicles, while negative values correspond to downregulation. **FC (BL rate, Kinetic, Morphology)** = FC obtained in our study for the corresponding IVP score, **Trend** = result of the comparison, same trend indicates that the gene follows the same trend as that described in (Hatzirodos *et al*.(a)) [30] (all the genes that show opposite behaviour resemble the expression profile of small follicles in cows with better IVP performance).

### Significantly enriched gene sets

Gene set enrichment analysis (GSEA) was performed to extract biological insight of the IVP process from the gene expression profiles. The top pathways (FDR<5%) are shown in (Table 5). As per the GSEA method recommendation, we also conducted the pathway analyses with the use of 25% FDR to reveal interesting biological mechanisms. The entire list of pathways with suggestive FDR <25% is reported in **Tables J-M in** S1 File.

**Table 5:** Significant enriched KEGG pathways (FDR <5%) identified from GSEA for all the IVP scores analysed.

IVP score	KEGG Pathway	Size	ES	NES	FDR q-val
BL rate	Progesterone-mediated oocyte maturation	61	-0.48	-1.82	4.73E-02
	Ribosome	45	0.62	2.34	0
	Pathogenic *Escherichia coli* infection	39	0.56	2.01	4.76E-03
Kinetic	Pathogenic *Escherichia coli* infection	39	0.65	2.38	0
	Cytokine-cytokine receptor interaction	54	0.51	2.02	7.60E-03
	Systemic *lupus erythematosus*	40	0.52	1.95	8.24E-03
	Spliceosome	110	0.43	1.95	9.33E-03
	Cell adhesion molecules (CAMs)	37	0.54	1.97	1.00E-02
	DNA replication	32	0.55	1.92	1.13E-02
	RIG-I-like receptor signalling pathway	41	0.49	1.80	1.96E-02
	Haematopoietic cell lineage	20	0.59	1.81	1.97E-02
	Viral myocarditis	22	0.58	1.81	2.25E-02
	Cytosolic DNA-sensing pathway	29	0.52	1.76	2.67E-02
	Complement and coagulation cascades	20	0.57	1.76	2.78E-02
	Antigen processing and presentation	30	0.50	1.70	4.11E-02
	Leukocyte transendothelial migration	53	0.43	1.71	4.14E-02
	Natural killer cell-mediated cytotoxicity	43	0.45	1.67	4.71E-02
Morphology	DNA replication	32	0.68	2.39	0
	Pathogenic *Escherichia coli* infection	39	0.63	2.27	0
	Cytokine-cytokine receptor interaction	54	0.52	2.12	2.08E-03
	Antigen processing and presentation	30	0.60	2.05	3.12E-03
	Haematopoietic cell lineage	20	0.62	1.96	4.79E-03
	Systemic lupus erythematosus	40	0.54	1.98	5.43E-03
	Natural killer cell-mediated cytotoxicity	43	0.51	1.93	8.36E-03
	Cysteine and methionine metabolism	27	0.57	1.90	9.31E-03
	RIG-I-like receptor signalling pathway	41	0.50	1.88	9.69E-03
	Cell adhesion molecules (CAMs)	37	0.50	1.85	1.16E-02
	Spliceosome	110	0.40	1.84	1.18E-02
	Leukocyte transendothelial migration	53	0.44	1.76	2.69E-02
	Cytosolic DNA-sensing pathway	29	0.50	1.72	3.80E-02
	Toll-like receptor signalling pathway	56	0.43	1.69	4.36E-02

**IVP score**: is the IVP score for which the pathway has been found enriched, **KEGG Pathway**: name of the KEGG gene set enriched, **Size**: total number of genes in the gene set, **ES**: Enrichment Score, **NES**: Normalized Enrichment Score, **FDR q-val**: False Discovery Rate associated with the entry.

For all the IVP scores, the analysis revealed positively enriched pathways involved in: DNA replication, cytokine-cytokine receptor interaction, pathways associated with the response to bacteria (pathogenic *Escherichia coli*, Toll-like receptor signalling pathway, antigen processing and presentation) and involvement of the immune system (natural killer cell-mediated cytotoxicity, leukocyte migration).

In terms of biological interpretation of the results, the following pathways (FDR <25%) are interesting: cell adhesion molecules (CAMs), gap junctions, fatty acid metabolism and p53 signalling. Furthermore, for BL rate we obtained the positive top-hit ribosome formation.

We obtained a set of pathways with negatively enriched score for BL rate involved in oocyte competence: progesterone mediated oocyte maturation and oocyte meiosis as well as pathways involved in cell cycle control, glycan biosynthesis and glycolysis, gluconeogenesis and sugar metabolism.

Gene Ontology (GO) term enrichment analysis confirmed the biological functions identified with the Kyoto Encyclopedia of Genes and Genomes (KEGG) pathways.

The lists of significant GO terms (FDR <25%) identified with the enrichment analysis for the GO terms are presented in **Tables N-Q in** S1 File.

### Ingenuity^®^ Pathway Analysis (IPA^®^) results

#### Upstream regulators

Among the top upstream regulators for BL rate, we identified many cytokines together with transcription regulators. The top five upstream regulators identified are: IFNA2, IFN Beta, thiocoraline, MAML1 and IL1RN (P value < 3.36E-4). ACOX1 is one of the top 10 identified upstream regulators predicted as significantly inhibited (Z-score = -2.00 and P value = 6.84 E-4).

The top upstream regulators identified for kinetic are involved in different functions (PRL, IRF7, TRIM24, UBA7 and ACKR2). The first two, PRL (Z-score = 2.45, P value = 7.57E-6) and IRF7 (Z-score = -2.18, P value = 8.86 E-6), were significantly predicted as activated, while IL1RN was predicted as inhibited (Z-score = -2.00, P value = 7.05 E-5).

**Fig 3:**
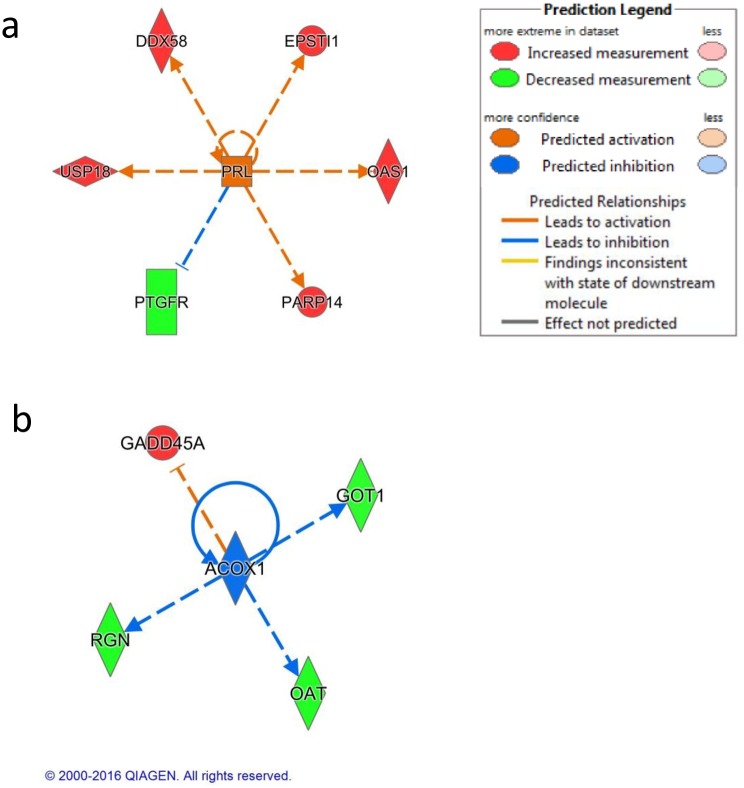
Upstream regulators with corresponding activation or inhibition predicted by IPA^®^. Upstream regulators and their targets displayed as a network of interactions with their respective expression trends. Node shapes represent functional classes; vertically rectangle for G-protein coupled receptor, squares for cytokines, vertically diamonds for enzymes, horizontally diamonds for peptidase, and circles for other. (a) PRL (predicted as activated), (b) ACOX1 (predicted as inhibited).

The upstream regulators identified for morphology are mainly involved in interferon regulation: IL17D, IFNK, IFNL1, IRF7 and IFNG (P value <1.62 E-4). The relevant upstream regulators are presented in Fig 3a and 3b.

#### Molecular and cellular function

The top five over-represented molecular and cellular functions identified for each IVP score together with the relatively enriched specific functions are represented in Table 6. When considering the most enriched functions that belong to these categories, we found: cell proliferation, cell development and homeostasis, necrosis, cell death and apoptosis. Only cell death identified for morphology showed a significant activation score (Z-score >2). Interestingly, inflammation of the body region (P value = 6.55 E-2, z-score = 2.21) and synthesis of reactive oxygen species (P value = 8.85E-2, z-score = -2.19), both involved in immune response, were predicted as being significantly inactivated. The complete list of the results from the enrichment analysis of molecular and cellular functions is shown in Tables R, S and T in S1 File.

**Table 6:** Molecular and cellular functions enriched in IPA^®^.

IVP score	Main category (-s*pecific function*)	Functional category	Z-score	B-H p-value
BL rate	Cellular Growth and Proliferation			4.52E-02-1.06E-01
	- *proliferation of cells*	proliferation	-1.23	4.52E-02
	- *proliferation of muscle cells*	proliferation	-1.11	4.52E-02
	Cellular Development			4.52E-02-1.14E-01
	- *differentiation of cells*	differentiation	0.02	5.69E-02
	- *proliferation of muscle cells*	proliferation	-1.11	4.52E-02
	Amino Acid Metabolism			4.52E-02-8.21E-02
	Gene Expression			4.52E-02-1.06E-01
	Cell Death and Survival			4.52E-02-1.14E-01
	- *necrosis of epithelial tissue*	necrosis	0	4.52E-02
	- *necrosis*	necrosis	0.8	6.62E-02
	- *apoptosis*	apoptosis	0.94	5.49E-02
	- *cell death of tumour cells*	cell death	0.82	6.37E-02
	- *cell death of epithelial cells*	cell death	0	6.73E-02
	- *cell death of cancer cells*	cell death	0.45	7.58E-02
	- *cell death*	cell death	1.45	7.58E-02
Kinetic	Cellular Development			2.62E-02-1.02E-01
	- *differentiation of cells*	differentiation	0.65	5.02E-02
	- *differentiation of connective tissue*	differentiation	0.58	7.21–02
	- *differentiation of leukocytes*	differentiation		7.64E-02
	Cellular Function and Maintenance			2.62E-02-8.96E-02
	- *cellular homeostasis*	homeostasis	-0.16	5.88E-02
	Cellular Growth and Proliferation			2.62E-02-1.02E-01
	- *proliferation of cells*	proliferation	-0.32	8.96E-02
	Gene Expression			2.62E-02-9.82E-02
	Carbohydrate Metabolism			2.62E-02-1.02E-01
Morphology	Cell Development			6.2E-03-7.33E-02
	Cellular Growth and Proliferation			6.2E-03-7.33E-02
	- *cellular growth and proliferation*	proliferation	-0.90	4.71–02
	Cell Signalling			6.2E-03-6.7E-02
	Carbohydrate Metabolism			6.2E-03-7.28E-02
	Cell Death and Survival			6.2E-03-4.76E-02
	- *cell death and survival*	cell death	2.32	3.39E-02
	- *cell death and survival*	apoptosis	1.68	4.44E-02

The table shows the top five main categories of molecular and cellular functions obtained in IPA^®^ for the BL rate, kinetic and morphology, together with significant specific function annotations (showing B-H P value <10% and including at least 5 genes). **Main category** = general functional class, **Specific function** = specific biological function, **Functional category** = general description of the function for the entry, **Z-score** = predicted level of activity (positive scores correspond to increased activity while negative scores correspond to decreased activity in relation to the increase in IVP performances), **B-H P value** = B-H adjusted *p* values for multiple testing.

#### Networks

**Fig 4:**
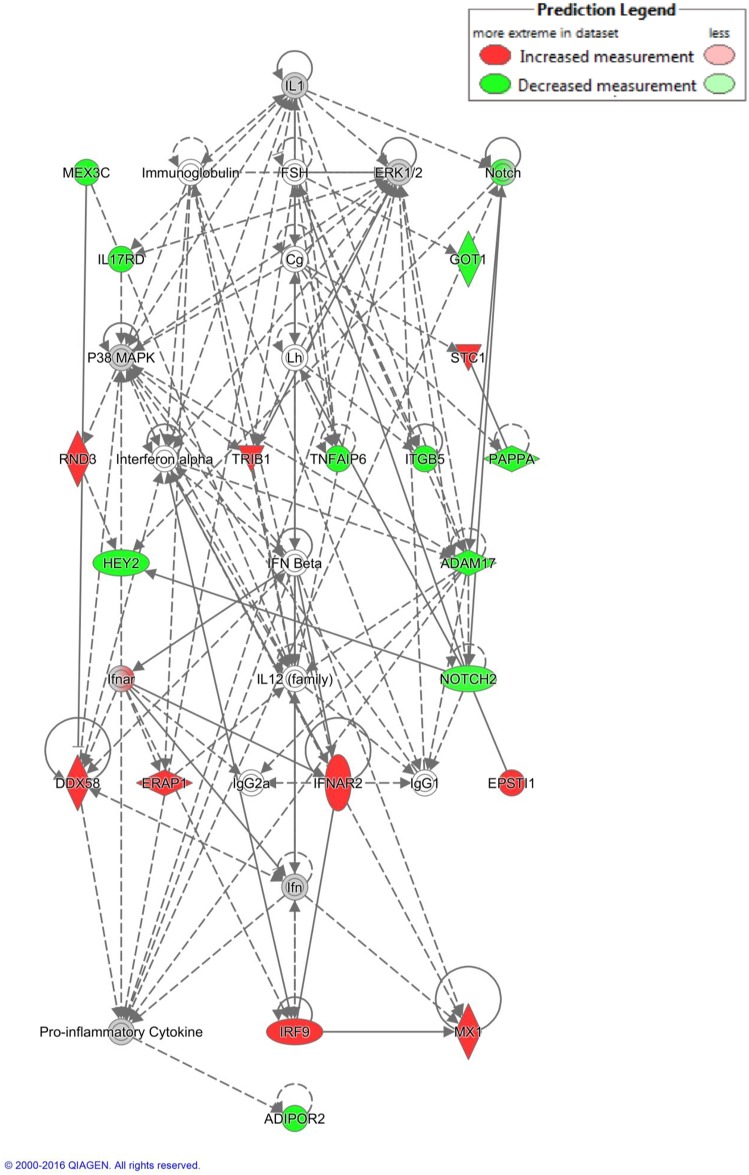
Network of interaction generated by IPA^®^ from the list of genes correlated with BL rate, organized by hierarchical structure. Node shapes represent functional classes; concentric circles for groups or complexes, vertically diamonds for enzymes, horizontally diamonds for peptidase, horizontally ellipses for transcriptional regulators or modulators, vertically ellipses for transmembrane receptor, inverted triangles for kinase and circles for other.

IPA^®^ generated interesting networks associated with different physiological and biological functions. We focused our analysis on a network involved in the control of the oestrous cycle and follicle development. We chose a network generated from the genes related to the BL rate with the highest number of genes, the highest score (Focus molecules = 19, score = 43) and in which both FSH and LH were included as nodes (Fig 4).

### Real-time PCR data validation

**Fig 5:**
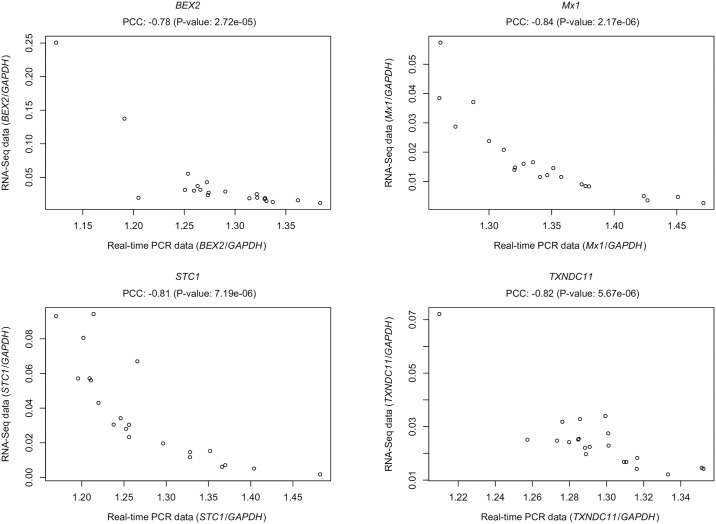
Scatter plots of the Real-time PCR data vs. RNA-Seq counts for the genes tested and Pearson Correlation Coefficient (PCC). The horizontal axis represents the ratio between the average Cp of the target gene and the average Cp of the reference (*GAPDH*) for all the samples. The vertical axis is the ratio between the RNA-Seq counts of the target gene and the reference gene for all the samples. PCC is the Pearson Correlation Coefficient between the Real time PCR data and the RNA-Seq data.

Real time PCR confirmed the RNA-Seq counts for the four genes tested. We obtained significant negative correlation (P value < 0.05) between Real-time PCR and RNA-Seq data for all the tests, indicating the concordance between the two methods in expressions patterns. The correlations between Real-time PCR and RNA-Seq data are presented in Fig 5.

## Discussion

In this study, we investigated the relationship between the individual cow granulosa cell transcriptomic profiles and IVP outcome as well as the possible effect of using semen from bulls with high versus low breeding values (NTM) on IVP outcome. Our study involved paired end RNA sequencing of granulosa cells isolated from follicular fluid and measuring the IVP scores or outcomes such as BL rate, morphology and kinetics. Then we used advanced statistical and bioinformatics methods to identify genes encoding potential biomarkers associated with IVP scores.

### Sperm effect

The model that we fit to test for the sperm effect did not show statistically significant effects of using sperm from bulls with high or low NTM index on the IVP performances. Hence, the use of high-NTM index bulls in IVP procedures does not have any negative effects and in fact showed a tendency for better IVP outcome. Further studies with a bigger population size and with reduced noise (for example with oestrous cycle synchronization) should preferably be performed in order to get enough statistical power to confirm and define a potential NTM effect of the sperm on IVP outcomes.

### Candidate genes

We found several candidate genes associated with IVP outcomes using the granulosa transcriptomic profiling and analyses. In order to interpret our results correctly we need to bear in mind that the RNA samples were derived from a heterogeneous pool of follicles. As a consequence, the most important candidate genes for follicle developmental stages like FSH receptor and P450 aromatase were expressed on average constantly across all the animals. LH receptor was excluded from the analysis during the filtering procedures, because lowly expressed (too low counts across samples). With respect to the multiple waves of follicular growth we expect to find a heterogeneous composition of our samples, including antral follicles of different dimensions as well as dominant and subordinate follicles at different developmental stages and in different stages of growth and atresia [49]. Thus, the genes that under these circumstances are correlated with IVP scores are good candidate genes, because their over- or underexpression profiles correlate with either a positive or negative outcome of the IVP procedure, despite the heterogeneity of the follicles. The IVP scores that we measured were scored independently of each other. For these reasons, we think that genes significantly correlated with all the scores are the most interesting genes to be tested as encoding potential biomarkers for selecting donor cows. Among the seven genes identified for all three scores, *Mx1* and *STC1* were positively correlated, while *BEX2*, *HEY2*, *RGN*, *TNFAIP6* and *TXNDC11* were negatively correlated to the IVP outcome. The expression patterns of four of these genes (*BEX2*, *Mx1*, *STC1*, *TXNDC11*) were validated with Real-time PCR. Most of these genes have previously been found to be involved in the control of follicle development and oocyte developmental potential.*Mx1* is involved in interferon signalling together with *IRF* and *IFNAR* (Interferon α/β receptor 1), which were both identified as being correlated only to BL rate. Prolactin response that involves interferon signaling has been related to optimal developmental competence of oocytes in cattle after 22 h- and 44 h of FSH coasting [27]. Interferon has been associated with developmental competence of oocytes in other species and in humans [50]. Correct concentrations of interferon and interleukins in human follicular fluid resulted were correlated to optimal oocyte developmental capacity. In mice, interferon-alpha (*IFN-α*) was upregulated in LH-treated preovulatory granulosa cells [51].

The protein product of *STC1* is secreted from the cells and has been found to control, in a paracrine way, the development of granulosa cells [52]. Furthermore, *STC1* is highly expressed in both *in vivo* and *in vitro* matured oocytes [53]. *STC1* probably plays an important role in a possible feedback loop between oocytes and granulosa cells.

*BEX2* has previously been found upregulated in large follicles rather than small ones [30]. *BEX2* acts as a negative regulator of apoptosis in the mitochondria and controls the G1 phase of the cell cycle [54].

*HEY2* encodes a transcriptional repressor, which is a downstream target of the *Notch* cell signaling system. The expression trend of *HEY2* is positively correlated to the expression of *NOTCH2* that was, in turn, negatively associated with BL rate (log2FC = -0.16, FDR = 0.05). In mice, *Notch* signaling has been shown to be extremely important to follicle and oocyte development and to exert a positive control of granulosa cell proliferation and a negative control of apoptosis [55]. Hence, as apoptosis can be expected during early atresia, the described relationship points towards a positive relationship between early atresia and good IVP outcome.

Finally, *RGN* has previously been found upregulated in large follicles rather than small ones [30] and has been described as being involved in the onset of follicular dominance and enhanced granulosa cell survival [56].

Transcript abundance of *TNFAIP6* has been associated with oocyte competence in cow granulosa cells after ovarian stimulation protocol with FSH where*TNFAIP6* resulted to be less transcribed in the competent group. In particular, *TNFAIP6* was found to be expressed more in granulosa cells of the incompetent group of associated oocytes [25]. *TNFAIP6* is an important component of the ECM as it has a hyaluronal-binding LINK domain [57].The ECM has a fundamental role during the follicle development [58]. ECM promotes cell survival and proliferation of granulosa cells in cattle [59]. Furthermore, N-glycan biosynthesis, one of the pathways identified with GSEA, is involved in ECM formation. The inhibitions of N-glycan biosynthesis has negative consequences on follicle development [60]. Hence, the described relationship again points towards a positive relationship between early atresia and good IVP outcome.

*TXNDC11* encodes a protein with the thioredoxin domain that might act as a redox regulator. *TXNDC11* has never been associated to oocyte competence in granulosa cells, although other thioredoxin proteins have been associated with the control of ovarian follicular atresia through scavenging action on reactive oxygen species [61].

Among the groups of gene significantly associated with only some of the IVP parameters, two genes, *OAT* negatively associated with BL rate and *OAS1* positively associated with kinetic score, deserve to be mentioned, because previously associated either with oocytes competence or follicle condition. *OAT* has been patented as a mammalian ovarian negative biomarker of oocyte competence [62] and the *OAS1* gene in oocytes has been associated with reduced fertility [63].

An important consideration during the selection of candidate genes encoding potential biomarkers is the subcellular localization of their protein products. Genes whose protein products are secreted in the extracellular space (including the follicular fluid and eventually blood plasma) can potentially be measured from the follicular fluid or even blood plasma during oocyte collection and used as biomarkers of IVP traits (**Tables E, F and G in** S1 File). Among the candidate genes described above, *STC1* is the only one whose protein product is secreted.

However, further studies are needed to test the correlation between the transcripts and the proteins that they encode for.

### Follicle size and follicle atresia hypothesis

The functional analysis of the seven candidate genes highlighted their potential involvement in the processes of atresia and follicular development. Similar evidences were obtained from the comparison of the expression pattern of our samples with the gene expression trend of healthy vs. early antral atretic follicles studied by Hatzirodos *et al*.(b) [31] and with the gene expression trend of small vs. medium and large follicles reported by Hatzirodos *et al*.(a) [30].

The comparison revealed that 65.28% of the genes identified as differentially expressed in early atretic follicles by Hatzirodos *et al*.(b) [31] showed the same trend in our study and this percentage increases to 89.88% considering the top 25% of the genes associated to BL rate. Similarly, 83.51% of the genes identified by Hatzirodos *et al*.(a) [30] in the comparison between small vs. medium and large follicles, showed opposite trend in our study, we observed that this percentage increases to 91.96% when we considered the top 25% genes related to BL rate.

In other words, in data good IVP outcome was positively correlated with early atresia and negatively correlated with follicle. Hence, very interestingly small early atretic follicles are most likely to result in good IVP outcome.

The evidence for this relationship is further sustained by the fact that several functional classes, biological pathways and regulator genes that are positively correlated with good IVP outcome also are associated with atresia: control of cell proliferation and development, cell death process, TP53 pathway and its regulator TRIM24, IFN-ϒ and PRL.

In details, we identified the pathway involved in control of cell proliferation for a set of genes negatively associated with IVP parameters. Interestingly, the biological function cell death was predicted as activated in cows with higher morphology score.

One of the most interesting pathways was that related to TP53. The TP53 pathway was enriched for genes positively associated with kinetic and morphology. TP53 induces apoptosis [64]. Interestingly, TP53 has been identified as being activated in when growing follicles enter the plateau phase and initiate atresia [27]. TRIM24 was identified as upstream regulator and it is involved in a key part of the TP53 mechanisms. TRIM24 promotes the degradation of TP53 and is primarily involved in TP53-induced apoptosis.

Many of the top upstream regulators identified for the blastocyst morphology score are related to the interferon pathway. Among these, IFN-ϒ has been found to enhance the apoptotic process in human, where the presence of IFN-ϒ has been observed specifically in atretic follicles [65, 66]. In the ovary, IFN-ϒ seems to be synthesized only by immune cells and not by granulosa cells [66]. This is in accordance with our results where IFN-γ was predicted as being activated, but its expression was not observed in our granulosa cell samples.

In our analysis, PRL is an upstream regulator of the genes that we identified associated to our IVP parameters. PRL was predicted as being activated in cows with good IVP outcome (Fig 3a). The PRL pathway is strictly associated with interferon signalling and its positive effect on oocyte competence has been widely studied [27].

It has earlier been noted that higher concentration of PRL in follicular fluid is positively correlated with early atresia. Lower concentration of PRL was associated with presence of pyknotic nuclei in granulosa cells which is an indicator of a late stage of cell death and follicular atresia [22, 67].

Treatments of PRL in rats *in vivo* resulted in an increased number of atretic follicles [68]. Furthermore, addition of PRL to *in vitro* maturation (IVM) media in co-culture with granulosa cells leads to an increase of embryos developed to the morula and blastocyst stages [69].

The relationships described above again indicate that a positive correlation exists between IVP outcome and early atresia.

Furthermore, we found that immune system was negatively correlated with IVP performances. We speculate that immune system activation is related to late atresia whereas the early atresia has not yet activated this type of a response. Again, this speculation supports the notion that early atresia, but not late atresia, is positively correlated with good IVP outcome. This theory is also sustained by observations reported earlier [70–73]. Hence, the percentage of embryos generated has earlier been positively correlated with early atresia whereas late atresia resulted in poor embryo yields[71]. This correlation has been attributed to differences in the development competence of the respective COCs. The developmental potential of the oocytes has earlier been reported as being positively correlated with granulosa cell apoptosis, follicle size and cumulus expansion as well as with the amount of follicular fluid granulosa cell apoptosis [70]. Granulosa cell apoptosis has been widely used to identify atretic follicles, but it has not yet been validated as a biomarker of oocyte competence [22].

Finally, previous studies also point to potential underlying cell biological mechanistic explanations of the positive correlation between good IVP outcome and early atresia. Hence, studies of the ultrastructure of COCs from dominant follicles approaching ovulation has clearly demonstrated that initial cumulus cell expansion and gradual retraction of the cumulus cell processes attached to the oocyte through the zona pellucida occur even prior to the LH peak [74]. These processes are associated with changes in the oocyte nucleus, i.e. the germinal vesicle, which develops undulations of the nuclear envelope likewise prior to the LH peak. After the LH peak these processes culminate in the resumption of meiosis and progress of cytoplasmic oocyte maturation over a 24 h period leading to ovulation. The same authors interestingly demonstrated that the above described sequence of processes can also be observed in COCs in the subordinate follicles of the follicular wave, i.e. follicles representing early atresia. Hence, in early atretic follicles, the COCs undergoes processes that mimic those seen in the dominant follicle approaching ovulation. Seen in this light, it is not surprising that COCs harvested from early atretic follicles may be better qualified for entering final maturation in vitro as they may be “primed” for the process. Interestingly, oocyte recovery post-FSH withdrawal period (“coasting”) has been demonstrated to increase IVP embryo yield [27]; an effect that is likely also to be based upon initiation of early atresia in the follicular pool.

It is important to keep in mind that even though all the animals were in the luteal phase of the estrous cycle, they were not synchronized. Hence, oocytes were collected at any given time point of the luteal phase and originated from a combination of growing and regressing follicular waves with the atretic follicles typically representing the subordinate follicular pool. The mechanistic hypothesis on the positive correlation between IVP outcome and early atresia needs further confirmation at single follicle level.

### Other biological mechanisms

The complexity of the IVP-related traits is represented in the numerous biological mechanisms that we found to be involved in the process. Together with the pathways involved in atresia we found many secondary pathways. The gene sets for the KEGG pathways, oocyte meiosis, progesterone-mediated oocyte maturation were enriched for genes negatively associated with BL rate that is in line with an early atresia condition while cytokine-cytokine receptor interaction pathway was enriched of genes positively correlated with good IVP outcome, which is not surprising as they represent key processes leading to oocyte competence [75]. We identified two cytokines: IL17D and IL1 (Fig 4) that are upstream regulators of the group of genes correlated respectively with morphology and BL rate. Interestingly, the expression of *IL17D* in granulosa cells as well as the presence of its encoded product in the follicular fluid has already been patented as a biomarker of oocyte competence in mammals [76]. Our data also showed a positive correlation between good IVP outcome and upregulation in the granulosa cells of the pathways gene expression, spliceosome and transcript maturation, as well as by the activation of the ribosome pathway indicating granulosa cell activity and function to be of significance for oocyte competence.

Upregulation of the pathogenic *Escherichia coli* pathway or Lipopolysaccharides effect likewise implies that activation of many subpathways important for the physiological function of the granulosa cells (e.g. gap junction, tight junction, adherent junction, axon guidance and regulation of actin cytoskeleton) are positively correlated with good IVP outcome.

In our study, the pathways for glycolysis and gluconeogenesis as well as galactose, mannose, fructose and fatty acid metabolism pathways were enriched by genes negatively associated with BL rate. The involvement of these pathways in oocyte development is supported by previous studies. It is known that granulosa cells provide the oocytes with glycolytic products and with energy production in order to support its correct development [77–80]. The pathway for fatty acid metabolism is also recognized as an important source of energy during oocytes maturation [81]. The protein ACOX1 was identified in our study to be an upstream regulator for a set of genes associated with BL rate. ACOX1 is involved in β-oxidation of fatty acids [82, 83]. In our dataset, *ACOX1* was also predicted as being inhibited in the BL rate from IPA^®^ (Fig 3b). The importance of beta oxidation and in particular of ACOX1 for fertility has been previously observed in a knockout study in mice [81, 84].

## Conclusions

In summary, to the best of our knowledge, this is the first study to have identified candidate genes encoding potential biomarkers of oocyte developmental competence in IVP using granulosa cells collected from pools of follicles at the individual cow level and implementing RNA-Seq technologies. In particular, we found evidence that good IVP outcome is positively correlated with early atresia. Our results provided stronger evidence of the involvement of our candidate genes in the IVP-related processes. Thus, we expect that the significant genes identified are good candidate genes for developing biomarkers for donor cow selection. Furthermore, we reported the most significant molecular pathways through which these candidate genes exert their effects on IVP outcomes. However, these candidate genes should be validated on a larger scale using OPU and IVP.

Future perspectives include the identification of eQTL for the candidate genes reported here and subsequent use in augmented GS procedures that utilize functional information, for example BLUP|GA (BLUP based on the genetic architecture) and sgBLUP (systems genomic BLUP) [1].

## Supporting information

S1 File Supplementary Tables: A-T. (DOCX)

S2 File Supplementary Figures: A-D. (DOCX)

S1 Text IVP procedure; Follicular cell purification and deep freezing procedure. (DOCX)
