# Supplementary material for: Identification of potential biomarkers in donor cows for in vitro embryo production by granulosa cell transcriptomics
Source: PLoS One. 2017 Apr 12;12(4):e0175464. doi: 10.1371/journal.pone.0175464 (PMC5389837; doi:10.1371/journal.pone.0175464)
Supplement: S1 File — (DOCX) [file pone.0175464.s001.docx]

# S1 File

# Identification of potential biomarkers in donor cows for *in vitro* embryo production by granulosa cell transcriptomics

**G. Mazzoni, S.M. Salleh, K. Freude, H.S. Pedersen, L. Stroebech, H. Callesen, P. Hyttel and H.N. Kadarmideen^*^**

* Corresponding author: hajak@dtu.dk

This PDF file included:

**Supplementary Tables: 1, 2 (a-c), 3 (a-c), 4 (a, b), 5 (a-h) and 6 (a-c)**

# Table A. Primer sequences and transcript accession numbers.

| Gene | Accession Number | Primer Sequence (Forward) | Primer (Sequence) Reverse | Product Size (bp) |
| --- | --- | --- | --- | --- |
| BEX2 | NM_001077034 | CCTAGAGGAAATCGCAGACG | TTCCCTCAGCTTTTCCATGA | 159 |
| Mx1 | NM_173940 | TGATCTCATCGACTCCCTGC | TGCCTTTCCACTCGTCTTCA | 199 |
| STC1 | NM_176669 | ATGATTGCCGAAGTCCAGGA | CCGTCTGCAGGATGTGAAAG | 241 |
| TXNDC11 | NM_001083420 | AGTCTCCTGCTGAACAACGA | GGATCAGGTGCCAGGGAC | 211 |
| GAPDH | NM_001034034 | AAGGCCATCACCATCTTCCA | TCACGCCCATCACAAACATG | 192 |

Accession number is the NCBI Reference Sequence

# Table B. List of genes associated to BL rate used in IPA.

| Symbol | Entrez ID | Log 2 FC | FDR | Entrez Gene Name | Location | Type(s) |
| --- | --- | --- | --- | --- | --- | --- |
| *ACSS3* | 531552 | -1.047 | 0.073 | acyl-CoA synthetase short-chain family member 3 | Cytoplasm | enzyme |
| *ADAM17* | 517541 | -1.048 | 0.02 | ADAM metallopeptidase domain 17 | Plasma Membrane | peptidase |
| *ADIPOR2* | 407234 | -1.033 | 0.048 | adiponectin receptor 2 | Plasma Membrane | other |
| *APOD* | 613972 | 0.766 | 0.078 | apolipoprotein D | Extracellular Space | transporter |
| *ARHGAP18* | 510743 | -1.226 | 0.023 | Rho GTPase activating protein 18 | Cytoplasm | other |
| *BCAT2* | 281643 | -1.367 | 0.009 | branched chain amino acid transaminase 2 | Cytoplasm | enzyme |
| *BMT2* | 538658 | -1.092 | 0.02 | base methyltransferase of 25S rRNA 2 homolog | Other | other |
| *BTBD6* | 100125777 | -1.025 | 0.078 | BTB domain containing 6 | Other | other |
| *BTBD7* | 100300409 | -1.245 | 0 | BTB domain containing 7 | Nucleus | other |
| *CASQ1* | 508394 | -1.172 | 0.046 | calsequestrin 1 | Cytoplasm | other |
| *CHCHD10* | 615846 | -1.181 | 0.036 | coiled-coil-helix-coiled-coil-helix domain containing 10 | Cytoplasm | other |
| *CHMP7* | 541288 | -1.059 | 0.031 | charged multivesicular body protein 7 | Cytoplasm | transporter |
| *CLMN* | 505010 | -0.945 | 0.036 | calmin (calponin-like, transmembrane) | Cytoplasm | other |
| *CTSL* | 515200 | 1.203 | 0.041 | cathepsin L | Cytoplasm | peptidase |
| *DDX58* | 504760 | 1.098 | 0.078 | DEXD/H-box helicase 58 | Cytoplasm | enzyme |
| *DHRS12* | 507276 | -1.183 | 0.047 | dehydrogenase/reductase (SDR family) member 12 | Nucleus | other |
| *DUSP7* | 785016 | -1.292 | 0.02 | dual specificity phosphatase 7 | Cytoplasm | phosphatase |
| *EPSTI1* | 614555 | 1.251 | 0.02 | epithelial stromal interaction 1 (breast) | Other | other |
| *ERAP1* | 514617 | 1.085 | 0.078 | endoplasmic reticulum aminopeptidase 1 | Extracellular Space | peptidase |
| *FBXL7* | 518806 | -1.431 | 0.005 | F-box and leucine-rich repeat protein 7 | Cytoplasm | enzyme |
| *FBXO28* | 537745 | -1.005 | 0.095 | F-box protein 28 | Other | other |
| *FIBIN* | 507975 | -1.08 | 0.047 | fin bud initiation factor homolog (zebrafish) | Cytoplasm | other |
| *GADD45A* | 505463 | 1.143 | 0.029 | growth arrest and DNA damage inducible alpha | Nucleus | other |
| *GOT1* | 281206 | -0.876 | 0.02 | glutamic-oxaloacetic transaminase 1 | Cytoplasm | enzyme |
| *HEY2* | 538619 | -1.327 | 0.014 | hes related family bHLH transcription factor with YRPW motif 2 | Nucleus | transcription regulator |
| *IAH1* | 614320 | -1.089 | 0.051 | isoamyl acetate-hydrolyzing esterase 1 homolog | Cytoplasm | other |
| *IFNAR2* | 282258 | 1.021 | 0.047 | interferon alpha and beta receptor subunit 2 | Plasma Membrane | transmembrane receptor |
| *IL17RD* | 782214 | -1.016 | 0.078 | interleukin 17 receptor D | Cytoplasm | other |
| *IRF9* | 509855 | 1.195 | 0.02 | interferon regulatory factor 9 | Nucleus | transcription regulator |
| *ITGB5* | 282564 | -1.138 | 0.047 | integrin subunit beta 5 | Plasma Membrane | other |
| *JADE3* | 506664 | -0.878 | 0.01 | jade family PHD finger 3 | Nucleus | other |
| *KBTBD7* | 539110 | -0.848 | 0.078 | kelch repeat and BTB domain containing 7 | Cytoplasm | other |
| *KCTD20* | 514967 | -0.582 | 0.069 | potassium channel tetramerization domain containing 20 | Cytoplasm | other |
| *KIAA2022* | 512493 | -0.886 | 0.092 | KIAA2022 | Other | enzyme |
| *LIMD2* | 508942 | 1.081 | 0.085 | LIM domain containing 2 | Other | other |
| *MAOA* | 281293 | -1.112 | 0.046 | monoamine oxidase A | Cytoplasm | enzyme |
| *MEX3C* | 529318 | -0.97 | 0.02 | mex-3 RNA binding family member C | Cytoplasm | other |
| *MS4A8* | 415111 | -1.238 | 0.017 | membrane spanning 4-domains A8 | Plasma Membrane | other |
| *MX1* | 280872 | 1.079 | 0.047 | MX dynamin like GTPase 1 | Cytoplasm | enzyme |
| *NOTCH2* | 513730 | -0.815 | 0.051 | notch 2 | Plasma Membrane | transcription regulator |
| *NTRK1* | 353111 | -0.769 | 0.078 | neurotrophic receptor tyrosine kinase 1 | Plasma Membrane | kinase |
| *OAT* | 505323 | -1.291 | 0.02 | ornithine aminotransferase | Cytoplasm | enzyme |
| *ODF2L* | 533508 | 1.559 | 0.001 | outer dense fiber of sperm tails 2 like | Cytoplasm | other |
| *P3H2* | 511799 | 1.022 | 0.089 | prolyl 3-hydroxylase 2 | Cytoplasm | enzyme |
| *PAPPA* | 282647 | -1.123 | 0.028 | pappalysin 1 | Extracellular Space | peptidase |
| *PPM1K* | 540329 | -0.987 | 0.078 | protein phosphatase, Mg2+/Mn2+ dependent 1K | Cytoplasm | phosphatase |
| *PRPF8* | 507371 | -0.541 | 0.073 | pre-mRNA processing factor 8 | Nucleus | other |
| *PRSS23* | 538575 | -0.858 | 0.029 | protease, serine 23 | Extracellular Space | peptidase |
| *PTGFR* | 282020 | -0.879 | 0.066 | prostaglandin F receptor | Plasma Membrane | G-protein coupled receptor |
| *RDH14* | 505949 | -0.835 | 0.02 | retinol dehydrogenase 14 (all-trans/9-cis/11-cis) | Cytoplasm | enzyme |
| *RGN* | 280910 | -1.334 | 0.002 | regucalcin | Nucleus | enzyme |
| *RND3* | 783333 | 0.961 | 0.096 | Rho family GTPase 3 | Cytoplasm | enzyme |
| *SCD5* | 617419 | -1.122 | 0.07 | stearoyl-CoA desaturase 5 | Cytoplasm | enzyme |
| *SLC25A28* | 538529 | -1.2 | 0.02 | solute carrier family 25 member 28 | Cytoplasm | transporter |
| *SLMAP* | 529366 | -1.151 | 0.014 | sarcolemma associated protein | Plasma Membrane | other |
| *SMIM1* | 100302586 | -1.068 | 0.097 | small integral membrane protein 1 (Vel blood group) | Cytoplasm | other |
| *STC1* | 338078 | 1.018 | 0.047 | stanniocalcin 1 | Extracellular Space | kinase |
| *SUGCT* | 100125578 | -1.44 | 0.005 | succinyl-CoA:glutarate-CoA transferase | Cytoplasm | enzyme |
| *TMOD1* | 780784 | 1.45 | 0.005 | tropomodulin 1 | Cytoplasm | enzyme |
| *TNFAIP6* | 493710 | -1.129 | 0 | TNF alpha induced protein 6 | Extracellular Space | other |
| *TNKS* | 535030 | -0.949 | 0.047 | tankyrase | Nucleus | enzyme |
| *TRIB1* | 521857 | 1.079 | 0.061 | tribbles pseudokinase 1 | Cytoplasm | kinase |
| *TXNDC11* | 513509 | -1.31 | 0.006 | thioredoxin domain containing 11 | Cytoplasm | enzyme |
| *ZFHX3* | 510844 | -0.931 | 0.051 | zinc finger homeobox 3 | Nucleus | transcription regulator |

**Symbol** = gene name (HGNC), **Entrez ID**= cattle Entrez ID, **Log2FC**=log 2 Fold Change identified for BL rate, **FDR** = P Value adjusted (B-H correction), **Entrez Gene Name**= Entrez extended gene name, **Location**=sub-cellular localization annotation, **Type**(s) = type of protein.

# Table C. List of genes associated to kinetic used in IPA.

| Symbol | Entrez ID | Log 2 FC | FDR | Entrez Gene Name | Location | Type(s) |
| --- | --- | --- | --- | --- | --- | --- |
| *ATP10D* | 536495 | -0.374 | 0.062 | ATPase phospholipid transporting 10D (putative) | Cytoplasm | transporter |
| *BTBD7* | 100300409 | -0.403 | 0.019 | BTB domain containing 7 | Nucleus | other |
| *CHCHD10* | 615846 | -0.469 | 0.032 | coiled-coil-helix-coiled-coil-helix domain containing 10 | Cytoplasm | other |
| *CHMP7* | 541288 | -0.392 | 0.07 | charged multivesicular body protein 7 | Cytoplasm | transporter |
| *CTSL* | 515200 | 0.511 | 0.021 | cathepsin L | Cytoplasm | peptidase |
| *DDX58* | 504760 | 0.467 | 0.034 | DEXD/H-box helicase 58 | Cytoplasm | enzyme |
| *DESI1* | 614655 | -0.41 | 0.082 | desumoylating isopeptidase 1 | Other | other |
| *DHRS12* | 507276 | -0.466 | 0.038 | dehydrogenase/reductase (SDR family) member 12 | Nucleus | other |
| *EPSTI1* | 614555 | 0.51 | 0.017 | epithelial stromal interaction 1 (breast) | Other | other |
| *FBXL7* | 518806 | -0.466 | 0.036 | F-box and leucine-rich repeat protein 7 | Cytoplasm | enzyme |
| *GPRC5B* | 516334 | 0.477 | 0.032 | G protein-coupled receptor class C group 5 member B | Plasma Membrane | G-protein coupled receptor |
| *HEY2* | 538619 | -0.522 | 0.017 | hes related family bHLH transcription factor with YRPW motif 2 | Nucleus | transcription regulator |
| *IAH1* | 614320 | -0.419 | 0.066 | isoamyl acetate-hydrolyzing esterase 1 homolog | Cytoplasm | other |
| *ITGB5* | 282564 | -0.409 | 0.09 | integrin subunit beta 5 | Plasma Membrane | other |
| *JADE3* | 506664 | -0.328 | 0.032 | jade family PHD finger 3 | Nucleus | other |
| *Mkrn1* | 514986 | -0.342 | 0.086 | makorin ring finger protein 1 | Plasma Membrane | enzyme |
| *MX1* | 280872 | 0.63 | 0 | MX dynamin like GTPase 1 | Cytoplasm | enzyme |
| *NOTCH2* | 513730 | -0.307 | 0.086 | notch 2 | Plasma Membrane | transcription regulator |
| *OAS1* | 654488 | 0.461 | 0.019 | 2'-5'-oligoadenylate synthetase 1 | Cytoplasm | enzyme |
| *OSTM1* | 518522 | 0.332 | 0.062 | osteopetrosis associated transmembrane protein 1 | Cytoplasm | other |
| *PARP14* | 540789 | 0.487 | 0.017 | poly(ADP-ribose) polymerase family member 14 | Cytoplasm | other |
| *PDGFB* | 788643 | 0.426 | 0.089 | platelet derived growth factor subunit B | Extracellular Space | growth factor |
| *PRDM1* | 538384 | 0.429 | 0.086 | PR domain 1 | Nucleus | transcription regulator |
| *PRSS23* | 538575 | -0.305 | 0.081 | protease, serine 23 | Extracellular Space | peptidase |
| *PTGFR* | 282020 | -0.346 | 0.086 | prostaglandin F receptor | Plasma Membrane | G-protein coupled receptor |
| *REV3L* | 517422 | -0.332 | 0.08 | REV3 like, DNA directed polymerase zeta catalytic subunit | Nucleus | enzyme |
| *RGN* | 280910 | -0.427 | 0.022 | regucalcin | Nucleus | enzyme |
| *RSPRY1* | 538571 | -0.307 | 0.034 | ring finger and SPRY domain containing 1 | Other | other |
| *SH3BP2* | 617344 | 0.423 | 0.086 | SH3 domain binding protein 2 | Cytoplasm | other |
| *SH3D19* | 535511 | -0.345 | 0.032 | SH3 domain containing 19 | Plasma Membrane | other |
| *SLC25A28* | 538529 | -0.444 | 0.036 | solute carrier family 25 member 28 | Cytoplasm | transporter |
| *SLMAP* | 529366 | -0.409 | 0.041 | sarcolemma associated protein | Plasma Membrane | other |
| *STC1* | 338078 | 0.647 | 0 | stanniocalcin 1 | Extracellular Space | kinase |
| *SUGCT* | 100125578 | -0.502 | 0.022 | succinyl-CoA:glutarate-CoA transferase | Cytoplasm | enzyme |
| *TC2N* | 534419 | -0.364 | 0.031 | tandem C2 domains, nuclear | Nucleus | transporter |
| *TMED5* | 534351 | 0.262 | 0.09 | transmembrane p24 trafficking protein 5 | Cytoplasm | other |
| *TMOD1* | 780784 | 0.478 | 0.032 | tropomodulin 1 | Cytoplasm | enzyme |
| *TMOD2* | 532085 | 0.477 | 0.034 | tropomodulin 2 | Cytoplasm | other |
| *TNFAIP6* | 493710 | -0.329 | 0.003 | TNF alpha induced protein 6 | Extracellular Space | other |
| *TNKS* | 535030 | -0.392 | 0.025 | tankyrase | Nucleus | enzyme |
| *TXNDC11* | 513509 | -0.486 | 0.018 | thioredoxin domain containing 11 | Cytoplasm | enzyme |
| *USP18* | 515202 | 0.479 | 0.025 | ubiquitin specific peptidase 18 | Cytoplasm | peptidase |
| *WDYHV1* | 510463 | -0.387 | 0.082 | WDYHV motif containing 1 | Cytoplasm | other |

**Symbol** = gene name (HGNC), **Entrez ID**= cattle Entrez ID, **Log2FC**=log 2 Fold Change identified for kinetic, **FDR** = P Value adjusted (B-H correction), **Entrez Gene Name**= Entrez extended gene name, **Location**=sub-cellular localization annotation, **Type**(s) = type of protein.

# Table D. List of genes associated to morphology used in IPA.

| **Symbol** | **Entrez ID** | **Log 2 FC** | **FDR** | **Entrez Gene Name** | **Location** | **Type(s)** |
| --- | --- | --- | --- | --- | --- | --- |
| *CHMP7* | 541288 | -0.283 | 0.1 | charged multivesicular body protein 7 | Cytoplasm | transporter |
| *HEY2* | 538619 | -0.358 | 0.044 | hes related family bHLH transcription factor with YRPW motif 2 | Nucleus | transcription regulator |
| *LAMB2* | 520167 | -0.235 | 0.056 | laminin subunit beta 2 | Extracellular Space | enzyme |
| *Mkrn1* | 514986 | -0.272 | 0.045 | makorin ring finger protein 1 | Plasma Membrane | enzyme |
| *MX1* | 280872 | 0.33 | 0.044 | MX dynamin like GTPase 1 | Cytoplasm | enzyme |
| *OAS1* | 654488 | 0.28 | 0.098 | 2'-5'-oligoadenylate synthetase 1 | Cytoplasm | enzyme |
| *PARP14* | 540789 | 0.332 | 0.044 | poly(ADP-ribose) polymerase family member 14 | Cytoplasm | other |
| *RGN* | 280910 | -0.29 | 0.045 | regucalcin | Nucleus | enzyme |
| *STC1* | 338078 | 0.351 | 0.025 | stanniocalcin 1 | Extracellular Space | kinase |
| *TC2N* | 534419 | -0.297 | 0.008 | tandem C2 domains, nuclear | Nucleus | transporter |
| *TNFAIP6* | 493710 | -0.19 | 0.045 | TNF alpha induced protein 6 | Extracellular Space | other |
| *TXNDC11* | 513509 | -0.324 | 0.045 | thioredoxin domain containing 11 | Cytoplasm | enzyme |

**Symbol** = gene name (HGNC), **Entrez ID**= cattle Entrez ID, **Log2FC**=log 2 Fold Change identified for morphology, **FDR** = P Value adjusted (B-H correction), **Entrez Gene Name**= Entrez extended gene name , **Location**=sub-cellular localization annotation, **Type**(s) = type of protein.

# Table E. Genes significantly related to BL rate

| Associated Gene Name | log2 FC | SE | stat | P value | FDR |
| --- | --- | --- | --- | --- | --- |
| *RPS26 (paralogue)* | -0.57 | 0.05 | -10.59 | 3.28E-26 | 3.39E-22 |
| *ODF2L* | 0.31 | 0.06 | 5.12 | 3.00E-07 | 6.96E-04 |
| *TMOD1* | 0.29 | 0.06 | 4.65 | 3.38E-06 | 4.63E-03 |
| *BEX2* | -0.29 | 0.06 | -5.10 | 3.36E-07 | 6.96E-04 |
| *SUGCT* | -0.29 | 0.06 | -4.58 | 4.59E-06 | 5.28E-03 |
| *FBXL7* | -0.29 | 0.06 | -4.63 | 3.58E-06 | 4.63E-03 |
| *BCAT2* | -0.27 | 0.06 | -4.42 | 9.93E-06 | 9.34E-03 |
| *RGN* | -0.27 | 0.05 | -4.89 | 1.01E-06 | 1.74E-03 |
| *HEY2* | -0.27 | 0.06 | -4.27 | 1.94E-05 | 1.44E-02 |
| *TXNDC11* | -0.26 | 0.06 | -4.54 | 5.71E-06 | 5.91E-03 |
| *DUSP7* | -0.26 | 0.06 | -4.11 | 3.90E-05 | 2.00E-02 |
| *OAT* | -0.26 | 0.06 | -4.11 | 4.02E-05 | 2.00E-02 |
| *ARHGAP28* | 0.26 | 0.06 | 4.06 | 4.87E-05 | 2.00E-02 |
| *EPSTI1* | 0.25 | 0.06 | 4.07 | 4.75E-05 | 2.00E-02 |
| *BTBD7* | -0.25 | 0.04 | -6.08 | 1.17E-09 | 4.03E-06 |
| *MS4A8* | -0.25 | 0.06 | -4.22 | 2.48E-05 | 1.71E-02 |
| *ARHGAP18* | -0.25 | 0.06 | -4.01 | 6.05E-05 | 2.32E-02 |
| *CTSL ** | 0.24 | 0.06 | 3.81 | 1.38E-04 | 4.07E-02 |
| *SLC25A28* | -0.24 | 0.06 | -4.10 | 4.06E-05 | 2.00E-02 |
| *IRF9* | 0.24 | 0.06 | 4.07 | 4.75E-05 | 2.00E-02 |
| *TMEM254* | -0.24 | 0.06 | -3.88 | 1.06E-04 | 3.43E-02 |
| *DHRS12* | -0.24 | 0.06 | -3.75 | 1.74E-04 | 4.65E-02 |
| *CHCHD10* | -0.24 | 0.06 | -3.86 | 1.14E-04 | 3.57E-02 |
| *CASQ1* | -0.23 | 0.06 | -3.78 | 1.59E-04 | 4.57E-02 |
| *SLMAP* | -0.23 | 0.05 | -4.29 | 1.76E-05 | 1.40E-02 |
| *GADD45A* | 0.23 | 0.06 | 3.93 | 8.46E-05 | 2.92E-02 |
| *ITGB5* | -0.23 | 0.06 | -3.73 | 1.90E-04 | 4.65E-02 |
| *TNFAIP6 ** | -0.23 | 0.04 | -6.12 | 9.08E-10 | 4.03E-06 |
| *PAPPA* | -0.22 | 0.06 | -3.95 | 7.70E-05 | 2.85E-02 |
| *MAOA* | -0.22 | 0.06 | -3.77 | 1.64E-04 | 4.59E-02 |
| *C7orf60* | -0.22 | 0.05 | -4.11 | 3.90E-05 | 2.00E-02 |
| *FIBIN ** | -0.22 | 0.06 | -3.73 | 1.94E-04 | 4.65E-02 |
| *Mx1* | 0.22 | 0.06 | 3.73 | 1.92E-04 | 4.65E-02 |
| *CHMP7* | -0.21 | 0.05 | -3.91 | 9.20E-05 | 3.07E-02 |
| *ADAM17* | -0.21 | 0.05 | -4.05 | 5.02E-05 | 2.00E-02 |
| *ADIPOR2* | -0.21 | 0.06 | -3.71 | 2.10E-04 | 4.84E-02 |
| *IFNAR2 ** | 0.20 | 0.05 | 3.73 | 1.89E-04 | 4.65E-02 |
| *STC1 ** | 0.20 | 0.05 | 3.72 | 1.98E-04 | 4.65E-02 |
| *MEX3C* | -0.19 | 0.05 | -4.09 | 4.29E-05 | 2.00E-02 |
| *TNKS* | -0.19 | 0.05 | -3.75 | 1.76E-04 | 4.65E-02 |
| *CLMN* | -0.19 | 0.05 | -3.85 | 1.20E-04 | 3.64E-02 |
| *JADE3* | -0.18 | 0.04 | -4.38 | 1.18E-05 | 1.02E-02 |
| *GOT1* | -0.18 | 0.04 | -4.09 | 4.40E-05 | 2.00E-02 |
| *PRSS23 ** | -0.17 | 0.04 | -3.94 | 8.00E-05 | 2.86E-02 |
| *RDH14* | -0.17 | 0.04 | -4.07 | 4.62E-05 | 2.00E-02 |

Genes significantly correlated with BL rate. **Log2 FC**: amount of doubling in gene expression level for each 20% increase in BL rate, **SE**: standard error associated with the log2foldchange, **stat**: Wald statistic**, P value**: nominal P value associated to the estimate, **FDR**: BH adjustment for multiple testing.

The symbol * indicates genes whose protein products are localized in the extracellular space or secreted following GO term annotation in Uniprot

# Table F. Genes significantly related to Kinetic

| Associated Gene Name | Log 2FC | SE | stat | P value | FDR |
| --- | --- | --- | --- | --- | --- |
| *RPS26* | -0.97 | 0.10 | -9.79 | 1.29E-22 | 1.41E-18 |
| *STC1 ** | 0.65 | 0.11 | 5.73 | 1.03E-08 | 5.63E-05 |
| *Mx1* | 0.63 | 0.12 | 5.44 | 5.23E-08 | 1.90E-04 |
| *HEY2* | -0.52 | 0.12 | -4.44 | 9.17E-06 | 1.67E-02 |
| *CTSL ** | 0.51 | 0.12 | 4.25 | 2.15E-05 | 2.13E-02 |
| *EPSTI1* | 0.51 | 0.12 | 4.40 | 1.07E-05 | 1.67E-02 |
| *SUGCT* | -0.50 | 0.12 | -4.23 | 2.39E-05 | 2.17E-02 |
| *PARP14* | 0.49 | 0.11 | 4.42 | 9.77E-06 | 1.67E-02 |
| *TXNDC11* | -0.49 | 0.11 | -4.35 | 1.33E-05 | 1.81E-02 |
| *USP18* | 0.48 | 0.12 | 4.16 | 3.23E-05 | 2.48E-02 |
| *TMOD1* | 0.48 | 0.12 | 4.01 | 6.05E-05 | 3.24E-02 |
| *GPRC5B ** | 0.48 | 0.12 | 4.00 | 6.41E-05 | 3.24E-02 |
| *TMOD2* | 0.48 | 0.12 | 3.97 | 7.21E-05 | 3.40E-02 |
| *PTPN13* | -0.22 | 0.12 | -3.96 | 0.00 | 3.40E-02 |
| *CHCHD10* | -0.47 | 0.12 | -4.02 | 5.92E-05 | 3.24E-02 |
| *DDX58* | 0.47 | 0.12 | 3.95 | 7.97E-05 | 3.40E-02 |
| *DHRS12* | -0.47 | 0.12 | -3.89 | 1.01E-04 | 3.81E-02 |
| *FBXL7* | -0.47 | 0.12 | -3.91 | 9.34E-05 | 3.63E-02 |
| *OAS1* | 0.46 | 0.11 | 4.32 | 1.53E-05 | 1.85E-02 |
| *SLC25A28* | -0.44 | 0.11 | -3.92 | 8.86E-05 | 3.57E-02 |
| *RGN* | -0.43 | 0.10 | -4.21 | 2.60E-05 | 2.18E-02 |
| *BEX2* | -0.42 | 0.10 | -4.04 | 5.24E-05 | 3.17E-02 |
| *SLMAP* | -0.41 | 0.11 | -3.86 | 1.14E-04 | 4.13E-02 |
| *BTBD7* | -0.40 | 0.09 | -4.29 | 1.75E-05 | 1.90E-02 |
| *TNKS* | -0.39 | 0.09 | -4.14 | 3.41E-05 | 2.48E-02 |
| *TC2N* | -0.36 | 0.09 | -4.07 | 4.62E-05 | 3.14E-02 |
| *SH3D19* | -0.35 | 0.09 | -3.99 | 6.54E-05 | 3.24E-02 |
| *TNFAIP6 ** | -0.33 | 0.07 | -4.89 | 1.03E-06 | 2.80E-03 |
| *JADE3* | -0.33 | 0.08 | -4.06 | 4.95E-05 | 3.17E-02 |
| *RSPRY1 ** | -0.31 | 0.08 | -3.94 | 8.11E-05 | 3.40E-02 |

Genes significantly correlated with kinetic. **Log2 FC**: amount of doubling in gene expression level for an increase of 1 unit in Kinetic score, **SE**: standard error associated with the log2foldchange, **stat**: Wald statistic, **P value**: nominal P value associated to the estimate, **FDR**: BH adjustment for multiple testing.

The symbol * indicates genes whose protein products are localized in the extracellular space or secreted following GO term annotation in Uniprot.

# Table G. Genes significantly related to Morphology

| Associated Gene Name | log2 FC | SE | stat | P Value | FDR |
| --- | --- | --- | --- | --- | --- |
| *HEY2* | -0.36 | 0.08 | -4.29 | 1.82E-05 | 4.40E-02 |
| *STC1** | 0.35 | 0.08 | 4.51 | 6.49E-06 | 2.47E-02 |
| *PARP14* | 0.33 | 0.08 | 4.19 | 2.75E-05 | 4.40E-02 |
| *Mx1* | 0.33 | 0.08 | 4.18 | 2.89E-05 | 4.40E-02 |
| *TXNDC11* | -0.32 | 0.08 | -4.02 | 5.81E-05 | 4.50E-02 |
| *TC2N* | -0.30 | 0.06 | -4.88 | 1.06E-06 | 8.10E-03 |
| *RGN* | -0.29 | 0.07 | -4.04 | 5.32E-05 | 4.50E-02 |
| *BEX2* | -0.28 | 0.07 | -4.03 | 5.64E-05 | 4.50E-02 |
| *MKRN1* | -0.27 | 0.07 | -4.08 | 4.57E-05 | 4.50E-02 |
| *TNFAIP6** | -0.19 | 0.05 | -4.02 | 5.91E-05 | 4.50E-02 |

Genes significantly correlated with Morphology. **Log2 FC**: amount of doubling in gene expression level for an increase of 1 unit in Morphology score, **SE**: standard error associated with the log2foldchange, **stat**: Wald statistic, **P value**: nominal P value associated to the estimate, **FDR**: BH adjustment for multiple testing.

The symbol * indicates genes whose protein products are localized in the extracellular space or secreted following GO term annotation in Uniprot.

# Table H. Comparison of the regression coefficient obtained for IVP scores with the differential expression profile obtained from comparison of healthy follicles to small atretic follicles (Hatzirodos et al. 2014b).

| **Gene Name** | **BL rate (FC)** | **Kinetic(FC)** | **Morphology (FC)** | **FC (healthy to small atretic follicles)** | **trend** |  |
| --- | --- | --- | --- | --- | --- | --- |
| *ABCD1* | -0.07 | -0.10 | -0.08 | 4.2 | D |  |
| *ACAD10* | -0.03 | -0.07 | -0.06 | 3.2 | D |  |
| *ACSM1* | 0.04 | 0.27 | 0.22 | 3.1 | D |  |
| *ALDH1L2* | 0.04 | 0.10 | 0.06 | 3.3 | D |  |
| *ALG3* | -0.05 | -0.09 | -0.05 | 7.8 | D |  |
| *ALG5* | -0.03 | -0.07 | -0.05 | 3.2 | D |  |
| *AMH* | -0.01 | -0.01 | -0.01 | 4.2 | D |  |
| *ANAPC5* | -0.04 | -0.10 | -0.09 | 4.4 | D |  |
| *ANKRD17* | 0.00 | -0.01 | -0.01 | 3.1 | D |  |
| *AP4B1* | 0.04 | 0.02 | 0.03 | 3.2 | D |  |
| *ASF1B* | -0.05 | 0.05 | 0.06 | 3.7 | D |  |
| *ASPM* | -0.10 | -0.14 | -0.07 | 3.2 | D |  |
| *ATP13A2* | -0.06 | -0.05 | -0.02 | 3.8 | D |  |
| *ATP2B4* | -0.02 | 0.01 | 0.00 | 4.4 | D |  |
| *AURKB* | -0.03 | 0.03 | 0.04 | 3 | D |  |
| *B3GNT3* | 0.03 | 0.04 | 0.01 | 3.1 | D |  |
| *BCL9* | -0.03 | -0.08 | -0.06 | 4 | D |  |
| *BCOR* | -0.07 | -0.09 | -0.05 | 3.5 | D |  |
| *BCORL1* | -0.01 | -0.01 | 0.00 | 3.2 | D |  |
| *BIRC5* | -0.07 | -0.07 | -0.05 | 3.2 | D |  |
| *BUB1* | -0.07 | -0.06 | -0.03 | 3.9 | D |  |
| *BUB1B* | -0.07 | -0.05 | -0.02 | 4.2 | D |  |
| *C6orf47* | -0.02 | -0.02 | -0.01 | 3.3 | D |  |
| *CA14* | 0.01 | 0.04 | 0.03 | 6.8 | D |  |
| *CA5B* | -0.10 | -0.16 | -0.12 | 3.2 | D |  |
| *CC2D1B* | 0.00 | 0.11 | 0.13 | 3.2 | D |  |
| *CCDC43* | 0.05 | 0.06 | 0.04 | 3 | D |  |
| *CCDC97* | 0.02 | 0.03 | 0.03 | 3 | D |  |
| *CCL25* | 0.06 | 0.21 | 0.12 | 3.4 | D |  |
| *CCNB1* | -0.09 | -0.08 | -0.03 | 3.6 | D |  |
| *CCNT1* | -0.03 | -0.03 | -0.03 | 4.5 | D |  |
| *CD72* | -0.02 | -0.03 | -0.03 | 3.3 | D |  |
| *CDC20* | -0.05 | -0.03 | -0.01 | 3.8 | D |  |
| *CDC6* | -0.03 | 0.04 | 0.04 | 3.7 | D |  |
| *CDCA3* | -0.06 | -0.04 | -0.01 | 3.3 | D |  |
| *CDCA8* | -0.08 | -0.05 | -0.03 | 4.6 | D |  |
| *CENPE* | -0.05 | -0.04 | -0.01 | 4.5 | D |  |
| *CENPF* | -0.07 | -0.09 | -0.05 | 3.3 | D |  |
| *CENPN* | -0.05 | -0.03 | -0.01 | 3.5 | D |  |
| *CEP85* | -0.05 | -0.03 | -0.01 | 4.5 | D |  |
| *CHAC1* | 0.09 | 0.09 | 0.05 | 3.3 | D |  |
| *CHST8* | -0.05 | -0.15 | -0.13 | 7 | D |  |
| *CKAP2* | -0.09 | -0.11 | -0.06 | 3.1 | D |  |
| *CKAP2L* | -0.05 | -0.01 | 0.01 | 4.7 | D |  |
| *CMBL* | 0.01 | -0.04 | -0.04 | 4.2 | D |  |
| *CNNM2* | -0.07 | -0.16 | -0.10 | 4.2 | D |  |
| *CRISPLD2* | 0.00 | 0.05 | 0.03 | 5.2 | D |  |
| *CUL7* | -0.03 | -0.09 | -0.06 | 4.3 | D |  |
| *CYP19A1* | -0.08 | -0.09 | -0.07 | 19.8 | D |  |
| *DBT* | 0.01 | -0.05 | -0.03 | 3.9 | D |  |
| *DCP1A* | -0.07 | -0.15 | -0.09 | 3.6 | D |  |
| *DNAJB1* | 0.08 | 0.13 | 0.07 | 3.1 | D |  |
| *DUSP14* | -0.07 | -0.09 | -0.06 | 3 | D |  |
| *ECT2* | -0.05 | -0.03 | 0.00 | 4.2 | D |  |
| *EDNRA* | -0.07 | -0.14 | -0.11 | 3.3 | D |  |
| *EEF1A1* | 0.02 | 0.03 | 0.02 | 3.1 | D |  |
| *EEFSEC* | -0.01 | 0.01 | 0.02 | 3.3 | D |  |
| *EFHD1* | -0.09 | -0.18 | -0.12 | 5.5 | D |  |
| *EHMT2* | 0.01 | -0.01 | -0.02 | 3.2 | D |  |
| *ELK1* | -0.05 | -0.12 | -0.09 | 4 | D |  |
| *ELMOD3* | -0.02 | -0.08 | -0.07 | 4 | D |  |
| *EMID1* | -0.01 | -0.03 | -0.03 | 4.3 | D |  |
| *ENSA* | 0.03 | 0.07 | 0.04 | 3.4 | D |  |
| *EPDR1* | -0.01 | 0.02 | 0.03 | 3.1 | D |  |
| *ERCC6L* | -0.04 | 0.04 | 0.04 | 3.6 | D |  |
| *ETNK2* | 0.01 | 0.00 | 0.01 | 3.6 | D |  |
| *FAM83D* | -0.09 | -0.09 | -0.05 | 3.4 | D |  |
| *FAT1* | -0.01 | 0.00 | 0.01 | 3.6 | D |  |
| *FHL3* | 0.14 | 0.27 | 0.16 | 4.7 | D |  |
| *FIGNL1* | -0.04 | 0.01 | 0.03 | 3 | D |  |
| *FSHR* | 0.01 | -0.02 | -0.03 | 4.4 | D |  |
| *FST* | -0.07 | -0.10 | -0.06 | 4 | D |  |
| *GALNT13* | -0.01 | 0.03 | 0.04 | 3.4 | D |  |
| *GCLC* | -0.07 | -0.16 | -0.10 | 6.9 | D |  |
| *GINS3* | -0.03 | 0.04 | 0.02 | 3.7 | D |  |
| *GMFG* | -0.01 | 0.04 | 0.04 | 3 | D |  |
| *GNA14* | -0.05 | -0.07 | -0.05 | 3 | D |  |
| *GOSR1* | -0.01 | -0.05 | -0.04 | 3.2 | D |  |
| *GPT* | -0.05 | -0.07 | -0.05 | 4.1 | D |  |
| *GPX3* | -0.03 | -0.13 | -0.09 | 5.4 | D |  |
| *GUCA1A* | 0.04 | 0.09 | 0.05 | 3.6 | D |  |
| *GYLTL1B* | 0.06 | 0.04 | 0.01 | 5.7 | D |  |
| *HEG1* | -0.01 | 0.00 | -0.01 | 3.1 | D |  |
| *HJURP* | -0.07 | -0.05 | -0.01 | 5.6 | D |  |
| *HMGCS1* | -0.09 | -0.10 | -0.05 | 6.9 | D |  |
| *HMMR* | -0.05 | -0.01 | 0.01 | 3.8 | D |  |
| *HSD17B1* | 0.03 | 0.08 | 0.04 | 4.8 | D |  |
| *HSPA1A* | 0.08 | 0.10 | 0.05 | 5.7 | D |  |
| *HYDIN* | 0.03 | -0.07 | -0.06 | 3.1 | D |  |
| *HYOU1* | 0.01 | 0.04 | 0.05 | 3.6 | D |  |
| *IHH* | 0.01 | 0.06 | 0.04 | 7.1 | D |  |
| *IL20RA* | -0.01 | -0.02 | -0.05 | 3.2 | D |  |
| *INHA* | -0.07 | -0.09 | -0.05 | 3.8 | D |  |
| *INHBA* | -0.04 | -0.07 | -0.05 | 4.2 | D |  |
| *INSIG1* | -0.12 | -0.19 | -0.11 | 3.8 | D |  |
| *IPMK* | -0.02 | -0.02 | -0.01 | 3.2 | D |  |
| *IRF2BP1* | -0.07 | -0.09 | -0.05 | 3.2 | D |  |
| *JAK3* | -0.03 | -0.09 | -0.07 | 5.1 | D |  |
| *JAKMIP1* | 0.04 | 0.12 | 0.07 | 4.6 | D |  |
| *KIF20A* | -0.09 | -0.09 | -0.05 | 3.6 | D |  |
| *KIF23* | -0.05 | -0.05 | -0.01 | 3.3 | D |  |
| *KIFC1* | -0.05 | -0.03 | -0.01 | 7.2 | D |  |
| *KLC2* | -0.04 | -0.07 | -0.08 | 4.3 | D |  |
| *KLK4* | -0.08 | -0.14 | -0.09 | 4.1 | D |  |
| *LAMC2* | 0.01 | 0.07 | 0.04 | 6.4 | D |  |
| *LDB1* | 0.02 | -0.01 | -0.01 | 4.2 | D |  |
| *LEMD2* | -0.04 | -0.05 | -0.03 | 3.5 | D |  |
| *LHPP* | -0.03 | -0.05 | -0.03 | 3.6 | D |  |
| *LPL* | 0.03 | 0.13 | 0.11 | 4.6 | D |  |
| *MAL2* | -0.03 | -0.08 | -0.05 | 4.6 | D |  |
| *MCAM* | -0.07 | -0.10 | -0.07 | 4 | D |  |
| *MCM10* | -0.02 | 0.06 | 0.05 | 3.3 | D |  |
| *MDC1* | -0.05 | -0.10 | -0.06 | 4.5 | D |  |
| *METTL21B* | -0.02 | 0.00 | 0.02 | 3.5 | D |  |
| *MEX3C* | -0.12 | -0.18 | -0.10 | 5 | D |  |
| *MOB3B* | -0.01 | -0.09 | -0.07 | 4.4 | D |  |
| *MPDZ* | 0.01 | -0.03 | -0.04 | 4 | D |  |
| *MTHFSD* | 0.04 | 0.04 | 0.04 | 5.4 | D |  |
| *MYO1D* | 0.00 | -0.02 | -0.04 | 4.3 | D |  |
| *NCAPH* | -0.05 | -0.03 | -0.01 | 3.2 | D |  |
| *NCOA6* | -0.07 | -0.15 | -0.09 | 3.2 | D |  |
| *NFE2L2* | -0.02 | -0.07 | -0.04 | 3.6 | D |  |
| *NFIA* | -0.09 | -0.15 | -0.08 | 3.3 | D |  |
| *NOS2* | -0.04 | -0.12 | -0.09 | 8.8 | D |  |
| *NOTCH4* | 0.01 | -0.04 | -0.04 | 3.4 | D |  |
| *NQO1* | -0.07 | -0.14 | -0.09 | 3.6 | D |  |
| *NR5A1* | -0.06 | -0.07 | -0.04 | 3.2 | D |  |
| *NSD1* | 0.00 | 0.01 | -0.01 | 3.1 | D |  |
| *NUSAP1* | -0.04 | 0.02 | 0.04 | 4.3 | D |  |
| *OIP5* | -0.03 | 0.03 | 0.05 | 3.2 | D |  |
| *P4HA2* | -0.05 | -0.05 | -0.03 | 3.4 | D |  |
| *PDK4* | 0.16 | 0.20 | 0.09 | 9.7 | D |  |
| *PDP2* | -0.05 | -0.09 | -0.05 | 3.9 | D |  |
| *PEX11B* | -0.02 | 0.01 | 0.03 | 3.1 | D |  |
| *PJA2* | -0.02 | -0.01 | 0.00 | 3.3 | D |  |
| *PLEK* | 0.06 | 0.04 | 0.03 | 4 | D |  |
| *PLEKHF2* | -0.03 | -0.03 | -0.01 | 3.4 | D |  |
| *POLR3D* | 0.01 | 0.01 | 0.00 | 4 | D |  |
| *PPP3CC* | 0.01 | -0.04 | -0.05 | 4.5 | D |  |
| *PRC1* | -0.10 | -0.12 | -0.08 | 3.3 | D |  |
| *PRCC* | -0.01 | -0.02 | -0.03 | 3.5 | D |  |
| *PRELP* | -0.02 | 0.02 | 0.06 | 4.5 | D |  |
| *PTGFRN* | 0.01 | 0.04 | 0.02 | 4.5 | D |  |
| *RBBP8* | 0.01 | 0.06 | 0.05 | 3.1 | D |  |
| *RBL2* | 0.03 | 0.03 | 0.02 | 3.8 | D |  |
| *RFX5* | -0.01 | -0.03 | -0.01 | 3.4 | D |  |
| *RGS3* | 0.05 | 0.08 | 0.04 | 3.3 | D |  |
| *RHBG* | 0.00 | -0.03 | -0.06 | 4 | D |  |
| *RIC3* | -0.03 | -0.10 | -0.09 | 3.8 | D |  |
| *RNFT1* | 0.04 | 0.05 | 0.01 | 3.1 | D |  |
| *RPS6KA4* | -0.01 | 0.04 | 0.03 | 3.3 | D |  |
| *RSPH9* | 0.00 | -0.05 | -0.03 | 3.7 | D |  |
| *SCD* | -0.03 | -0.02 | 0.00 | 5.6 | D |  |
| *SEPT4* | 0.08 | 0.21 | 0.11 | 3.1 | D |  |
| *SGOL1* | -0.07 | -0.04 | -0.01 | 3.8 | D |  |
| *SHCBP1* | -0.04 | 0.02 | 0.04 | 3.6 | D |  |
| *SKA3* | -0.06 | -0.02 | 0.01 | 3.9 | D |  |
| *SLC16A3* | -0.03 | 0.00 | -0.01 | 4.1 | D |  |
| *SLC25A34* | 0.02 | 0.09 | 0.06 | 3.1 | D |  |
| *SLC27A3* | -0.08 | -0.18 | -0.15 | 5.5 | D |  |
| *SLC4A2* | -0.02 | -0.01 | -0.01 | 4.5 | D |  |
| *SLC7A1* | -0.02 | -0.03 | -0.01 | 3.5 | D |  |
| *SPPL2B* | -0.02 | -0.05 | -0.03 | 3.2 | D |  |
| *SRGN* | 0.01 | 0.04 | 0.03 | 4 | D |  |
| *ST6GAL1* | 0.05 | 0.03 | 0.01 | 3.2 | D |  |
| *STRA6* | -0.02 | 0.01 | 0.00 | 3.6 | D |  |
| *SYNPO* | 0.01 | 0.02 | 0.01 | 3.6 | D |  |
| *TASP1* | 0.04 | 0.06 | 0.01 | 3.2 | D |  |
| *TBKBP1* | 0.04 | -0.01 | -0.03 | 6.2 | D |  |
| *TMEM138* | 0.03 | 0.01 | -0.01 | 4.5 | D |  |
| *TMEM216* | -0.02 | -0.06 | -0.05 | 4 | D |  |
| *TNPO1* | -0.04 | -0.05 | -0.03 | 6 | D |  |
| *TOP2A* | -0.10 | -0.11 | -0.06 | 6.3 | D |  |
| *TPX2* | -0.07 | -0.05 | -0.01 | 3.8 | D |  |
| *TRAM2* | 0.03 | 0.15 | 0.09 | 3.6 | D |  |
| *TRIB2* | -0.09 | -0.15 | -0.09 | 6.5 | D |  |
| *TRIM8* | -0.01 | -0.06 | -0.03 | 3 | D |  |
| *TRNAU1AP* | 0.03 | 0.04 | 0.01 | 3.3 | D |  |
| *TROAP* | -0.07 | -0.10 | -0.06 | 3.5 | D |  |
| *TTYH1* | 0.06 | 0.12 | 0.07 | 4.8 | D |  |
| *U2SURP* | 0.01 | 0.03 | 0.01 | 3 | D |  |
| *UBE2C* | -0.08 | -0.08 | -0.03 | 3.1 | D |  |
| *UBN2* | 0.01 | -0.03 | -0.01 | 3.3 | D |  |
| *UHRF1* | -0.03 | 0.09 | 0.08 | 3.5 | D |  |
| *USP28* | 0.01 | -0.03 | -0.03 | 3.8 | D |  |
| *VEGFA* | -0.09 | -0.12 | -0.08 | 5.8 | D |  |
| *VPS52* | 0.04 | 0.07 | 0.06 | 3.2 | D |  |
| *VWA8* | -0.03 | -0.12 | -0.09 | 4.1 | D |  |
| *WDR46* | 0.03 | 0.08 | 0.06 | 3.3 | D |  |
| *WDR76* | -0.05 | 0.00 | 0.01 | 4.4 | D |  |
| *ZBTB4* | -0.05 | -0.09 | -0.06 | 3.1 | D |  |
| *ZFHX3* | -0.12 | -0.19 | -0.09 | 6.3 | D |  |
| *ZNF274* | -0.04 | -0.03 | 0.01 | 3 | D |  |
| *A2M* | 0.09 | 0.27 | 0.20 | 8.1 | U |  |
| *ABCB1* | -0.08 | -0.10 | -0.07 | 3.8 | U |  |
| *ABLIM1* | 0.06 | 0.03 | 0.01 | 3.5 | U |  |
| *ACTA2* | 0.03 | 0.04 | 0.02 | 4.3 | U |  |
| *ACTN1* | 0.06 | 0.14 | 0.08 | 4.1 | U |  |
| *ACVR1* | -0.01 | 0.00 | 0.01 | 3.1 | U |  |
| *ADAMTS1* | 0.09 | 0.12 | 0.05 | 6.1 | U |  |
| *ADM* | 0.11 | 0.13 | 0.08 | 3.9 | U |  |
| *AGRN* | 0.06 | 0.14 | 0.11 | 4.1 | U |  |
| *AIF1L* | 0.06 | 0.05 | 0.02 | 4.4 | U |  |
| *AKAP8L* | 0.07 | 0.05 | -0.01 | 4.1 | U |  |
| *ALDH1A1* | -0.03 | -0.05 | -0.01 | 3.3 | U |  |
| *ALKBH4* | 0.00 | 0.03 | 0.05 | 3.4 | U |  |
| *AMIGO2* | 0.13 | 0.20 | 0.16 | 4.5 | U |  |
| *ANGPTL4* | 0.02 | 0.05 | 0.01 | 3.4 | U |  |
| *ANKH* | 0.02 | 0.01 | 0.00 | 3.6 | U |  |
| *ANKRD1* | 0.07 | 0.16 | 0.09 | 48.9 | U |  |
| *ANKRD10* | 0.01 | -0.03 | -0.06 | 3 | U |  |
| *ANKRD11* | 0.05 | 0.06 | 0.02 | 3.2 | U |  |
| *ANTXR2* | 0.09 | 0.06 | 0.02 | 3.7 | U |  |
| *ANXA1* | 0.05 | 0.15 | 0.10 | 10.9 | U |  |
| *ANXA2* | 0.01 | 0.05 | 0.04 | 4.7 | U |  |
| *AP1S2* | -0.05 | -0.09 | -0.05 | 3.1 | U |  |
| *APOD* | 0.11 | 0.12 | 0.04 | 37.5 | U |  |
| *ARF5* | 0.01 | 0.01 | 0.04 | 3.1 | U |  |
| *ARF5* | 0.01 | 0.01 | 0.04 | 3.1 | U |  |
| *ARF5* | 0.01 | 0.01 | 0.04 | 3.1 | U |  |
| *ARF5* | 0.01 | 0.01 | 0.04 | 3.1 | U |  |
| *ARHGAP32* | 0.06 | 0.08 | 0.04 | 3.5 | U |  |
| *ARHGEF11* | 0.03 | 0.04 | 0.01 | 9.9 | U |  |
| *ARHGEF2* | 0.08 | 0.20 | 0.13 | 4 | U |  |
| *ARHGEF3* | -0.05 | -0.03 | -0.02 | 7.1 | U |  |
| *ARMCX3* | 0.02 | 0.04 | 0.04 | 3.2 | U |  |
| *ARPC1B* | 0.00 | 0.06 | 0.06 | 5.5 | U |  |
| *ASCC2* | 0.01 | 0.00 | 0.00 | 3.5 | U |  |
| *ATF3* | 0.05 | 0.04 | 0.01 | 9.7 | U |  |
| *ATG14* | 0.02 | -0.02 | -0.02 | 3.1 | U |  |
| *ATRX* | 0.05 | 0.04 | 0.02 | 8.4 | U |  |
| *AUTS2* | 0.12 | 0.21 | 0.12 | 3.4 | U |  |
| *B4GALT5* | 0.00 | 0.00 | 0.01 | 3.4 | U |  |
| *B9D2* | 0.01 | 0.05 | 0.04 | 3 | U |  |
| *BAMBI* | 0.09 | 0.20 | 0.12 | 7.6 | U |  |
| *BAZ1A* | 0.12 | 0.22 | 0.11 | 3 | U |  |
| *BCAR1* | -0.06 | -0.09 | -0.05 | 3.1 | U |  |
| *BCL6* | 0.05 | 0.05 | 0.01 | 8.6 | U |  |
| *BOD1L1* | 0.08 | 0.07 | 0.02 | 3.1 | U |  |
| *BTBD3* | 0.09 | 0.09 | 0.04 | 6.1 | U |  |
| *C10orf10* | 0.08 | 0.06 | 0.02 | 7 | U |  |
| *C1orf35* | 0.01 | 0.02 | 0.01 | 4.2 | U |  |
| *C1QTNF6* | 0.04 | 0.06 | 0.04 | 4.1 | U |  |
| *C5orf30* | 0.05 | 0.14 | 0.09 | 3.1 | U |  |
| *C8orf4* | 0.06 | 0.07 | 0.04 | 37.2 | U |  |
| *CACNB3* | 0.10 | 0.09 | 0.04 | 4 | U |  |
| *CADM1* | 0.09 | 0.15 | 0.09 | 3.3 | U |  |
| *CALD1* | 0.06 | 0.09 | 0.04 | 11.6 | U |  |
| *CAPG* | 0.01 | 0.07 | 0.06 | 11.1 | U |  |
| *CAST* | 0.10 | 0.16 | 0.08 | 6.7 | U |  |
| *CBLB* | 0.08 | 0.14 | 0.09 | 3.6 | U |  |
| *CCDC80* | -0.01 | 0.04 | 0.04 | 3 | U |  |
| *CD302* | -0.01 | 0.04 | 0.03 | 3.7 | U |  |
| *CD55* | 0.09 | 0.17 | 0.07 | 6.2 | U |  |
| *CD9* | 0.09 | 0.20 | 0.10 | 7.1 | U |  |
| *CD99* | 0.04 | 0.11 | 0.08 | 6 | U |  |
| *CDC37L1* | -0.02 | -0.07 | -0.05 | 5.9 | U |  |
| *CDH1* | 0.13 | 0.32 | 0.20 | 4.3 | U |  |
| *CFLAR* | 0.09 | 0.12 | 0.09 | 6 | U |  |
| *CHMP5* | 0.01 | -0.01 | -0.01 | 3.7 | U |  |
| *CHST2* | 0.02 | -0.02 | -0.01 | 4 | U |  |
| *CIR1* | 0.04 | 0.03 | -0.01 | 3.2 | U |  |
| *CITED2* | 0.10 | 0.12 | 0.09 | 5.6 | U |  |
| *CLDN1* | 0.06 | 0.16 | 0.13 | 10.2 | U |  |
| *CLIC4* | 0.01 | -0.01 | -0.01 | 4.5 | U |  |
| *CMTM3* | 0.04 | 0.10 | 0.08 | 3.5 | U |  |
| *CNN1* | 0.08 | 0.22 | 0.13 | 4.4 | U |  |
| *COL12A1* | 0.00 | 0.01 | 0.01 | 3.4 | U |  |
| *COL18A1* | 0.04 | 0.04 | 0.02 | 4 | U |  |
| *COL1A2* | 0.00 | 0.01 | 0.01 | 5.3 | U |  |
| *COL3A1* | 0.00 | 0.02 | 0.02 | 5.5 | U |  |
| *COL4A1* | -0.06 | -0.08 | -0.07 | 4.5 | U |  |
| *COL4A5* | 0.06 | 0.07 | 0.04 | 3.5 | U |  |
| *COL5A2* | 0.01 | 0.04 | 0.03 | 8.3 | U |  |
| *COL6A1* | 0.01 | 0.01 | 0.01 | 3.2 | U |  |
| *COMMD4* | 0.01 | 0.01 | 0.01 | 3.4 | U |  |
| *CORO1A* | 0.02 | 0.13 | 0.11 | 3.2 | U |  |
| *CPEB2* | 0.01 | 0.07 | 0.04 | 3.6 | U |  |
| *CPEB4* | -0.01 | -0.01 | 0.01 | 3.2 | U |  |
| *CPT1A* | 0.03 | 0.13 | 0.09 | 4.5 | U |  |
| *CRABP2* | 0.10 | 0.18 | 0.10 | 3.7 | U |  |
| *CRYAB* | 0.02 | 0.04 | 0.02 | 5.1 | U |  |
| *CSDC2* | -0.03 | -0.03 | -0.02 | 3.1 | U |  |
| *CSNK1G2* | 0.01 | -0.01 | -0.03 | 4.2 | U |  |
| *CSNK2A1* | -0.01 | -0.02 | -0.01 | 3.7 | U |  |
| *CSRNP1* | 0.02 | 0.01 | 0.01 | 3.3 | U |  |
| *CTGF* | 0.06 | 0.06 | 0.03 | 23.4 | U |  |
| *CTR9* | 0.01 | 0.01 | 0.01 | 5.5 | U |  |
| *CTSB* | -0.02 | 0.02 | 0.04 | 3.2 | U |  |
| *CTSH* | -0.01 | 0.03 | 0.04 | 4.7 | U |  |
| *CTSK* | 0.01 | 0.09 | 0.08 | 3.2 | U |  |
| *CTSS* | -0.01 | 0.06 | 0.06 | 4.4 | U |  |
| *CTSZ* | 0.00 | 0.06 | 0.08 | 3.5 | U |  |
| *CTTNBP2NL* | -0.05 | -0.08 | -0.06 | 3.7 | U |  |
| *CUL9* | 0.03 | 0.03 | 0.01 | 4 | U |  |
| *CWC22* | -0.02 | -0.06 | -0.05 | 3.2 | U |  |
| *CYLD* | 0.03 | 0.03 | 0.01 | 3.7 | U |  |
| *CYR61* | 0.07 | 0.06 | 0.04 | 14.1 | U |  |
| *CYTH3* | 0.00 | 0.06 | 0.05 | 3.4 | U |  |
| *DAB2* | 0.04 | 0.16 | 0.13 | 4.3 | U |  |
| *DBNDD2* | 0.07 | 0.14 | 0.09 | 9.2 | U |  |
| *DCLK1* | 0.05 | 0.13 | 0.09 | 19.5 | U |  |
| *DCN* | 0.01 | 0.00 | -0.02 | 11.1 | U |  |
| *DDAH1* | 0.09 | 0.11 | 0.07 | 3.1 | U |  |
| *DDAH2* | 0.08 | 0.08 | 0.03 | 5.5 | U |  |
| *DDX42* | 0.01 | -0.01 | -0.03 | 3.6 | U |  |
| *DDX5* | 0.04 | 0.04 | 0.04 | 3.7 | U |  |
| *DENND3* | 0.05 | 0.18 | 0.14 | 4.1 | U |  |
| *DKK3* | 0.05 | 0.16 | 0.11 | 17.4 | U |  |
| *DOCK5* | -0.05 | -0.12 | -0.10 | 3.1 | U |  |
| *DPH3* | 0.08 | 0.10 | 0.06 | 3.7 | U |  |
| *DPH5* | 0.05 | 0.04 | 0.02 | 3 | U |  |
| *DSTN* | -0.01 | -0.03 | -0.04 | 4 | U |  |
| *DUSP1* | 0.09 | 0.10 | 0.06 | 4.3 | U |  |
| *DUSP7* | -0.16 | -0.21 | -0.12 | 3.6 | U |  |
| *DYNLT3* | 0.06 | 0.12 | 0.08 | 3.1 | U |  |
| *E4F1* | 0.02 | 0.04 | 0.02 | 3.9 | U |  |
| *EBNA1BP2* | 0.02 | 0.04 | 0.03 | 3.1 | U |  |
| *EFHD2* | 0.01 | 0.09 | 0.08 | 5.8 | U |  |
| *EFNA5* | -0.12 | -0.18 | -0.11 | 3.3 | U |  |
| *EGLN1* | 0.05 | 0.06 | 0.06 | 3.5 | U |  |
| *EGR1* | 0.02 | -0.03 | -0.01 | 11.3 | U |  |
| *EIF2AK4* | 0.01 | 0.01 | -0.01 | 3.5 | U |  |
| *EIF2AK4* | 0.01 | 0.01 | -0.01 | 3.5 | U |  |
| *EIF2AK4* | 0.01 | 0.01 | -0.01 | 3.2 | U |  |
| *EIF2AK4* | 0.01 | 0.01 | -0.01 | 3.2 | U |  |
| *EIF4G3* | -0.04 | -0.07 | -0.07 | 3.2 | U |  |
| *ELMO1* | 0.06 | 0.10 | 0.06 | 3.1 | U |  |
| *ELMO2* | 0.02 | 0.04 | 0.01 | 3.1 | U |  |
| *EML1* | 0.08 | 0.14 | 0.06 | 4.7 | U |  |
| *ENPP5* | 0.14 | 0.22 | 0.14 | 4.9 | U |  |
| *EPAS1* | 0.14 | 0.26 | 0.15 | 4.6 | U |  |
| *ERBB2* | 0.06 | 0.06 | 0.02 | 3.2 | U |  |
| *ERRFI1* | 0.07 | 0.10 | 0.04 | 6.1 | U |  |
| *ERRFI1* | 0.07 | 0.10 | 0.04 | 6.1 | U |  |
| *ERRFI1* | 0.07 | 0.10 | 0.04 | 4.7 | U |  |
| *ERRFI1* | 0.07 | 0.10 | 0.04 | 4.7 | U |  |
| *FABP5* | 0.01 | 0.07 | 0.06 | 8.9 | U |  |
| *FAIM* | 0.07 | 0.06 | 0.02 | 3.8 | U |  |
| *FAM126B* | 0.05 | 0.07 | 0.04 | 4.4 | U |  |
| *FAM129B* | -0.01 | 0.02 | 0.01 | 3.3 | U |  |
| *FAM160A2* | -0.02 | -0.07 | -0.06 | 3.1 | U |  |
| *FBLIM1* | 0.04 | 0.06 | 0.03 | 3 | U |  |
| *FBN1* | -0.01 | 0.05 | 0.04 | 3.8 | U |  |
| *FBXO32* | 0.04 | 0.01 | 0.00 | 3.3 | U |  |
| *FBXO33* | -0.04 | -0.03 | 0.01 | 6.2 | U |  |
| *FERMT2* | -0.01 | -0.01 | -0.01 | 5.4 | U |  |
| *FHL2* | 0.06 | 0.04 | 0.01 | 3.7 | U |  |
| *FHOD3* | 0.09 | 0.22 | 0.11 | 3.4 | U |  |
| *FLNB* | 0.07 | 0.16 | 0.07 | 6.6 | U |  |
| *FLRT2* | 0.03 | 0.10 | 0.07 | 3.3 | U |  |
| *FN1* | 0.00 | 0.01 | 0.01 | 3.9 | U |  |
| *FNBP1L* | 0.13 | 0.19 | 0.11 | 4.1 | U |  |
| *FOS* | 0.04 | 0.01 | 0.01 | 3.7 | U |  |
| *FOSL2* | 0.10 | 0.10 | 0.08 | 11.7 | U |  |
| *FOXO1* | -0.02 | -0.10 | -0.07 | 3.6 | U |  |
| *FOXP1* | 0.01 | -0.04 | -0.03 | 4.5 | U |  |
| *FSCN1* | 0.10 | 0.19 | 0.11 | 4.2 | U |  |
| *FYN* | 0.06 | 0.11 | 0.06 | 3.2 | U |  |
| *GABARAPL1* | 0.05 | 0.01 | -0.01 | 6.2 | U |  |
| *GDPD1* | 0.06 | 0.04 | 0.00 | 3.6 | U |  |
| *GEM* | 0.06 | 0.08 | 0.04 | 4.4 | U |  |
| *GEMIN8* | 0.01 | -0.05 | -0.05 | 3.1 | U |  |
| *GFPT2* | 0.08 | 0.21 | 0.14 | 7.8 | U |  |
| *GIMAP8* | 0.05 | 0.17 | 0.13 | 7.5 | U |  |
| *GIT2* | 0.04 | 0.11 | 0.06 | 4.4 | U |  |
| *GLIPR2* | 0.12 | 0.26 | 0.16 | 5.6 | U |  |
| *GLTSCR2* | 0.03 | 0.02 | 0.00 | 3.7 | U |  |
| *GNG2* | 0.04 | 0.10 | 0.06 | 7.3 | U |  |
| *GOLPH3L* | 0.02 | -0.03 | -0.03 | 4.8 | U |  |
| *GPN3* | 0.00 | 0.04 | 0.05 | 3.2 | U |  |
| *GPR155* | 0.15 | 0.27 | 0.19 | 3.3 | U |  |
| *GPRC5B* | 0.14 | 0.39 | 0.24 | 3.3 | U |  |
| *GPS2* | -0.01 | 0.00 | 0.01 | 3.1 | U |  |
| *GSN* | 0.03 | 0.07 | 0.04 | 5.3 | U |  |
| *GTF2E2* | 0.01 | 0.05 | 0.04 | 3.3 | U |  |
| *GTF2F2* | 0.05 | 0.09 | 0.06 | 4.4 | U |  |
| *GTF2H4* | -0.01 | -0.03 | -0.01 | 3.3 | U |  |
| *HDAC5* | 0.02 | -0.01 | -0.01 | 3.5 | U |  |
| *HDAC7* | 0.13 | 0.16 | 0.09 | 5.8 | U |  |
| *HECA* | 0.01 | -0.02 | -0.03 | 3.8 | U |  |
| *HMBOX1* | -0.04 | -0.09 | -0.06 | 4 | U |  |
| *HNRNPH3* | 0.01 | -0.01 | -0.01 | 3.2 | U |  |
| *HSP90AA1* | 0.04 | 0.15 | 0.13 | 4 | U |  |
| *ID1* | -0.03 | -0.09 | -0.07 | 7.4 | U |  |
| *ID3* | -0.02 | -0.09 | -0.06 | 5.8 | U |  |
| *IFI30* | -0.07 | -0.06 | -0.02 | 4.5 | U |  |
| *IFITM1* | 0.09 | 0.27 | 0.14 | 4.3 | U |  |
| *IFITM3* | 0.16 | 0.32 | 0.16 | 3.3 | U |  |
| *IGF2* | 0.05 | 0.13 | 0.12 | 5 | U |  |
| *IGF2R* | 0.06 | 0.12 | 0.09 | 6.2 | U |  |
| *IGFBP5* | 0.07 | 0.15 | 0.09 | 4.3 | U |  |
| *IGFBP6* | -0.02 | -0.05 | -0.03 | 5.1 | U |  |
| *IL10RA* | 0.05 | 0.06 | 0.05 | 3.6 | U |  |
| *IL17RA* | -0.01 | -0.03 | -0.03 | 3.5 | U |  |
| *IL18* | 0.04 | 0.08 | 0.06 | 8.9 | U |  |
| *ILF3* | 0.00 | 0.00 | 0.00 | 3 | U |  |
| *ING4* | 0.05 | 0.02 | -0.01 | 4.4 | U |  |
| *INSIG2* | 0.03 | 0.01 | 0.01 | 5.4 | U |  |
| *INTS7* | 0.05 | 0.20 | 0.13 | 3.3 | U |  |
| *IP6K2* | 0.09 | 0.08 | 0.04 | 3 | U |  |
| *IRAK1BP1* | -0.01 | -0.03 | -0.03 | 4.4 | U |  |
| *IRGQ* | 0.04 | 0.04 | 0.04 | 3.4 | U |  |
| *JAG1* | 0.11 | 0.29 | 0.16 | 3.6 | U |  |
| *JAK1* | 0.04 | 0.06 | 0.05 | 5.7 | U |  |
| *JAM3* | 0.07 | 0.10 | 0.05 | 3.2 | U |  |
| *JARID2* | 0.03 | 0.10 | 0.08 | 4.9 | U |  |
| *JUN* | 0.05 | 0.03 | 0.02 | 6.4 | U |  |
| *JUNB* | 0.08 | 0.04 | 0.01 | 3.9 | U |  |
| *KANK2* | 0.03 | 0.05 | 0.02 | 5.1 | U |  |
| *KCNJ2* | 0.09 | 0.27 | 0.17 | 3.7 | U |  |
| *KCTD10* | 0.04 | 0.07 | 0.07 | 3.8 | U |  |
| *KHDRBS1* | 0.00 | -0.03 | -0.03 | 3 | U |  |
| *KIAA0232* | 0.00 | -0.01 | 0.01 | 4.4 | U |  |
| *KIAA0922* | 0.02 | -0.01 | 0.00 | 3.2 | U |  |
| *KLF6* | 0.07 | 0.08 | 0.06 | 6.5 | U |  |
| *KLHL24* | 0.04 | 0.00 | -0.01 | 3.9 | U |  |
| *KLHL28* | -0.03 | -0.01 | 0.02 | 4.9 | U |  |
| *KLHL36* | -0.02 | -0.07 | -0.06 | 3.2 | U |  |
| *KRT18* | 0.09 | 0.23 | 0.13 | 62.4 | U |  |
| *KRT8* | 0.09 | 0.21 | 0.14 | 53.2 | U |  |
| *LAMB1* | 0.09 | 0.13 | 0.09 | 12 | U |  |
| *LARP7* | 0.06 | 0.12 | 0.06 | 3.3 | U |  |
| *LEF1* | 0.08 | 0.07 | 0.06 | 3.4 | U |  |
| *LEO1* | 0.03 | 0.08 | 0.06 | 3.3 | U |  |
| *LGALS3* | 0.02 | 0.07 | 0.05 | 22.9 | U |  |
| *LHFPL2* | -0.01 | 0.06 | 0.06 | 3.2 | U |  |
| *LIMCH1* | 0.10 | 0.26 | 0.19 | 3.9 | U |  |
| *LITAF* | 0.08 | 0.12 | 0.08 | 5.6 | U |  |
| *LMNA* | -0.01 | -0.01 | -0.02 | 3.5 | U |  |
| *LMTK2* | 0.02 | 0.09 | 0.09 | 8.1 | U |  |
| *LRCH1* | 0.01 | 0.06 | 0.06 | 3.8 | U |  |
| *LSG1* | 0.01 | 0.02 | 0.02 | 3.6 | U |  |
| *LUC7L3* | 0.06 | 0.09 | 0.04 | 8.9 | U |  |
| *LUM* | 0.04 | 0.07 | 0.03 | 6.1 | U |  |
| *MAGI1* | 0.09 | 0.10 | 0.06 | 6 | U |  |
| *MAGI3* | 0.02 | 0.04 | 0.01 | 4.6 | U |  |
| *MAP1B* | 0.09 | 0.18 | 0.15 | 4 | U |  |
| *MAP1LC3C* | 0.04 | 0.10 | 0.09 | 16.7 | U |  |
| *MAP2K3* | 0.04 | 0.11 | 0.08 | 3.1 | U |  |
| *MAP7D1* | 0.07 | 0.21 | 0.14 | 3.9 | U |  |
| *MAP9* | 0.04 | 0.08 | 0.06 | 3.2 | U |  |
| *MARCKS* | 0.08 | 0.14 | 0.10 | 3.7 | U |  |
| *MARCKSL1* | 0.09 | 0.09 | 0.05 | 3.2 | U |  |
| *MDK* | 0.12 | 0.14 | 0.06 | 9.9 | U |  |
| *MERTK* | 0.06 | 0.13 | 0.08 | 4.1 | U |  |
| *MESDC1* | -0.03 | -0.06 | -0.01 | 3.2 | U |  |
| *METRNL* | 0.07 | 0.24 | 0.17 | 3.1 | U |  |
| *MGST2* | -0.03 | -0.11 | -0.08 | 3.4 | U |  |
| *MIA3* | -0.09 | -0.18 | -0.13 | 4.6 | U |  |
| *MICAL1* | 0.06 | 0.28 | 0.18 | 8.9 | U |  |
| *MICAL2* | 0.04 | 0.13 | 0.09 | 3.1 | U |  |
| *MLLT11* | -0.01 | 0.09 | 0.06 | 3.3 | U |  |
| *MORF4L2* | -0.02 | 0.01 | 0.01 | 3.3 | U |  |
| *MPHOSPH8* | 0.03 | 0.09 | 0.07 | 3.4 | U |  |
| *MRPS28* | -0.05 | -0.04 | -0.01 | 3.6 | U |  |
| *MTA3* | -0.01 | -0.03 | -0.01 | 3.9 | U |  |
| *MTMR3* | -0.03 | -0.08 | -0.07 | 3.8 | U |  |
| *MTPN* | -0.05 | -0.07 | -0.03 | 4.6 | U |  |
| *MVP* | 0.03 | 0.04 | 0.01 | 3.7 | U |  |
| *MXI1* | 0.06 | 0.06 | 0.04 | 4.9 | U |  |
| *MXRA8* | 0.06 | 0.05 | 0.02 | 5.8 | U |  |
| *MYCBP2* | 0.01 | 0.00 | 0.01 | 4.6 | U |  |
| *NAGK* | 0.01 | 0.00 | -0.01 | 3.4 | U |  |
| *NDP* | 0.11 | 0.10 | 0.05 | 7.6 | U |  |
| *NDRG1* | 0.07 | 0.19 | 0.10 | 9 | U |  |
| *NDRG4* | -0.05 | -0.09 | -0.07 | 5.6 | U |  |
| *NFIL3* | 0.04 | 0.04 | 0.01 | 5 | U |  |
| *NFKBIA* | 0.03 | 0.13 | 0.11 | 3.2 | U |  |
| *NID2* | 0.05 | 0.16 | 0.10 | 18.5 | U |  |
| *NMI* | -0.10 | -0.14 | -0.08 | 5.7 | U |  |
| *NNAT* | 0.05 | 0.05 | 0.02 | 7.8 | U |  |
| *NPC2* | 0.04 | 0.09 | 0.06 | 3 | U |  |
| *NR2F1* | 0.06 | 0.05 | 0.01 | 4.1 | U |  |
| *NR4A2* | 0.06 | 0.05 | 0.03 | 3.5 | U |  |
| *NTN4* | 0.09 | 0.11 | 0.06 | 7.3 | U |  |
| *OCIAD2* | 0.03 | 0.11 | 0.10 | 3.5 | U |  |
| *ODF2L* | 0.24 | 0.33 | 0.16 | 5.8 | U |  |
| *OLR1* | 0.01 | 0.09 | 0.07 | 11.7 | U |  |
| *OSTF1* | 0.04 | 0.14 | 0.09 | 3.2 | U |  |
| *PCDH7* | 0.13 | 0.18 | 0.07 | 7 | U |  |
| *PCOLCE2* | 0.09 | 0.16 | 0.09 | 4.6 | U |  |
| *PDCD10* | 0.01 | 0.07 | 0.07 | 3.1 | U |  |
| *PDCD4* | 0.06 | 0.08 | 0.05 | 3.9 | U |  |
| *PDK4* | 0.16 | 0.20 | 0.09 | 9.7 | U |  |
| *PDLIM1* | 0.01 | 0.01 | 0.01 | 3.9 | U |  |
| *PDLIM4* | -0.07 | -0.14 | -0.09 | 4.6 | U |  |
| *PELI1* | 0.06 | 0.06 | 0.04 | 4.7 | U |  |
| *PHF3* | 0.03 | 0.02 | 0.00 | 6.4 | U |  |
| *PHLDA1* | 0.13 | 0.20 | 0.11 | 4.7 | U |  |
| *PIGT* | 0.05 | 0.09 | 0.06 | 4.3 | U |  |
| *PIK3CA* | 0.05 | 0.05 | 0.03 | 4.3 | U |  |
| *PIM1* | 0.07 | 0.11 | 0.08 | 4 | U |  |
| *PKIB* | 0.00 | 0.11 | 0.10 | 6.5 | U |  |
| *PKIG* | 0.06 | 0.09 | 0.03 | 3.6 | U |  |
| *PLAT* | 0.00 | 0.02 | 0.02 | 26.5 | U |  |
| *PLAUR* | 0.03 | 0.13 | 0.09 | 6.7 | U |  |
| *PLCL2* | 0.08 | 0.06 | 0.03 | 3 | U |  |
| *PLEKHB2* | -0.01 | 0.04 | 0.06 | 3.6 | U |  |
| *PLEKHG2* | 0.06 | 0.07 | 0.05 | 3.3 | U |  |
| *PLEKHO1* | 0.09 | 0.09 | 0.04 | 4.8 | U |  |
| *PLIN2* | 0.04 | 0.12 | 0.07 | 6.6 | U |  |
| *PLSCR4* | 0.04 | 0.02 | 0.02 | 5.4 | U |  |
| *PLXDC2* | 0.08 | 0.14 | 0.09 | 9.1 | U |  |
| *PLXNB2* | 0.00 | 0.01 | 0.01 | 3.8 | U |  |
| *POSTN* | 0.02 | 0.05 | 0.01 | 9.9 | U |  |
| *PPFIBP1* | 0.07 | 0.13 | 0.08 | 3.3 | U |  |
| *PPIE* | 0.04 | 0.06 | 0.03 | 3.1 | U |  |
| *PRICKLE1* | 0.14 | 0.16 | 0.09 | 8.2 | U |  |
| *PRPF38B* | 0.02 | 0.02 | 0.01 | 7.1 | U |  |
| *PRRC2C* | 0.02 | 0.02 | 0.01 | 3.2 | U |  |
| *PRSS23* | -0.11 | -0.19 | -0.12 | 10.9 | U |  |
| *PSMB1* | 0.00 | 0.02 | 0.01 | 3.5 | U |  |
| *PTPN21* | 0.11 | 0.20 | 0.12 | 3.2 | U |  |
| *PTPRK* | 0.06 | 0.04 | 0.01 | 3.6 | U |  |
| *PTX3* | 0.06 | 0.15 | 0.10 | 24.1 | U |  |
| *PVRL2* | 0.01 | -0.04 | -0.05 | 3.8 | U |  |
| *PYGL* | 0.01 | 0.07 | 0.05 | 4.9 | U |  |
| *QSOX1* | -0.01 | -0.03 | -0.01 | 5.9 | U |  |
| *RAB22A* | -0.02 | -0.03 | -0.02 | 3.3 | U |  |
| *RAB31* | 0.06 | 0.11 | 0.06 | 3.2 | U |  |
| *RAB7A* | 0.00 | 0.01 | 0.02 | 3.1 | U |  |
| *RABEP1* | -0.02 | -0.07 | -0.03 | 3.9 | U |  |
| *RAD52* | -0.05 | -0.18 | -0.14 | 3.5 | U |  |
| *RALA* | -0.03 | -0.06 | -0.05 | 3.9 | U |  |
| *RAP2C* | 0.06 | 0.16 | 0.11 | 4 | U |  |
| *RASA2* | 0.06 | 0.15 | 0.10 | 4.1 | U |  |
| *RASAL2* | 0.07 | 0.18 | 0.11 | 4.2 | U |  |
| *RBFOX2* | 0.01 | -0.01 | -0.01 | 6.5 | U |  |
| *RBKS* | 0.04 | 0.08 | 0.06 | 3.1 | U |  |
| *RBM17* | -0.01 | -0.01 | 0.00 | 4.5 | U |  |
| *RBM24* | 0.11 | 0.06 | 0.03 | 4.6 | U |  |
| *RBM25* | 0.01 | 0.04 | 0.01 | 3.9 | U |  |
| *RBMS2* | 0.01 | -0.06 | -0.05 | 6.3 | U |  |
| *RBP1* | 0.08 | 0.12 | 0.03 | 3.4 | U |  |
| *RCN2* | 0.08 | 0.09 | 0.05 | 4.4 | U |  |
| *RELN* | 0.04 | 0.01 | 0.01 | 7.7 | U |  |
| *REXO2* | 0.09 | 0.22 | 0.13 | 4 | U |  |
| *RILPL2* | 0.16 | 0.30 | 0.16 | 4 | U |  |
| *RIOK2* | 0.00 | -0.02 | -0.02 | 3.2 | U |  |
| *RND3* | 0.14 | 0.24 | 0.13 | 35.7 | U |  |
| *RNF20* | 0.02 | 0.00 | -0.01 | 3.2 | U |  |
| *RNF8* | 0.03 | 0.00 | -0.01 | 3.1 | U |  |
| *ROCK1* | 0.03 | 0.08 | 0.05 | 3.3 | U |  |
| *ROCK2* | 0.03 | 0.07 | 0.04 | 3.8 | U |  |
| *RPIA* | 0.07 | 0.13 | 0.05 | 4.7 | U |  |
| *RSRC2* | 0.06 | 0.05 | 0.01 | 7.1 | U |  |
| *S100A11* | 0.04 | 0.13 | 0.09 | 3.3 | U |  |
| *SAFB2* | 0.04 | 0.06 | 0.04 | 3.5 | U |  |
| *SCAND1* | 0.08 | 0.06 | 0.03 | 3.5 | U |  |
| *SCAP* | 0.03 | -0.01 | -0.01 | 3.4 | U |  |
| *SCG5* | 0.07 | 0.09 | 0.06 | 3.1 | U |  |
| *SCN5A* | 0.08 | 0.18 | 0.09 | 3.9 | U |  |
| *SDC2* | 0.06 | 0.04 | 0.05 | 9 | U |  |
| *SDC4* | 0.06 | 0.13 | 0.09 | 3.4 | U |  |
| *SDE2* | -0.01 | -0.09 | -0.07 | 6.3 | U |  |
| *SERPINB8* | 0.07 | 0.07 | 0.02 | 7.4 | U |  |
| *SERPINE1* | 0.08 | 0.22 | 0.12 | 19.3 | U |  |
| *SERTAD1* | 0.05 | 0.06 | 0.03 | 4.3 | U |  |
| *SETD7* | 0.02 | 0.01 | 0.01 | 4 | U |  |
| *SH3KBP1* | 0.09 | 0.21 | 0.13 | 7.4 | U |  |
| *SH3RF1* | 0.04 | 0.08 | 0.06 | 4 | U |  |
| *SHF* | 0.09 | 0.09 | 0.04 | 3.9 | U |  |
| *SHISA2* | -0.01 | -0.05 | -0.04 | 3.9 | U |  |
| *SLC17A5* | 0.05 | 0.10 | 0.06 | 9 | U |  |
| *SLC25A17* | 0.09 | 0.15 | 0.09 | 3.4 | U |  |
| *SLC25A29* | 0.10 | 0.07 | 0.01 | 4.2 | U |  |
| *SLC39A8* | -0.07 | -0.11 | -0.07 | 5.2 | U |  |
| *SLC40A1* | 0.01 | 0.02 | 0.03 | 7.2 | U |  |
| *SLITRK2* | 0.08 | 0.09 | 0.07 | 4.3 | U |  |
| *SMAD3* | 0.09 | 0.08 | 0.06 | 4.3 | U |  |
| *SMS* | 0.11 | 0.20 | 0.14 | 3.8 | U |  |
| *SNN* | 0.04 | 0.01 | 0.01 | 3.2 | U |  |
| *SNRNP200* | 0.01 | -0.01 | 0.00 | 3.2 | U |  |
| *SNTB2* | -0.01 | -0.09 | -0.05 | 4 | U |  |
| *SNX9* | -0.05 | -0.08 | -0.06 | 3 | U |  |
| *SPARC* | 0.10 | 0.21 | 0.12 | 3.6 | U |  |
| *SPOCK2* | 0.04 | 0.04 | 0.01 | 23.8 | U |  |
| *SPP1* | 0.01 | 0.08 | 0.06 | 14.1 | U |  |
| *SRP14* | 0.10 | 0.18 | 0.11 | 4.7 | U |  |
| *STAR* | 0.01 | 0.05 | 0.05 | 22.5 | U |  |
| *STARD3NL* | 0.03 | 0.06 | 0.04 | 3.7 | U |  |
| *STAT3* | 0.08 | 0.11 | 0.06 | 4.3 | U |  |
| *STAU1* | -0.01 | -0.08 | -0.08 | 4.1 | U |  |
| *STK17A* | 0.06 | 0.16 | 0.12 | 5 | U |  |
| *STK38L* | 0.04 | 0.01 | 0.00 | 3.1 | U |  |
| *SYAP1* | 0.00 | -0.01 | 0.00 | 3.1 | U |  |
| *SYNCRIP* | 0.02 | 0.09 | 0.06 | 3.6 | U |  |
| *SYNE1* | 0.10 | 0.15 | 0.12 | 7.6 | U |  |
| *SYT11* | -0.09 | -0.16 | -0.10 | 3.2 | U |  |
| *TACC2* | 0.09 | 0.17 | 0.11 | 4.8 | U |  |
| *TAGLN* | 0.08 | 0.21 | 0.09 | 10.8 | U |  |
| *TAGLN2* | 0.04 | 0.16 | 0.11 | 4.3 | U |  |
| *TAX1BP3* | 0.05 | 0.06 | 0.04 | 4.8 | U |  |
| *TCF3* | 0.04 | 0.04 | 0.02 | 3.3 | U |  |
| *TCF7L2* | 0.04 | -0.01 | -0.01 | 5.8 | U |  |
| *TCN2* | 0.03 | 0.09 | 0.06 | 3.4 | U |  |
| *TCP11L2* | 0.06 | 0.04 | 0.02 | 3.2 | U |  |
| *TDRD7* | -0.01 | -0.03 | -0.01 | 4.3 | U |  |
| *TES* | 0.09 | 0.17 | 0.12 | 3.7 | U |  |
| *TGFBR2* | -0.01 | 0.06 | 0.06 | 5 | U |  |
| *TGIF1* | 0.12 | 0.13 | 0.06 | 3.4 | U |  |
| *THBS2* | 0.06 | 0.17 | 0.09 | 23.9 | U |  |
| *TIMP1* | 0.07 | 0.13 | 0.09 | 68.9 | U |  |
| *TIMP2* | -0.06 | -0.10 | -0.07 | 3.3 | U |  |
| *TLN1* | 0.01 | 0.08 | 0.06 | 4.1 | U |  |
| *TMEM14A* | 0.13 | 0.19 | 0.10 | 5.9 | U |  |
| *TMEM176A* | 0.02 | 0.09 | 0.07 | 5.4 | U |  |
| *TMEM176B* | 0.04 | 0.13 | 0.08 | 3.1 | U |  |
| *TMTC2* | 0.00 | -0.06 | -0.05 | 3.9 | U |  |
| *TNFAIP6* | -0.15 | -0.20 | -0.12 | 7.1 | U |  |
| *TNFRSF12A* | 0.11 | 0.21 | 0.11 | 10.4 | U |  |
| *TNFRSF1A* | 0.06 | 0.12 | 0.08 | 3.2 | U |  |
| *TOP1* | 0.09 | 0.15 | 0.09 | 5.1 | U |  |
| *TOPORS* | 0.04 | 0.02 | 0.01 | 5.7 | U |  |
| *TP53INP1* | 0.05 | 0.03 | 0.01 | 8.9 | U |  |
| *TPGS1* | -0.03 | -0.15 | -0.10 | 3 | U |  |
| *TRANK1* | 0.09 | 0.11 | 0.06 | 3.6 | U |  |
| *TSC22D3* | 0.03 | 0.01 | 0.00 | 3.4 | U |  |
| *TSPO* | 0.04 | 0.12 | 0.08 | 5.7 | U |  |
| *TUBB6* | 0.04 | 0.13 | 0.09 | 5.1 | U |  |
| *TYRO3* | 0.07 | 0.16 | 0.10 | 3.1 | U |  |
| *UACA* | 0.02 | 0.14 | 0.08 | 3.2 | U |  |
| *USF1* | 0.10 | 0.12 | 0.06 | 6.8 | U |  |
| *VAT1* | 0.06 | 0.13 | 0.09 | 6.8 | U |  |
| *VAV2* | 0.04 | 0.09 | 0.05 | 5.6 | U |  |
| *VCL* | 0.03 | 0.04 | 0.02 | 4.3 | U |  |
| *VMP1* | 0.10 | 0.24 | 0.14 | 5.3 | U |  |
| *VNN1* | 0.08 | 0.06 | 0.04 | 15.1 | U |  |
| *WASL* | 0.04 | -0.01 | -0.03 | 4.3 | U |  |
| *WDFY4* | 0.04 | 0.12 | 0.10 | 11.1 | U |  |
| *WDR44* | 0.08 | 0.12 | 0.06 | 5 | U |  |
| *WHSC1L1* | 0.03 | 0.01 | -0.01 | 4.5 | U |  |
| *YPEL5* | -0.01 | -0.10 | -0.07 | 3.9 | U |  |
| *ZC3H8* | 0.04 | 0.10 | 0.06 | 3.2 | U |  |
| *ZDHHC23* | -0.03 | -0.07 | -0.03 | 4.7 | U |  |
| *ZFP36L1* | 0.01 | 0.11 | 0.09 | 3.9 | U |  |
| *ZFYVE1* | 0.04 | -0.01 | -0.02 | 3.3 | U |  |
| *ZMIZ2* | 0.04 | 0.06 | 0.03 | 3.2 | U |  |
| *ZNF281* | 0.03 | 0.01 | 0.01 | 3.8 | U |  |
| *ZNF292* | -0.05 | -0.10 | -0.08 | 5.7 | U |  |
| *ZNF317* | -0.01 | -0.03 | -0.03 | 4.2 | U |  |
| *ZNF398* | 0.00 | -0.02 | -0.02 | 4.2 | U |  |
| *ZNF462* | 0.04 | 0.01 | 0.01 | 7.5 | U |  |
| *ZNF521* | 0.06 | 0.09 | 0.04 | 4.6 | U |  |
| *ZNF608* | 0.13 | 0.12 | 0.06 | 4.9 | U |  |
| *ZNHIT3* | 0.06 | 0.13 | 0.09 | 3.1 | U |  |
| *ZNRF1* | 0.04 | 0.02 | -0.02 | 3.4 | U |  |

The first three columns **BL rate FC**, **Kinetic FC** and **Morphology FC** are the regression coefficient obtained in our analysis, **FC** is the FC observed in (Hatzirodos et al. 2014b) and **trend** indicates the direction of the FC, U stands for up-regulated and D for down-regulated in small atretic follicles respect to healthy follicles. The red color indicates that the expression trend in IVP scores is up-regulated as in the (Hatzirodos et al. 2014b), green color indicates that the expression in our result is down-regulated as in the (Hatzirodos et al. 2014b), yellow is used when our results showed opposite behavior from (Hatzirodos et al. 2014b).

# Table I. Comparison of the regression coefficient obtained for IVP scores with the differential expression profile obtained from comparison of small antral follicles against big antral follicles (Hatzirodos et al. 2014a).

| **Gene Name** | **BL rate (FC)** | **Kinetic (FC)** | **Morphology (FC)** | **FC (small to large follicles)** | **trend** |  |
| --- | --- | --- | --- | --- | --- | --- |
| *ABAT* | 0.00 | -0.01 | 0.00 | 3.5 | D |  |
| *ABCC8* | 0.04 | 0.17 | 0.13 | 4.7 | D |  |
| *ADCK3* | 0.04 | 0.06 | 0.04 | 3.4 | D |  |
| *SEPT4* | 0.08 | 0.21 | 0.11 | 15.3 | D |  |
| *ANGPTL2* | 0.03 | 0.06 | 0.04 | 3.1 | D |  |
| *AP3B2* | 0.05 | 0.07 | 0.03 | 5.5 | D |  |
| *APBB2* | 0.05 | 0.13 | 0.08 | 3.2 | D |  |
| *BANK1* | -0.01 | 0.09 | 0.08 | 3.1 | D |  |
| *CA14* | 0.01 | 0.04 | 0.03 | 8.8 | D |  |
| *CARTPT* | -0.01 | -0.03 | -0.03 | 14 | D |  |
| *ANGPT2* | 0.09 | 0.09 | 0.04 | 11.5 | D |  |
| *CHRDL1* | 0.06 | 0.06 | 0.02 | 3.7 | D |  |
| *CMTM8* | 0.04 | 0.08 | 0.04 | 3.4 | D |  |
| *COL1A1* | 0.01 | 0.03 | 0.03 | 3 | D |  |
| *CPEB1* | 0.04 | 0.06 | 0.05 | 3.9 | D |  |
| *DDO* | 0.02 | 0.01 | 0.01 | 3.6 | D |  |
| *EMX2* | 0.01 | -0.02 | -0.01 | 3.1 | D |  |
| *ENPP1* | -0.01 | -0.03 | -0.01 | 4.1 | D |  |
| *EPHA1* | -0.01 | 0.06 | 0.03 | 3.2 | D |  |
| *EPHX1* | 0.03 | 0.04 | 0.02 | 3.4 | D |  |
| *FEZ1* | 0.03 | 0.03 | 0.01 | 3.8 | D |  |
| *FHL2* | 0.06 | 0.04 | 0.01 | 3.3 | D |  |
| *FOS* | 0.04 | 0.01 | 0.01 | 4 | D |  |
| *FXYD6* | 0.04 | 0.07 | 0.04 | 3.2 | D |  |
| *GALNT13* | -0.01 | 0.03 | 0.04 | 5.3 | D |  |
| *CDH2* | 0.09 | 0.21 | 0.11 | 4 | D |  |
| *GUCA1A* | 0.04 | 0.09 | 0.05 | 5.1 | D |  |
| *GYLTL1B* | 0.06 | 0.04 | 0.01 | 3 | D |  |
| *GATM* | 0.10 | 0.13 | 0.09 | 11.1 | D |  |
| *HES1* | 0.09 | 0.15 | 0.05 | 3.2 | D |  |
| *HOPX* | -0.01 | 0.01 | 0.03 | 13.8 | D |  |
| *IHH* | 0.01 | 0.06 | 0.04 | 16.6 | D |  |
| *IL20RA* | -0.01 | -0.02 | -0.05 | 3.4 | D |  |
| *IL33* | -0.01 | 0.01 | -0.01 | 3.6 | D |  |
| *JAKMIP1* | 0.04 | 0.12 | 0.07 | 20.8 | D |  |
| *KIT* | 0.05 | 0.21 | 0.13 | 23.1 | D |  |
| *LAMC2* | 0.01 | 0.07 | 0.04 | 3.4 | D |  |
| *LRRC1* | 0.04 | 0.10 | 0.05 | 3.8 | D |  |
| *LRRC17* | 0.02 | 0.03 | 0.02 | 7.1 | D |  |
| *MAN1A1* | 0.05 | 0.06 | 0.04 | 4.2 | D |  |
| *MEST* | -0.01 | 0.01 | 0.01 | 28.7 | D |  |
| *MFAP2* | -0.01 | 0.00 | -0.01 | 4.6 | D |  |
| *MMD* | 0.01 | 0.01 | -0.01 | 3.1 | D |  |
| *MMP16* | 0.06 | 0.19 | 0.15 | 4 | D |  |
| *MYC* | 0.06 | 0.11 | 0.06 | 16.4 | D |  |
| *MYO10* | 0.04 | 0.03 | 0.00 | 6.8 | D |  |
| *MYO1D* | 0.00 | -0.02 | -0.04 | 3 | D |  |
| *NALCN* | 0.06 | 0.10 | 0.05 | 6 | D |  |
| *NEDD9* | 0.02 | 0.09 | 0.06 | 5.9 | D |  |
| *NPNT* | 0.06 | 0.11 | 0.06 | 3.2 | D |  |
| *NUP210* | -0.01 | 0.02 | 0.04 | 4.7 | D |  |
| *PAPSS2* | 0.05 | 0.11 | 0.06 | 7.8 | D |  |
| *HMOX1* | 0.12 | 0.13 | 0.08 | 4.1 | D |  |
| *PFKFB3* | 0.06 | 0.25 | 0.17 | 3.2 | D |  |
| *PHGDH* | 0.01 | 0.07 | 0.06 | 3.7 | D |  |
| *PRSS35* | 0.01 | 0.07 | 0.06 | 3.7 | D |  |
| *RASL11B* | 0.01 | 0.01 | 0.01 | 6.8 | D |  |
| *RENBP* | 0.05 | 0.08 | 0.05 | 3.6 | D |  |
| *PDGFC* | 0.11 | 0.26 | 0.15 | 4.6 | D |  |
| *RPRM* | 0.02 | 0.01 | 0.01 | 5.8 | D |  |
| *RYR2* | 0.02 | 0.04 | 0.03 | 7 | D |  |
| *SASH1* | 0.07 | 0.08 | 0.04 | 3.5 | D |  |
| *RGS2* | 0.16 | 0.27 | 0.16 | 3.4 | D |  |
| *SEL1L3* | 0.08 | 0.18 | 0.11 | 3.6 | D |  |
| *SHISA2* | -0.01 | -0.05 | -0.04 | 3.5 | D |  |
| *SNX31* | 0.06 | 0.09 | 0.04 | 6 | D |  |
| *STARD10* | -0.01 | -0.01 | -0.01 | 3.4 | D |  |
| *SVOPL* | 0.01 | -0.05 | -0.04 | 8.6 | D |  |
| *TGIF1* | 0.12 | 0.13 | 0.06 | 6.1 | D |  |
| *TNFAIP2* | 0.04 | 0.02 | 0.04 | 17.5 | D |  |
| *TRIM2* | 0.04 | 0.06 | 0.04 | 3.3 | D |  |
| *TTN* | 0.03 | 0.05 | 0.01 | 3.6 | D |  |
| *ABCB1* | -0.08 | -0.10 | -0.07 | 5.1 | U |  |
| *ACSS2* | -0.10 | -0.14 | -0.11 | 3.5 | U |  |
| *ABLIM1* | 0.06 | 0.03 | 0.01 | 3.7 | U |  |
| *ACBD5* | -0.05 | -0.10 | -0.07 | 3.5 | U |  |
| *ADAM12* | -0.07 | -0.14 | -0.10 | 6.6 | U |  |
| *ADAM10* | 0.02 | 0.00 | -0.01 | 3.7 | U |  |
| *ADAM9* | -0.11 | -0.16 | -0.10 | 8.4 | U |  |
| *AHCYL2* | -0.07 | -0.09 | -0.04 | 4.4 | U |  |
| *ADNP* | 0.01 | -0.01 | -0.03 | 3.7 | U |  |
| *AFF1* | -0.05 | -0.10 | -0.07 | 5.5 | U |  |
| *ARFGAP3* | -0.10 | -0.20 | -0.14 | 7.6 | U |  |
| *AKAP8L* | 0.07 | 0.05 | -0.01 | 3.6 | U |  |
| *AMIGO2* | 0.13 | 0.20 | 0.16 | 4.7 | U |  |
| *ANAPC5* | -0.04 | -0.10 | -0.09 | 3.1 | U |  |
| *ANKRD10* | 0.01 | -0.03 | -0.06 | 3.1 | U |  |
| *ANKRD12* | 0.01 | 0.00 | -0.01 | 3.4 | U |  |
| *AP1S2* | -0.05 | -0.09 | -0.05 | 3.5 | U |  |
| *AP2B1* | -0.06 | -0.14 | -0.10 | 6.8 | U |  |
| *AP3S2* | -0.01 | -0.03 | -0.03 | 3.6 | U |  |
| *APC* | -0.05 | -0.10 | -0.07 | 4 | U |  |
| *APOA1* | -0.01 | -0.03 | -0.02 | 3.2 | U |  |
| *ARHGAP18* | -0.16 | -0.24 | -0.15 | 20.5 | U |  |
| *ARHGAP17* | 0.00 | -0.01 | -0.01 | 4.8 | U |  |
| *ATP13A3* | -0.10 | -0.10 | -0.06 | 3.6 | U |  |
| *ARHGEF3* | -0.05 | -0.03 | -0.02 | 4.8 | U |  |
| *ARHGEF6* | -0.04 | -0.09 | -0.06 | 3.5 | U |  |
| *ARPC1B* | 0.00 | 0.06 | 0.06 | 3.3 | U |  |
| *B3GALT2* | -0.10 | -0.20 | -0.12 | 6.5 | U |  |
| *ATP6V1A* | 0.02 | 0.11 | 0.09 | 4.3 | U |  |
| *BEX2* | -0.18 | -0.25 | -0.18 | 9 | U |  |
| *BCAS3* | 0.01 | -0.04 | -0.04 | 6 | U |  |
| *BCL2L2* | -0.01 | -0.04 | -0.02 | 3.2 | U |  |
| *BMP2K* | -0.03 | -0.05 | -0.03 | 3.8 | U |  |
| *BMPR1A* | -0.02 | -0.03 | -0.01 | 3.1 | U |  |
| *BMPR2* | -0.09 | -0.17 | -0.11 | 3.3 | U |  |
| *BRWD1* | 0.01 | 0.01 | 0.01 | 3.3 | U |  |
| *BRWD3* | -0.01 | -0.06 | -0.05 | 3.2 | U |  |
| *BTBD7* | -0.16 | -0.24 | -0.15 | 4.3 | U |  |
| *BZW1* | 0.02 | 0.08 | 0.06 | 4.2 | U |  |
| *BZW2* | -0.04 | -0.14 | -0.10 | 3.6 | U |  |
| *CA8* | -0.03 | -0.03 | -0.01 | 3 | U |  |
| *CANX* | -0.04 | -0.01 | 0.03 | 3.1 | U |  |
| *CCM2* | -0.04 | -0.07 | -0.04 | 3.6 | U |  |
| *CDH11* | -0.04 | -0.05 | -0.02 | 4.6 | U |  |
| *CDK13* | -0.07 | -0.14 | -0.11 | 4.3 | U |  |
| *CHCHD10* | -0.15 | -0.28 | -0.19 | 3.3 | U |  |
| *CHD1* | 0.01 | -0.01 | -0.01 | 4.7 | U |  |
| *CHST10* | -0.03 | -0.04 | -0.01 | 3.1 | U |  |
| *CITED1* | -0.07 | -0.18 | -0.14 | 3.1 | U |  |
| *CITED2* | 0.10 | 0.12 | 0.09 | 3.1 | U |  |
| *CLGN* | -0.02 | -0.05 | -0.04 | 3.2 | U |  |
| *CLIC4* | 0.01 | -0.01 | -0.01 | 3.7 | U |  |
| *CLTC* | -0.04 | -0.02 | -0.01 | 3.2 | U |  |
| *CLTC* | -0.04 | -0.02 | -0.01 | 3.2 | U |  |
| *CLTC* | -0.04 | -0.02 | -0.01 | 3.2 | U |  |
| *CLTC* | -0.04 | -0.02 | -0.01 | 3.2 | U |  |
| *COL16A1* | -0.10 | -0.16 | -0.15 | 6 | U |  |
| *COL4A1* | -0.06 | -0.08 | -0.07 | 6.8 | U |  |
| *COPZ1* | -0.03 | -0.09 | -0.06 | 3.3 | U |  |
| *CPD* | -0.10 | -0.18 | -0.11 | 11.8 | U |  |
| *CPNE8* | -0.09 | -0.19 | -0.12 | 3.7 | U |  |
| *CPEB4* | -0.01 | -0.01 | 0.01 | 5.1 | U |  |
| *CYP11A1* | -0.07 | -0.13 | -0.10 | 5.8 | U |  |
| *CREB3L2* | -0.01 | -0.04 | -0.03 | 3.7 | U |  |
| *CSDE1* | -0.01 | -0.03 | -0.02 | 4.7 | U |  |
| *CSPG4* | -0.06 | -0.10 | -0.08 | 5.5 | U |  |
| *CTR9* | 0.01 | 0.01 | 0.01 | 3.9 | U |  |
| *CTSB* | -0.02 | 0.02 | 0.04 | 4.4 | U |  |
| *CUL1* | -0.02 | -0.05 | -0.03 | 3 | U |  |
| *CUL3* | -0.03 | -0.08 | -0.03 | 3.5 | U |  |
| *CYBB* | -0.06 | -0.04 | -0.01 | 18.1 | U |  |
| *CYP19A1* | -0.08 | -0.09 | -0.07 | 14.2 | U |  |
| *DACT1* | -0.13 | -0.20 | -0.12 | 8.4 | U |  |
| *DDX26B* | -0.09 | -0.19 | -0.13 | 9.7 | U |  |
| *DERL1* | -0.07 | -0.15 | -0.10 | 4.1 | U |  |
| *DDX46* | -0.01 | 0.00 | 0.00 | 3.4 | U |  |
| *DPYD* | -0.13 | -0.20 | -0.11 | 3.4 | U |  |
| *DICER1* | -0.05 | -0.05 | -0.03 | 3.1 | U |  |
| *DKK3* | 0.05 | 0.16 | 0.11 | 5.3 | U |  |
| *DPYSL2* | -0.07 | -0.07 | -0.01 | 3.8 | U |  |
| *DTNA* | -0.10 | -0.12 | -0.06 | 8.5 | U |  |
| *ECE1* | -0.10 | -0.18 | -0.12 | 9.5 | U |  |
| *EFNA5* | -0.12 | -0.18 | -0.11 | 8.6 | U |  |
| *EDEM2* | -0.07 | -0.15 | -0.13 | 3.1 | U |  |
| *EIF4EBP1* | -0.10 | -0.16 | -0.12 | 7.3 | U |  |
| *FAM114A1* | -0.08 | -0.19 | -0.14 | 18.8 | U |  |
| *EIF4G3* | -0.04 | -0.07 | -0.07 | 7.4 | U |  |
| *ERRFI1* | 0.07 | 0.10 | 0.04 | 3.6 | U |  |
| *F2R* | -0.05 | -0.05 | -0.03 | 8.9 | U |  |
| *F3* | -0.01 | -0.01 | -0.03 | 7.2 | U |  |
| *FAF2* | -0.03 | -0.04 | -0.03 | 3.2 | U |  |
| *FDFT1* | -0.09 | -0.16 | -0.10 | 4.1 | U |  |
| *FAM126B* | 0.05 | 0.07 | 0.04 | 3.6 | U |  |
| *FBXL20* | 0.04 | 0.04 | 0.01 | 3.9 | U |  |
| *GNA11* | -0.08 | -0.15 | -0.09 | 3.3 | U |  |
| *FERMT2* | -0.01 | -0.01 | -0.01 | 4.2 | U |  |
| *FICD* | -0.05 | -0.10 | -0.05 | 3.4 | U |  |
| *FKBP9* | 0.00 | -0.01 | -0.01 | 3.1 | U |  |
| *FOXP2* | -0.04 | -0.10 | -0.07 | 5.3 | U |  |
| *GABARAPL1* | 0.05 | 0.01 | -0.01 | 4.6 | U |  |
| *GADD45B* | -0.04 | -0.07 | -0.04 | 6.8 | U |  |
| *GDI1* | -0.04 | -0.09 | -0.05 | 3.4 | U |  |
| *GLG1* | -0.05 | -0.12 | -0.11 | 3.3 | U |  |
| *HMGXB3* | -0.09 | -0.10 | -0.06 | 3.1 | U |  |
| *GOLGA4* | 0.01 | 0.00 | -0.01 | 4.6 | U |  |
| *GPR173* | -0.03 | -0.10 | -0.09 | 3 | U |  |
| *GPR88* | 0.06 | 0.03 | 0.02 | 3.2 | U |  |
| *GPS2* | -0.01 | 0.00 | 0.01 | 3.1 | U |  |
| *GPX3* | -0.03 | -0.13 | -0.09 | 3.2 | U |  |
| *HECTD1* | 0.00 | -0.05 | -0.04 | 3 | U |  |
| *HELZ* | -0.01 | -0.07 | -0.04 | 3.1 | U |  |
| *HERPUD1* | -0.02 | -0.12 | -0.07 | 3.3 | U |  |
| *HIATL1* | -0.01 | 0.00 | 0.01 | 3.1 | U |  |
| *HM13* | -0.01 | -0.03 | -0.01 | 4.5 | U |  |
| *ID2* | -0.12 | -0.20 | -0.14 | 4.5 | U |  |
| *HSP90AA1* | 0.04 | 0.15 | 0.13 | 7 | U |  |
| *HUWE1* | -0.03 | -0.07 | -0.05 | 3.8 | U |  |
| *IDH3A* | -0.14 | -0.19 | -0.14 | 5.5 | U |  |
| *ID3* | -0.02 | -0.09 | -0.06 | 4.3 | U |  |
| *IFI30* | -0.07 | -0.06 | -0.02 | 39 | U |  |
| *IER3* | 0.08 | 0.12 | 0.07 | 3.3 | U |  |
| *IL4R* | -0.09 | -0.10 | -0.07 | 6.2 | U |  |
| *IGFBP4* | -0.02 | -0.05 | -0.03 | 7.3 | U |  |
| *IGFBP6* | -0.02 | -0.05 | -0.03 | 3.5 | U |  |
| *ITGB5* | -0.15 | -0.25 | -0.16 | 11.4 | U |  |
| *IL6R* | -0.06 | -0.12 | -0.07 | 5.7 | U |  |
| *INHBA* | -0.04 | -0.07 | -0.05 | 3.8 | U |  |
| *INSIG2* | 0.03 | 0.01 | 0.01 | 3.2 | U |  |
| *IREB2* | -0.02 | -0.07 | -0.04 | 3.4 | U |  |
| *KIRREL* | -0.09 | -0.16 | -0.10 | 3 | U |  |
| *JARID2* | 0.03 | 0.10 | 0.08 | 3.2 | U |  |
| *LAMA1* | -0.10 | -0.17 | -0.12 | 5.8 | U |  |
| *KLF6* | 0.07 | 0.08 | 0.06 | 3.5 | U |  |
| *KLHL28* | -0.03 | -0.01 | 0.02 | 5 | U |  |
| *LARGE* | -0.07 | -0.16 | -0.14 | 3.2 | U |  |
| *LGALS3BP* | -0.07 | -0.07 | -0.04 | 3.5 | U |  |
| *LIMA1* | -0.08 | -0.09 | -0.05 | 8.2 | U |  |
| *LHFPL2* | -0.01 | 0.06 | 0.06 | 6.8 | U |  |
| *MAOA* | -0.14 | -0.24 | -0.15 | 3.1 | U |  |
| *LINGO2* | -0.03 | -0.10 | -0.07 | 5 | U |  |
| *LRIG3* | -0.04 | -0.07 | -0.03 | 3.7 | U |  |
| *LRP8* | -0.05 | -0.09 | -0.07 | 53.6 | U |  |
| *LRRC2* | -0.05 | -0.09 | -0.05 | 3.6 | U |  |
| *LUC7L3* | 0.06 | 0.09 | 0.04 | 3.8 | U |  |
| *MAL2* | -0.03 | -0.08 | -0.05 | 3.8 | U |  |
| *MGARP* | -0.09 | -0.14 | -0.10 | 3.1 | U |  |
| *MAP2K4* | -0.05 | -0.07 | -0.03 | 4.4 | U |  |
| *MAPK6* | -0.01 | 0.04 | 0.04 | 4.8 | U |  |
| *MED24* | 0.02 | 0.02 | -0.01 | 3.1 | U |  |
| *MIA3* | -0.09 | -0.18 | -0.13 | 5.4 | U |  |
| *MPV17L2* | -0.08 | -0.16 | -0.09 | 3.1 | U |  |
| *MICU1* | 0.04 | 0.10 | 0.05 | 3.2 | U |  |
| *MLEC* | -0.02 | -0.01 | 0.01 | 3.8 | U |  |
| *MPP5* | -0.01 | -0.03 | -0.01 | 7.1 | U |  |
| *NINJ1* | -0.10 | -0.14 | -0.09 | 3.2 | U |  |
| *MTPN* | -0.05 | -0.07 | -0.03 | 5.7 | U |  |
| *MTR* | -0.01 | -0.08 | -0.05 | 3.4 | U |  |
| *MYCBP2* | 0.01 | 0.00 | 0.01 | 3.4 | U |  |
| *MYO1B* | -0.05 | -0.11 | -0.08 | 5.1 | U |  |
| *NCEH1* | 0.00 | 0.02 | 0.06 | 3.1 | U |  |
| *NCOR1* | -0.01 | -0.03 | -0.04 | 3.5 | U |  |
| *NDRG3* | -0.01 | -0.05 | -0.03 | 4.4 | U |  |
| *NDRG4* | -0.05 | -0.09 | -0.07 | 6.7 | U |  |
| *NISCH* | -0.09 | -0.15 | -0.10 | 3.3 | U |  |
| *NMI* | -0.10 | -0.14 | -0.08 | 8.4 | U |  |
| *NPR3* | -0.08 | -0.15 | -0.09 | 5.2 | U |  |
| *NOL3* | -0.04 | -0.10 | -0.09 | 6 | U |  |
| *NOSTRIN* | -0.03 | -0.11 | -0.07 | 3.2 | U |  |
| *NR5A2* | -0.10 | -0.23 | -0.16 | 5.5 | U |  |
| *OBSL1* | -0.09 | -0.15 | -0.11 | 5.8 | U |  |
| *NRP1* | 0.06 | 0.04 | 0.04 | 3 | U |  |
| *NUP85* | -0.05 | -0.06 | -0.05 | 3.2 | U |  |
| *PCOLCE* | -0.09 | -0.16 | -0.11 | 5.7 | U |  |
| *ODF2L* | 0.24 | 0.33 | 0.16 | 4.4 | U |  |
| *OPTN* | -0.04 | -0.10 | -0.09 | 6.5 | U |  |
| *OSBPL8* | -0.07 | -0.08 | -0.05 | 3.1 | U |  |
| *OXR1* | -0.04 | -0.10 | -0.07 | 3.2 | U |  |
| *PARD3B* | -0.06 | -0.12 | -0.09 | 3.2 | U |  |
| *PBRM1* | -0.05 | -0.10 | -0.07 | 4.3 | U |  |
| *PDLIM4* | -0.07 | -0.14 | -0.09 | 5 | U |  |
| *PDCD4* | 0.06 | 0.08 | 0.05 | 4 | U |  |
| *PDP1* | -0.08 | -0.10 | -0.06 | 3.2 | U |  |
| *PDSS1* | -0.09 | -0.16 | -0.12 | 6 | U |  |
| *PDPK1* | -0.01 | -0.01 | 0.01 | 4.5 | U |  |
| *PGR* | -0.10 | -0.19 | -0.11 | 4.7 | U |  |
| *PFKM* | -0.06 | -0.12 | -0.10 | 3.7 | U |  |
| *PIK3R1* | -0.10 | -0.20 | -0.12 | 9 | U |  |
| *PHF3* | 0.03 | 0.02 | 0.00 | 3.1 | U |  |
| *PIGS* | -0.04 | -0.12 | -0.10 | 5.5 | U |  |
| *PLD1* | -0.11 | -0.20 | -0.13 | 3.7 | U |  |
| *PIP4K2A* | 0.05 | 0.09 | 0.06 | 3.1 | U |  |
| *PLAT* | 0.00 | 0.02 | 0.02 | 17.5 | U |  |
| *PLEKHA2* | -0.09 | -0.13 | -0.08 | 4.2 | U |  |
| *PLEKHG1* | -0.11 | -0.16 | -0.10 | 3.6 | U |  |
| *PLEKHB2* | -0.01 | 0.04 | 0.06 | 3.5 | U |  |
| *POR* | -0.12 | -0.18 | -0.14 | 3.3 | U |  |
| *PLEKHH3* | -0.06 | -0.10 | -0.08 | 4.1 | U |  |
| *PLXNB2* | 0.00 | 0.01 | 0.01 | 4.7 | U |  |
| *PLXNC1* | -0.04 | -0.02 | -0.01 | 4.8 | U |  |
| *PNISR* | 0.10 | 0.14 | 0.05 | 3.7 | U |  |
| *PPM1K* | -0.13 | -0.14 | -0.07 | 5.9 | U |  |
| *PRSS23* | -0.11 | -0.19 | -0.12 | 48.5 | U |  |
| *PPP2R5E* | 0.00 | -0.01 | -0.01 | 3 | U |  |
| *PRKAG2* | 0.02 | 0.04 | 0.03 | 3.4 | U |  |
| *PRPF38B* | 0.02 | 0.02 | 0.01 | 4.2 | U |  |
| *PTGFR* | -0.12 | -0.22 | -0.13 | 5.5 | U |  |
| *PSAP* | 0.01 | 0.03 | 0.03 | 3.7 | U |  |
| *PSMD4* | -0.04 | -0.09 | -0.05 | 3.8 | U |  |
| *PTP4A2* | -0.10 | -0.14 | -0.08 | 3.1 | U |  |
| *PTPN13* | -0.14 | -0.28 | -0.19 | 4.2 | U |  |
| *PTPN11* | -0.03 | -0.05 | -0.03 | 3.1 | U |  |
| *PYGL* | 0.01 | 0.07 | 0.05 | 8.9 | U |  |
| *QSOX1* | -0.01 | -0.03 | -0.01 | 3.5 | U |  |
| *R3HCC1* | -0.12 | -0.20 | -0.15 | 4.2 | U |  |
| *RAB7A* | 0.00 | 0.01 | 0.02 | 3.3 | U |  |
| *RAD50* | -0.02 | -0.03 | -0.01 | 3.1 | U |  |
| *RASA2* | 0.06 | 0.15 | 0.10 | 5.5 | U |  |
| *RBFOX2* | 0.01 | -0.01 | -0.01 | 3.4 | U |  |
| *RBM25* | 0.01 | 0.04 | 0.01 | 4.4 | U |  |
| *RBM5* | 0.05 | 0.06 | 0.03 | 4 | U |  |
| *RCAN3* | -0.07 | -0.10 | -0.06 | 4.1 | U |  |
| *RCN3* | 0.06 | 0.09 | 0.04 | 3.6 | U |  |
| *RDH11* | -0.08 | -0.13 | -0.09 | 3.1 | U |  |
| *RGN* | -0.17 | -0.26 | -0.18 | 9.8 | U |  |
| *RHBDD2* | -0.03 | -0.06 | -0.03 | 3.8 | U |  |
| *RNASEK* | -0.03 | -0.12 | -0.09 | 3.6 | U |  |
| *RNF128* | -0.11 | -0.15 | -0.10 | 4.5 | U |  |
| *RNF144B* | -0.10 | -0.18 | -0.12 | 4.3 | U |  |
| *RNF20* | 0.02 | 0.00 | -0.01 | 3.3 | U |  |
| *RNF213* | -0.08 | -0.09 | -0.06 | 5.9 | U |  |
| *ROBO1* | -0.05 | -0.14 | -0.10 | 3.1 | U |  |
| *ROBO2* | -0.03 | -0.07 | -0.05 | 6.7 | U |  |
| *ROCK1* | 0.03 | 0.08 | 0.05 | 3.1 | U |  |
| *RRBP1* | -0.03 | -0.05 | -0.04 | 4.2 | U |  |
| *RSRC2* | 0.06 | 0.05 | 0.01 | 5.1 | U |  |
| *SAFB2* | 0.04 | 0.06 | 0.04 | 4.9 | U |  |
| *SCD5* | -0.14 | -0.22 | -0.12 | 4.2 | U |  |
| *SCYL2* | -0.01 | -0.07 | -0.05 | 4 | U |  |
| *SDC2* | 0.06 | 0.04 | 0.05 | 3.8 | U |  |
| *SEC63* | 0.01 | -0.03 | -0.02 | 3.4 | U |  |
| *SEMA6A* | -0.10 | -0.12 | -0.07 | 3.3 | U |  |
| *SEMA6D* | -0.07 | -0.11 | -0.07 | 6.2 | U |  |
| *SERPINA5* | -0.07 | -0.11 | -0.08 | 11.2 | U |  |
| *SF3B1* | 0.06 | 0.06 | 0.03 | 4.6 | U |  |
| *SGSH* | -0.04 | -0.12 | -0.10 | 3.6 | U |  |
| *SLC25A12* | -0.03 | 0.03 | 0.03 | 3.6 | U |  |
| *SLC25A28* | -0.15 | -0.26 | -0.18 | 5.8 | U |  |
| *SLC26A11* | -0.08 | -0.12 | -0.05 | 3.6 | U |  |
| *SLC27A3* | -0.08 | -0.18 | -0.15 | 5.7 | U |  |
| *SLC39A8* | -0.07 | -0.11 | -0.07 | 9.1 | U |  |
| *SLC40A1* | 0.01 | 0.02 | 0.03 | 5.3 | U |  |
| *SLITRK2* | 0.08 | 0.09 | 0.07 | 5.1 | U |  |
| *SLMAP* | -0.15 | -0.25 | -0.18 | 5.1 | U |  |
| *SNTB2* | -0.01 | -0.09 | -0.05 | 3.4 | U |  |
| *SON* | -0.02 | -0.05 | -0.03 | 4.2 | U |  |
| *SPG7* | -0.02 | -0.07 | -0.06 | 4.2 | U |  |
| *SPOCK2* | 0.04 | 0.04 | 0.01 | 22 | U |  |
| *STAR* | 0.01 | 0.05 | 0.05 | 3.4 | U |  |
| *STBD1* | -0.07 | -0.10 | -0.07 | 3.1 | U |  |
| *STIM1* | -0.01 | -0.04 | -0.04 | 3.2 | U |  |
| *STRA6* | -0.02 | 0.01 | 0.00 | 4.2 | U |  |
| *SUSD4* | -0.01 | -0.08 | -0.07 | 3.5 | U |  |
| *SYNCRIP* | 0.02 | 0.09 | 0.06 | 3.6 | U |  |
| *TACC1* | 0.01 | -0.01 | 0.00 | 3.5 | U |  |
| *TBC1D5* | -0.02 | -0.09 | -0.07 | 3 | U |  |
| *TBCEL* | -0.08 | -0.18 | -0.12 | 4.8 | U |  |
| *TET2* | 0.00 | -0.02 | -0.03 | 3.7 | U |  |
| *TFCP2* | -0.02 | -0.05 | -0.03 | 3.1 | U |  |
| *TFR2* | -0.04 | -0.09 | -0.07 | 3.3 | U |  |
| *THBS3* | -0.01 | -0.09 | -0.07 | 3.5 | U |  |
| *TIA1* | 0.08 | 0.13 | 0.06 | 3.9 | U |  |
| *TIMP2* | -0.06 | -0.10 | -0.07 | 7.9 | U |  |
| *TM2D1* | -0.09 | -0.17 | -0.11 | 3.1 | U |  |
| *TM9SF4* | -0.05 | -0.10 | -0.06 | 3.5 | U |  |
| *TMEM176A* | 0.02 | 0.09 | 0.07 | 4.1 | U |  |
| *TMEM176B* | 0.04 | 0.13 | 0.08 | 3.7 | U |  |
| *TMEM47* | -0.10 | -0.18 | -0.10 | 4.5 | U |  |
| *TMEM50B* | -0.01 | -0.03 | -0.01 | 3.2 | U |  |
| *TNFAIP6* | -0.15 | -0.20 | -0.12 | 279.6 | U |  |
| *TNFAIP8L3* | -0.08 | -0.16 | -0.10 | 4.7 | U |  |
| *TNPO1* | -0.04 | -0.05 | -0.03 | 5.5 | U |  |
| *TNS3* | -0.10 | -0.16 | -0.11 | 3.6 | U |  |
| *TOB1* | 0.01 | -0.07 | -0.05 | 6.3 | U |  |
| *TOPORS* | 0.04 | 0.02 | 0.01 | 3.3 | U |  |
| *TOX* | -0.01 | -0.05 | -0.01 | 11.4 | U |  |
| *TPR* | 0.03 | 0.06 | 0.03 | 4.3 | U |  |
| *TRIB2* | -0.09 | -0.15 | -0.09 | 3.2 | U |  |
| *TRIM25* | -0.01 | -0.01 | -0.01 | 3.7 | U |  |
| *TSPAN9* | -0.06 | -0.14 | -0.10 | 3.4 | U |  |
| *TTC3* | 0.01 | -0.01 | -0.01 | 3.1 | U |  |
| *TXNIP* | 0.05 | 0.09 | 0.06 | 4.1 | U |  |
| *UBQLN1* | -0.02 | -0.04 | -0.03 | 3 | U |  |
| *UBR1* | 0.00 | -0.04 | -0.03 | 4 | U |  |
| *UBR3* | -0.05 | -0.09 | -0.05 | 3.2 | U |  |
| *UGCG* | -0.03 | -0.02 | 0.00 | 4 | U |  |
| *UHRF1BP1L* | -0.02 | -0.04 | -0.01 | 3.5 | U |  |
| *USHBP1* | -0.07 | -0.12 | -0.09 | 5.2 | U |  |
| *USP4* | -0.05 | -0.12 | -0.07 | 4 | U |  |
| *USP7* | 0.00 | -0.01 | -0.01 | 6.4 | U |  |
| *UTP6* | 0.03 | 0.04 | 0.02 | 4.8 | U |  |
| *VCAN* | -0.09 | -0.15 | -0.10 | 5 | U |  |
| *VWA1* | -0.04 | -0.12 | -0.10 | 3 | U |  |
| *WDFY2* | -0.10 | -0.16 | -0.10 | 3.4 | U |  |
| *WDFY4* | 0.04 | 0.12 | 0.10 | 3.9 | U |  |
| *WHSC1L1* | 0.03 | 0.01 | -0.01 | 3.9 | U |  |
| *XRN2* | 0.04 | 0.06 | 0.05 | 3.1 | U |  |
| *YPEL5* | -0.01 | -0.10 | -0.07 | 3.5 | U |  |
| *ZBTB33* | -0.01 | -0.03 | 0.00 | 3 | U |  |
| *ZNF24* | -0.04 | -0.08 | -0.05 | 3.3 | U |  |
| *ZNF292* | -0.05 | -0.10 | -0.08 | 6 | U |  |
| *ZNF317* | -0.01 | -0.03 | -0.03 | 3.8 | U |  |
| *ZNF317* | -0.01 | -0.03 | -0.03 | 3.8 | U |  |
| *ZNF317* | -0.01 | -0.03 | -0.03 | 3.8 | U |  |
| *ZNF317* | -0.01 | -0.03 | -0.03 | 3.8 | U |  |
| *ZNF462* | 0.04 | 0.01 | 0.01 | 3.1 | U |  |
| *ZNF609* | -0.04 | -0.10 | -0.10 | 3.6 | U |  |

The first three columns **BL rate FC**, **Kinetic FC** and **Morphology FC** are the regression coefficients obtained in our analysis, **FC** is FC observed in (Hatzirodos et al. 2014a) and **trend** indicates the direction of the FC, U stands for up-regulated and D for down-regulated in big antral follicles respect to small antral follicles. The red color indicates that the expression trend in IVP scores is up-regulated as in (Hatzirodos et al. 2014a), green color indicates that the expression in our result is down-regulated as in (Hatzirodos et al. 2014a), yellow is used when our results showed opposite behavior from (Hatzirodos et al. 2014a).

# Table J. GSEA enrichment using KEGG database for negative enrichment scores for BL rate.

| NAME | SIZE | ES | NES | NOM p-val | FDR q-val |
| --- | --- | --- | --- | --- | --- |
| KEGG CELL CYCLE | 105.00 | -0.44 | -1.86 | 0.00E+00 | 6.7E-02 |
| KEGG PROGESTERONE MEDIATED OOCYTE MATURATION | 61.00 | -0.48 | -1.82 | 0.00E+00 | 4.7E-02 |
| KEGG N GLYCAN BIOSYNTHESIS | 38.00 | -0.49 | -1.74 | 3.55E-03 | 7.2E-02 |
| KEGG GLYCOLYSIS GLUCONEOGENESIS | 31.00 | -0.53 | -1.74 | 3.54E-03 | 5.5E-02 |
| KEGG OOCYTE MEIOSIS | 79.00 | -0.43 | -1.71 | 0.00E+00 | 5.5E-02 |
| KEGG DNA REPLICATION | 32.00 | -0.51 | -1.66 | 5.73E-03 | 7.7E-02 |
| KEGG HOMOLOGOUS RECOMBINATION | 24.00 | -0.52 | -1.63 | 1.04E-02 | 9.4E-02 |
| KEGG TYROSINE METABOLISM | 19.00 | -0.56 | -1.62 | 2.56E-02 | 8.7E-02 |
| KEGG GALACTOSE METABOLISM | 17.00 | -0.54 | -1.53 | 4.10E-02 | 1.7E-01 |
| KEGG FRUCTOSE AND MANNOSE METABOLISM | 28.00 | -0.48 | -1.52 | 1.94E-02 | 1.6E-01 |
| KEGG DRUG METABOLISM OTHER ENZYMES | 15.00 | -0.55 | -1.51 | 4.53E-02 | 1.6E-01 |
| KEGG PENTOSE PHOSPHATE PATHWAY | 20.00 | -0.51 | -1.50 | 4.29E-02 | 1.6E-01 |
| KEGG BIOSYNTHESIS OF UNSATURATED FATTY ACIDS | 15.00 | -0.53 | -1.46 | 6.36E-02 | 2.0E-01 |

KEGG entries are considered suggestive at FDR <25%. **Name:** name of the KEGG gene set enriched, **Size**: total number of genes in the gene set, **ES:** Enrichment Score, **NES:** Normalized Enrichment Score, **NOM p-val** = nominal P value, **FDR q-val:** False Discovery Rate associated with the entry.

# Table K. GSEA enrichment using KEGG database for positive enrichment scores for BL rate.

| NAME | SIZE | ES | NES | NOM p-val | FDR q-val |
| --- | --- | --- | --- | --- | --- |
| KEGG RIBOSOME | 45 | 0.62 | 2.34 | 0.00E+00 | 0.00E+00 |
| KEGG PATHOGENIC ESCHERICHIA COLI INFECTION | 39 | 0.56 | 2.01 | 0.00E+00 | 4.76E-03 |
| KEGG VIRAL MYOCARDITIS | 22 | 0.54 | 1.70 | 4.38E-03 | 1.35E-01 |
| KEGG CYTOKINE CYTOKINE RECEPTOR INTERACTION | 54 | 0.43 | 1.65 | 2.38E-03 | 1.54E-01 |
| KEGG CELL ADHESION MOLECULES CAMS | 37 | 0.46 | 1.63 | 7.25E-03 | 1.53E-01 |
| KEGG COMPLEMENT AND COAGULATION CASCADES | 20 | 0.51 | 1.57 | 3.15E-02 | 2.02E-01 |

KEGG entries are considered suggestive at FDR <25%. **Name:** name of the KEGG gene set enriched, **Size**: total number of genes in the gene set, **ES:** Enrichment Score, **NES:** Normalized Enrichment Score, **NOM p-val** = nominal P value, **FDR q-val:** False Discovery Rate associated with the entry.

# Table L. GSEA enrichment using KEGG database for positive enrichment scores for Kinetic.

| NAME | SIZE | ES | NES | NOM p-val | FDR q-val |
| --- | --- | --- | --- | --- | --- |
| KEGG_PATHOGENIC_ESCHERICHIA_COLI_INFECTION | 39 | 0.65 | 2.38 | 0.00E+00 | 0.00E+00 |
| KEGG_CYTOKINE_CYTOKINE_RECEPTOR_INTERACTION | 54 | 0.51 | 2.02 | 0.00E+00 | 7.60E-03 |
| KEGG_CELL_ADHESION_MOLECULES_CAMS | 37 | 0.54 | 1.97 | 0.00E+00 | 1.00E-02 |
| KEGG_SPLICEOSOME | 110 | 0.43 | 1.95 | 0.00E+00 | 9.33E-03 |
| KEGG_SYSTEMIC_LUPUS_ERYTHEMATOSUS | 40 | 0.52 | 1.95 | 0.00E+00 | 8.24E-03 |
| KEGG_DNA_REPLICATION | 32 | 0.55 | 1.92 | 2.32E-03 | 1.13E-02 |
| KEGG_VIRAL_MYOCARDITIS | 22 | 0.58 | 1.81 | 9.43E-03 | 2.25E-02 |
| KEGG_HEMATOPOIETIC_CELL_LINEAGE | 20 | 0.59 | 1.81 | 0.00E+00 | 1.97E-02 |
| KEGG_RIG_I_LIKE_RECEPTOR_SIGNALING_PATHWAY | 41 | 0.49 | 1.80 | 0.00E+00 | 1.96E-02 |
| KEGG_COMPLEMENT_AND_COAGULATION_CASCADES | 20 | 0.57 | 1.76 | 4.47E-03 | 2.78E-02 |
| KEGG_CYTOSOLIC_DNA_SENSING_PATHWAY | 29 | 0.52 | 1.76 | 2.33E-03 | 2.67E-02 |
| KEGG_LEUKOCYTE_TRANSENDOTHELIAL_MIGRATION | 53 | 0.43 | 1.71 | 2.38E-03 | 4.14E-02 |
| KEGG_ANTIGEN_PROCESSING_AND_PRESENTATION | 30 | 0.50 | 1.70 | 1.58E-02 | 4.11E-02 |
| KEGG_NATURAL_KILLER_CELL_MEDIATED_CYTOTOXICITY | 43 | 0.45 | 1.67 | 5.18E-03 | 4.71E-02 |
| KEGG_ECM_RECEPTOR_INTERACTION | 44 | 0.44 | 1.65 | 1.17E-02 | 5.11E-02 |
| KEGG_LEISHMANIA_INFECTION | 33 | 0.46 | 1.63 | 1.62E-02 | 5.75E-02 |
| KEGG_ARRHYTHMOGENIC_RIGHT_VENTRICULAR_CARDIOMYOPATHY_ARVC | 33 | 0.44 | 1.58 | 1.56E-02 | 7.82E-02 |
| KEGG_CYSTEINE_AND_METHIONINE_METABOLISM | 27 | 0.47 | 1.58 | 2.99E-02 | 7.78E-02 |
| KEGG_REGULATION_OF_ACTIN_CYTOSKELETON | 126 | 0.32 | 1.47 | 7.58E-03 | 1.62E-01 |
| KEGG_FATTY_ACID_METABOLISM | 30 | 0.41 | 1.44 | 5.05E-02 | 1.91E-01 |
| KEGG_P53_SIGNALING_PATHWAY | 51 | 0.37 | 1.44 | 3.76E-02 | 1.85E-01 |
| KEGG_TOLL_LIKE_RECEPTOR_SIGNALING_PATHWAY | 56 | 0.35 | 1.42 | 3.05E-02 | 1.92E-01 |
| KEGG_MAPK_SIGNALING_PATHWAY | 151 | 0.30 | 1.41 | 1.20E-02 | 1.92E-01 |
| KEGG_GAP_JUNCTION | 52 | 0.36 | 1.41 | 4.16E-02 | 1.86E-01 |
| KEGG_NOD_LIKE_RECEPTOR_SIGNALING_PATHWAY | 32 | 0.40 | 1.36 | 8.39E-02 | 2.42E-01 |
| KEGG_FOCAL_ADHESION | 125 | 0.30 | 1.36 | 1.69E-02 | 2.43E-01 |

KEGG entries are considered suggestive at FDR <25%. **Name:** name of the KEGG gene set enriched, **Size**: total number of genes in the gene set, **ES:** Enrichment Score, **NES:** Normalized Enrichment Score, **NOM p-val** = nominal P value, **FDR q-val:** False Discovery Rate associated with the entry.

# Table M. GSEA enrichment using KEGG database for positive enrichment scores for Morphology.

| NAME | SIZE | ES | NES | NOM p-val | FDR q-val |
| --- | --- | --- | --- | --- | --- |
| KEGG_DNA_REPLICATION | 32 | 0.68 | 2.39 | 0.00E+00 | 0.00E+00 |
| KEGG_PATHOGENIC_ESCHERICHIA_COLI_INFECTION | 39 | 0.63 | 2.27 | 0.00E+00 | 0.00E+00 |
| KEGG_CYTOKINE_CYTOKINE_RECEPTOR_INTERACTION | 54 | 0.52 | 2.12 | 0.00E+00 | 2.08E-03 |
| KEGG_ANTIGEN_PROCESSING_AND_PRESENTATION | 30 | 0.60 | 2.05 | 0.00E+00 | 3.12E-03 |
| KEGG_SYSTEMIC_LUPUS_ERYTHEMATOSUS | 40 | 0.54 | 1.98 | 0.00E+00 | 5.43E-03 |
| KEGG_HEMATOPOIETIC_CELL_LINEAGE | 20 | 0.62 | 1.96 | 0.00E+00 | 4.79E-03 |
| KEGG_NATURAL_KILLER_CELL_MEDIATED_CYTOTOXICITY | 43 | 0.51 | 1.93 | 0.00E+00 | 8.36E-03 |
| KEGG_CYSTEINE_AND_METHIONINE_METABOLISM | 27 | 0.57 | 1.90 | 8.99E-03 | 9.31E-03 |
| KEGG_RIG_I_LIKE_RECEPTOR_SIGNALING_PATHWAY | 41 | 0.50 | 1.88 | 0.00E+00 | 9.69E-03 |
| KEGG_CELL_ADHESION_MOLECULES_CAMS | 37 | 0.50 | 1.85 | 2.35E-03 | 1.16E-02 |
| KEGG_SPLICEOSOME | 110 | 0.40 | 1.84 | 0.00E+00 | 1.18E-02 |
| KEGG_LEUKOCYTE_TRANSENDOTHELIAL_MIGRATION | 53 | 0.44 | 1.76 | 0.00E+00 | 2.69E-02 |
| KEGG_CYTOSOLIC_DNA_SENSING_PATHWAY | 29 | 0.50 | 1.72 | 9.28E-03 | 3.80E-02 |
| KEGG_TOLL_LIKE_RECEPTOR_SIGNALING_PATHWAY | 56 | 0.43 | 1.69 | 0.00E+00 | 4.36E-02 |
| KEGG_AMINO_SUGAR_AND_NUCLEOTIDE_SUGAR_METABOLISM | 38 | 0.45 | 1.65 | 4.45E-03 | 6.08E-02 |
| KEGG_VIRAL_MYOCARDITIS | 22 | 0.50 | 1.63 | 2.16E-02 | 6.62E-02 |
| KEGG_P53_SIGNALING_PATHWAY | 51 | 0.41 | 1.54 | 2.16E-02 | 1.16E-01 |
| KEGG_NOD_LIKE_RECEPTOR_SIGNALING_PATHWAY | 32 | 0.43 | 1.53 | 2.10E-02 | 1.19E-01 |
| KEGG_COMPLEMENT_AND_COAGULATION_CASCADES | 20 | 0.50 | 1.53 | 2.82E-02 | 1.16E-01 |
| KEGG_MISMATCH_REPAIR | 21 | 0.48 | 1.52 | 3.32E-02 | 1.18E-01 |
| KEGG_LYSOSOME | 97 | 0.34 | 1.51 | 1.62E-02 | 1.24E-01 |
| KEGG_GAP_JUNCTION | 52 | 0.38 | 1.50 | 1.36E-02 | 1.20E-01 |
| KEGG_GLYCINE_SERINE_AND_THREONINE_METABOLISM | 17 | 0.50 | 1.49 | 7.22E-02 | 1.28E-01 |
| KEGG_SELENOAMINO_ACID_METABOLISM | 18 | 0.49 | 1.47 | 5.29E-02 | 1.38E-01 |
| KEGG_BUTANOATE_METABOLISM | 22 | 0.46 | 1.46 | 7.36E-02 | 1.51E-01 |
| KEGG_NUCLEOTIDE_EXCISION_REPAIR | 39 | 0.40 | 1.45 | 3.16E-02 | 1.48E-01 |
| KEGG_MAPK_SIGNALING_PATHWAY | 151 | 0.30 | 1.44 | 9.88E-03 | 1.53E-01 |
| KEGG_PROTEASOME | 39 | 0.38 | 1.42 | 4.66E-02 | 1.66E-01 |
| KEGG_BASE_EXCISION_REPAIR | 32 | 0.40 | 1.42 | 5.99E-02 | 1.62E-01 |
| KEGG_LEISHMANIA_INFECTION | 33 | 0.41 | 1.42 | 5.47E-02 | 1.59E-01 |
| KEGG_ECM_RECEPTOR_INTERACTION | 44 | 0.38 | 1.41 | 3.71E-02 | 1.67E-01 |
| KEGG_DILATED_CARDIOMYOPATHY | 31 | 0.40 | 1.40 | 6.72E-02 | 1.64E-01 |
| KEGG_REGULATION_OF_ACTIN_CYTOSKELETON | 126 | 0.29 | 1.36 | 2.81E-02 | 2.03E-01 |
| KEGG_ARRHYTHMOGENIC_RIGHT_VENTRICULAR_CARDIOMYOPATHY_ARVC | 33 | 0.38 | 1.35 | 9.73E-02 | 2.10E-01 |
| KEGG_FC_GAMMA_R_MEDIATED_PHAGOCYTOSIS | 62 | 0.33 | 1.34 | 5.68E-02 | 2.18E-01 |

KEGG entries are considered suggestive at FDR <25%. **Name:** name of the KEGG gene set enriched, **Size**: total number of genes in the gene set, **ES:** Enrichment Score, **NES:** Normalized Enrichment Score, **NOM p-val** = nominal P value, **FDR q-val:** False Discovery Rate associated with the entry.

# Table N. GSEA enrichment using GO terms for positive enrichment scores for BL rate.

| NAME | SIZE | ES | NES | NOM p-val | FDR q-val |
| --- | --- | --- | --- | --- | --- |
| STRUCTURAL_CONSTITUENT_OF_RIBOSOME | 46 | 0.57 | 2.13 | 0.00E+00 | 9.26E-03 |
| RNA_BINDING | 185 | 0.42 | 2.00 | 0.00E+00 | 3.54E-02 |
| RESPONSE_TO_VIRUS | 19 | 0.62 | 1.91 | 0.00E+00 | 7.48E-02 |
| STRUCTURAL_MOLECULE_ACTIVITY | 111 | 0.40 | 1.78 | 0.00E+00 | 2.11E-01 |

GO terms are considered suggestive at FDR <25%. **Name:** name of the GO term of the gene set enriched, **Size**: total number of genes in the gene set, **ES:** Enrichment Score, **NES:** Normalized Enrichment Score, **NOM p-val** = nominal P value, **FDR q-val:** False Discovery Rate associated with the entry.

# Table O. GSEA enrichment using GO terms for negative enrichment scores for BL rate.

| NAME | SIZE | ES | NES | NOM p-val | FDR q-val |
| --- | --- | --- | --- | --- | --- |
| M_PHASE | 87 | -0.53 | -2.14 | 0.00E+00 | 4.23E-03 |
| CELL_CYCLE_PROCESS | 150 | -0.48 | -2.14 | 0.00E+00 | 2.11E-03 |
| MITOSIS | 67 | -0.54 | -2.13 | 0.00E+00 | 1.41E-03 |
| M_PHASE_OF_MITOTIC_CELL_CYCLE | 70 | -0.54 | -2.12 | 0.00E+00 | 1.06E-03 |
| SPINDLE | 34 | -0.63 | -2.11 | 0.00E+00 | 8.45E-04 |
| CELL_CYCLE_PHASE | 131 | -0.48 | -2.10 | 0.00E+00 | 1.24E-03 |
| MITOTIC_CELL_CYCLE | 124 | -0.48 | -2.07 | 0.00E+00 | 2.28E-03 |
| REGULATION_OF_MITOSIS | 29 | -0.60 | -1.90 | 0.00E+00 | 2.87E-02 |
| MICROTUBULE_CYTOSKELETON_ORGANIZATION_AND_BIOGENESIS | 30 | -0.57 | -1.84 | 1.82E-03 | 5.66E-02 |
| CELLULAR_PROTEIN_CATABOLIC_PROCESS | 53 | -0.49 | -1.82 | 0.00E+00 | 6.67E-02 |
| CELL_CYCLE_GO_0007049 | 244 | -0.37 | -1.73 | 0.00E+00 | 1.44E-01 |
| LIPID_BIOSYNTHETIC_PROCESS | 71 | -0.44 | -1.73 | 3.50E-03 | 1.40E-01 |
| SPINDLE_POLE | 16 | -0.61 | -1.72 | 9.23E-03 | 1.33E-01 |
| KINASE_BINDING | 51 | -0.46 | -1.72 | 0.00E+00 | 1.35E-01 |
| ALCOHOL_METABOLIC_PROCESS | 51 | -0.47 | -1.70 | 5.45E-03 | 1.48E-01 |
| PROTEIN_UBIQUITINATION | 37 | -0.49 | -1.69 | 3.60E-03 | 1.46E-01 |
| MICROTUBULE_CYTOSKELETON | 126 | -0.39 | -1.68 | 1.64E-03 | 1.60E-01 |
| OXIDOREDUCTASE_ACTIVITY_ACTING_ON_NADH_OR_NADPH | 16 | -0.59 | -1.67 | 1.48E-02 | 1.65E-01 |
| CELLULAR_MACROMOLECULE_CATABOLIC_PROCESS | 84 | -0.41 | -1.66 | 1.70E-03 | 1.71E-01 |
| PROTEIN_CATABOLIC_PROCESS | 59 | -0.43 | -1.65 | 3.68E-03 | 1.73E-01 |
| CHROMOSOMEPERICENTRIC_REGION | 25 | -0.51 | -1.62 | 1.06E-02 | 2.13E-01 |
| G1_S_TRANSITION_OF_MITOTIC_CELL_CYCLE | 21 | -0.54 | -1.62 | 1.93E-02 | 2.20E-01 |
| EMBRYONIC_DEVELOPMENT | 24 | -0.51 | -1.61 | 1.29E-02 | 2.16E-01 |
| PROTEIN_MODIFICATION_BY_SMALL_PROTEIN_CONJUGATION | 40 | -0.47 | -1.61 | 1.23E-02 | 2.20E-01 |
| INTERPHASE | 55 | -0.43 | -1.60 | 1.18E-02 | 2.22E-01 |
| SISTER_CHROMATID_SEGREGATION | 15 | -0.58 | -1.58 | 2.68E-02 | 2.49E-01 |
| STEROID_METABOLIC_PROCESS | 29 | -0.48 | -1.58 | 2.48E-02 | 2.49E-01 |
| CELLULAR_LIPID_METABOLIC_PROCESS | 149 | -0.36 | -1.57 | 1.71E-03 | 2.42E-01 |

GO terms are considered suggestive at FDR <25%. **Name:** name of the GO term of the gene set enriched, **Size**: total number of genes in the gene set, **ES:** Enrichment Score, **NES:** Normalized Enrichment Score, **NOM p-val** = nominal P value, **FDR q-val:** False Discovery Rate associated with the entry.

# Table P. GSEA enrichment using GO terms for positive enrichment scores for Kinetic.

| NAME | SIZE | ES | NES | NOM p-val | FDR q-val |
| --- | --- | --- | --- | --- | --- |
| RESPONSE_TO_VIRUS | 19 | 0.68 | 2.05 | 0.00E+00 | 5.10E-02 |
| MRNA_BINDING | 17 | 0.67 | 1.98 | 0.00E+00 | 6.68E-02 |
| CHROMATIN | 26 | 0.60 | 1.97 | 0.00E+00 | 4.85E-02 |
| RESPONSE_TO_OTHER_ORGANISM | 25 | 0.59 | 1.96 | 0.00E+00 | 4.37E-02 |
| NUCLEAR_CHROMOSOME_PART | 26 | 0.56 | 1.90 | 0.00E+00 | 6.61E-02 |
| STRUCTURAL_CONSTITUENT_OF_CYTOSKELETON | 23 | 0.59 | 1.88 | 2.17E-03 | 6.72E-02 |
| RNA_BINDING | 185 | 0.38 | 1.86 | 0.00E+00 | 7.49E-02 |
| LOCOMOTORY_BEHAVIOR | 18 | 0.62 | 1.82 | 2.24E-03 | 1.01E-01 |
| IMMUNE_RESPONSE | 66 | 0.44 | 1.80 | 0.00E+00 | 1.10E-01 |
| MRNA_PROCESSING_GO_0006397 | 67 | 0.43 | 1.79 | 0.00E+00 | 1.04E-01 |
| MRNA_METABOLIC_PROCESS | 77 | 0.43 | 1.79 | 0.00E+00 | 9.55E-02 |
| NUCLEAR_CHROMOSOME | 41 | 0.49 | 1.79 | 2.47E-03 | 8.92E-02 |
| JAK_STAT_CASCADE | 18 | 0.58 | 1.74 | 6.68E-03 | 1.27E-01 |
| RNA_SPLICING | 85 | 0.40 | 1.72 | 0.00E+00 | 1.35E-01 |
| ACTIN_CYTOSKELETON | 86 | 0.40 | 1.71 | 0.00E+00 | 1.41E-01 |
| CYTOKINE_BINDING | 16 | 0.58 | 1.71 | 6.90E-03 | 1.37E-01 |
| CELL_ACTIVATION | 22 | 0.53 | 1.70 | 4.42E-03 | 1.40E-01 |
| ACTIN_BINDING | 51 | 0.43 | 1.68 | 4.80E-03 | 1.53E-01 |
| INTEGRAL_TO_PLASMA_MEMBRANE | 295 | 0.33 | 1.67 | 0.00E+00 | 1.61E-01 |
| INTRINSIC_TO_PLASMA_MEMBRANE | 301 | 0.32 | 1.66 | 0.00E+00 | 1.56E-01 |
| LEUKOCYTE_ACTIVATION | 21 | 0.52 | 1.66 | 1.30E-02 | 1.60E-01 |
| NEGATIVE_REGULATION_OF_PROGRAMMED_CELL_DEATH | 91 | 0.38 | 1.65 | 5.06E-03 | 1.64E-01 |
| PLASMA_MEMBRANE_PART | 371 | 0.31 | 1.64 | 0.00E+00 | 1.70E-01 |
| BEHAVIOR | 33 | 0.47 | 1.63 | 1.23E-02 | 1.67E-01 |
| RNA_PROCESSING | 156 | 0.35 | 1.63 | 0.00E+00 | 1.67E-01 |
| SOLUBLE_FRACTION | 76 | 0.38 | 1.62 | 5.08E-03 | 1.76E-01 |
| CYSTEINE_TYPE_ENDOPEPTIDASE_ACTIVITY | 27 | 0.48 | 1.60 | 2.93E-02 | 1.90E-01 |
| PLASMA_MEMBRANE | 494 | 0.30 | 1.60 | 0.00E+00 | 1.84E-01 |
| LYMPHOCYTE_ACTIVATION | 21 | 0.52 | 1.59 | 2.04E-02 | 1.95E-01 |
| RECEPTOR_BINDING | 145 | 0.33 | 1.58 | 0.00E+00 | 2.00E-01 |
| ACTIN_FILAMENT_BASED_PROCESS | 82 | 0.36 | 1.58 | 1.27E-02 | 2.00E-01 |
| CELL_SURFACE_RECEPTOR_LINKED_SIGNAL_TRANSDUCTION_GO_0007166 | 201 | 0.32 | 1.58 | 0.00E+00 | 1.95E-01 |
| MONOVALENT_INORGANIC_CATION_TRANSPORT | 20 | 0.51 | 1.57 | 1.63E-02 | 1.94E-01 |
| IMMUNE_SYSTEM_PROCESS | 114 | 0.35 | 1.57 | 5.46E-03 | 1.95E-01 |
| ENDOPEPTIDASE_ACTIVITY | 49 | 0.41 | 1.55 | 1.98E-02 | 2.17E-01 |
| NEGATIVE_REGULATION_OF_APOPTOSIS | 90 | 0.36 | 1.55 | 5.10E-03 | 2.11E-01 |
| ACTIN_CYTOSKELETON_ORGANIZATION_AND_BIOGENESIS | 76 | 0.37 | 1.55 | 5.00E-03 | 2.07E-01 |
| INTRACELLULAR_NON_MEMBRANE_BOUND_ORGANELLE | 451 | 0.29 | 1.54 | 0.00E+00 | 2.14E-01 |
| SYNAPSE | 16 | 0.52 | 1.53 | 4.20E-02 | 2.22E-01 |
| RECEPTOR_COMPLEX | 19 | 0.50 | 1.53 | 4.27E-02 | 2.23E-01 |
| REPLICATION_FORK | 17 | 0.51 | 1.53 | 3.70E-02 | 2.19E-01 |
| NUCLEASE_ACTIVITY | 38 | 0.42 | 1.53 | 2.12E-02 | 2.14E-01 |
| MOTOR_ACTIVITY | 18 | 0.50 | 1.53 | 4.87E-02 | 2.11E-01 |
| TRNA_METABOLIC_PROCESS | 18 | 0.51 | 1.52 | 3.42E-02 | 2.14E-01 |
| STRUCTURAL_MOLECULE_ACTIVITY | 111 | 0.34 | 1.52 | 0.00E+00 | 2.11E-01 |
| CHROMOSOMAL_PART | 77 | 0.36 | 1.52 | 1.85E-02 | 2.09E-01 |
| RIBONUCLEOPROTEIN_COMPLEX | 122 | 0.33 | 1.52 | 2.67E-03 | 2.07E-01 |
| NON_MEMBRANE_BOUND_ORGANELLE | 451 | 0.29 | 1.51 | 0.00E+00 | 2.06E-01 |
| RESPONSE_TO_EXTERNAL_STIMULUS | 96 | 0.34 | 1.51 | 0.00E+00 | 2.07E-01 |
| NEGATIVE_REGULATION_OF_DNA_METABOLIC_PROCESS | 17 | 0.50 | 1.50 | 3.00E-02 | 2.09E-01 |
| SMALL_GTPASE_REGULATOR_ACTIVITY | 47 | 0.40 | 1.50 | 3.83E-02 | 2.08E-01 |
| CYTOKINESIS | 16 | 0.52 | 1.50 | 6.03E-02 | 2.12E-01 |
| ENDONUCLEASE_ACTIVITY | 20 | 0.49 | 1.49 | 4.58E-02 | 2.11E-01 |
| TRANSFERASE_ACTIVITY_TRANSFERRING_ONE_CARBON_GROUPS | 29 | 0.45 | 1.49 | 4.19E-02 | 2.07E-01 |
| CHROMOSOME | 99 | 0.33 | 1.47 | 2.65E-03 | 2.32E-01 |
| ANTI_APOPTOSIS | 75 | 0.35 | 1.47 | 9.46E-03 | 2.31E-01 |
| RAS_GTPASE_ACTIVATOR_ACTIVITY | 20 | 0.47 | 1.47 | 6.44E-02 | 2.39E-01 |
| RIBOSOME_BIOGENESIS_AND_ASSEMBLY | 15 | 0.51 | 1.46 | 8.99E-02 | 2.39E-01 |
| MULTI_ORGANISM_PROCESS | 49 | 0.38 | 1.46 | 3.08E-02 | 2.42E-01 |

GO terms are considered suggestive at FDR <25%. **Name:** name of the GO term of the gene set enriched, **Size**: total number of genes in the gene set, **ES:** Enrichment Score, **NES:** Normalized Enrichment Score, **NOM p-val** = nominal P value, **FDR q-val:** False Discovery Rate associated with the entry.

# Table Q. GSEA enrichment using GO terms for positive enrichment scores for Morphology.

| NAME | SIZE | ES | NES | NOM p-val | FDR q-val |
| --- | --- | --- | --- | --- | --- |
| RESPONSE_TO_VIRUS | 19 | 0.69 | 2.15 | 0.00E+00 | 1.13E-02 |
| IMMUNE_RESPONSE | 66 | 0.51 | 2.10 | 0.00E+00 | 1.71E-02 |
| RESPONSE_TO_OTHER_ORGANISM | 25 | 0.59 | 1.96 | 2.17E-03 | 5.62E-02 |
| NUCLEAR_CHROMOSOME_PART | 26 | 0.59 | 1.95 | 0.00E+00 | 4.57E-02 |
| LOCOMOTORY_BEHAVIOR | 18 | 0.64 | 1.93 | 2.34E-03 | 4.62E-02 |
| CHROMATIN | 26 | 0.60 | 1.93 | 0.00E+00 | 3.98E-02 |
| CELL_ACTIVATION | 22 | 0.58 | 1.86 | 6.58E-03 | 8.32E-02 |
| CYTOKINE_BINDING | 16 | 0.64 | 1.83 | 4.75E-03 | 9.96E-02 |
| NUCLEASE_ACTIVITY | 38 | 0.50 | 1.82 | 0.00E+00 | 9.65E-02 |
| INTRINSIC_TO_PLASMA_MEMBRANE | 301 | 0.35 | 1.82 | 0.00E+00 | 8.94E-02 |
| RNA_BINDING | 185 | 0.37 | 1.82 | 0.00E+00 | 8.35E-02 |
| INTEGRAL_TO_PLASMA_MEMBRANE | 295 | 0.35 | 1.81 | 0.00E+00 | 7.94E-02 |
| DNA_REPLICATION | 83 | 0.43 | 1.81 | 0.00E+00 | 7.36E-02 |
| DNA_DEPENDENT_DNA_REPLICATION | 46 | 0.48 | 1.81 | 2.43E-03 | 7.02E-02 |
| LYMPHOCYTE_ACTIVATION | 21 | 0.56 | 1.81 | 4.52E-03 | 6.62E-02 |
| IMMUNE_SYSTEM_PROCESS | 114 | 0.40 | 1.80 | 0.00E+00 | 6.56E-02 |
| LEUKOCYTE_ACTIVATION | 21 | 0.56 | 1.80 | 4.29E-03 | 6.47E-02 |
| NUCLEAR_CHROMOSOME | 41 | 0.49 | 1.80 | 2.27E-03 | 6.12E-02 |
| CHROMOSOMAL_PART | 77 | 0.42 | 1.79 | 2.38E-03 | 6.19E-02 |
| ACTIN_BINDING | 51 | 0.46 | 1.79 | 4.66E-03 | 5.96E-02 |
| MONOVALENT_INORGANIC_CATION_TRANSPORT | 20 | 0.57 | 1.76 | 2.16E-03 | 7.75E-02 |
| REPLICATION_FORK | 17 | 0.59 | 1.75 | 8.42E-03 | 7.53E-02 |
| SOLUBLE_FRACTION | 76 | 0.41 | 1.74 | 0.00E+00 | 7.81E-02 |
| MRNA_PROCESSING_GO_0006397 | 67 | 0.41 | 1.74 | 0.00E+00 | 7.68E-02 |
| INTRACELLULAR_NON_MEMBRANE_BOUND_ORGANELLE | 451 | 0.32 | 1.74 | 0.00E+00 | 7.53E-02 |
| MRNA_BINDING | 17 | 0.59 | 1.74 | 1.31E-02 | 7.38E-02 |
| SINGLE_STRANDED_DNA_BINDING | 27 | 0.51 | 1.73 | 0.00E+00 | 7.28E-02 |
| ENDOPEPTIDASE_ACTIVITY | 49 | 0.44 | 1.73 | 0.00E+00 | 7.10E-02 |
| NON_MEMBRANE_BOUND_ORGANELLE | 451 | 0.32 | 1.72 | 0.00E+00 | 7.37E-02 |
| PLASMA_MEMBRANE_PART | 371 | 0.32 | 1.71 | 0.00E+00 | 8.23E-02 |
| MRNA_METABOLIC_PROCESS | 77 | 0.40 | 1.70 | 2.33E-03 | 8.14E-02 |
| ACTIN_CYTOSKELETON | 86 | 0.40 | 1.70 | 0.00E+00 | 7.89E-02 |
| RIBONUCLEASE_ACTIVITY | 17 | 0.57 | 1.70 | 2.29E-02 | 7.66E-02 |
| DNA_REPLICATION_INITIATION | 15 | 0.58 | 1.69 | 1.67E-02 | 8.09E-02 |
| CHROMOSOME | 99 | 0.38 | 1.68 | 0.00E+00 | 8.49E-02 |
| JAK_STAT_CASCADE | 18 | 0.56 | 1.67 | 2.00E-02 | 9.24E-02 |
| NEGATIVE_REGULATION_OF_PROGRAMMED_CELL_DEATH | 91 | 0.38 | 1.67 | 0.00E+00 | 9.14E-02 |
| REGULATION_OF_DNA_REPLICATION | 16 | 0.56 | 1.66 | 2.52E-02 | 9.60E-02 |
| STRUCTURAL_CONSTITUENT_OF_CYTOSKELETON | 23 | 0.51 | 1.65 | 4.20E-03 | 9.72E-02 |
| RNA_PROCESSING | 156 | 0.35 | 1.65 | 0.00E+00 | 9.64E-02 |
| NEGATIVE_REGULATION_OF_DNA_METABOLIC_PROCESS | 17 | 0.56 | 1.64 | 1.21E-02 | 1.00E-01 |
| CYSTEINE_TYPE_ENDOPEPTIDASE_ACTIVITY | 27 | 0.48 | 1.63 | 1.13E-02 | 1.12E-01 |
| PLASMA_MEMBRANE | 494 | 0.30 | 1.62 | 0.00E+00 | 1.18E-01 |
| REGULATION_OF_DNA_METABOLIC_PROCESS | 34 | 0.46 | 1.62 | 2.44E-02 | 1.16E-01 |
| RIBONUCLEOPROTEIN_COMPLEX | 122 | 0.35 | 1.61 | 0.00E+00 | 1.18E-01 |
| RNA_SPLICING | 85 | 0.37 | 1.60 | 7.19E-03 | 1.24E-01 |
| TRANSFERASE_ACTIVITY_TRANSFERRING_ONE_CARBON_GROUPS | 29 | 0.46 | 1.60 | 1.59E-02 | 1.22E-01 |
| DNA_METABOLIC_PROCESS | 207 | 0.32 | 1.60 | 0.00E+00 | 1.20E-01 |
| BEHAVIOR | 33 | 0.45 | 1.60 | 1.51E-02 | 1.18E-01 |
| ENDONUCLEASE_ACTIVITY | 20 | 0.52 | 1.60 | 1.48E-02 | 1.17E-01 |
| RIBOSOME_BIOGENESIS_AND_ASSEMBLY | 15 | 0.56 | 1.60 | 1.91E-02 | 1.15E-01 |
| REGULATION_OF_IMMUNE_SYSTEM_PROCESS | 25 | 0.48 | 1.59 | 2.60E-02 | 1.21E-01 |
| CONDENSED_CHROMOSOME | 26 | 0.47 | 1.59 | 3.06E-02 | 1.20E-01 |
| NEGATIVE_REGULATION_OF_APOPTOSIS | 90 | 0.36 | 1.58 | 2.46E-03 | 1.25E-01 |
| RECEPTOR_COMPLEX | 19 | 0.50 | 1.57 | 1.74E-02 | 1.32E-01 |
| STRUCTURE_SPECIFIC_DNA_BINDING | 42 | 0.42 | 1.55 | 1.77E-02 | 1.53E-01 |
| RECEPTOR_MEDIATED_ENDOCYTOSIS | 18 | 0.51 | 1.55 | 3.31E-02 | 1.50E-01 |
| CELL_SURFACE_RECEPTOR_LINKED_SIGNAL_TRANSDUCTION_GO_0007166 | 201 | 0.32 | 1.54 | 0.00E+00 | 1.51E-01 |
| SMALL_GTPASE_REGULATOR_ACTIVITY | 47 | 0.40 | 1.54 | 1.96E-02 | 1.53E-01 |
| RHO_PROTEIN_SIGNAL_TRANSDUCTION | 24 | 0.46 | 1.53 | 4.00E-02 | 1.56E-01 |
| ION_TRANSPORT | 58 | 0.38 | 1.53 | 1.73E-02 | 1.55E-01 |
| METHYLTRANSFERASE_ACTIVITY | 28 | 0.44 | 1.53 | 2.83E-02 | 1.55E-01 |
| MULTI_ORGANISM_PROCESS | 49 | 0.39 | 1.51 | 3.00E-02 | 1.77E-01 |
| RESPONSE_TO_EXTERNAL_STIMULUS | 96 | 0.34 | 1.51 | 4.72E-03 | 1.76E-01 |
| RESPONSE_TO_BIOTIC_STIMULUS | 53 | 0.39 | 1.51 | 1.17E-02 | 1.75E-01 |
| CYTOSKELETON | 248 | 0.30 | 1.51 | 2.75E-03 | 1.74E-01 |
| NUCLEOBASENUCLEOSIDENUCLEOTIDE_KINASE_ACTIVITY | 19 | 0.49 | 1.50 | 4.31E-02 | 1.80E-01 |
| ANTI_APOPTOSIS | 75 | 0.35 | 1.48 | 1.43E-02 | 2.07E-01 |
| CYTOSKELETAL_PROTEIN_BINDING | 115 | 0.33 | 1.47 | 7.13E-03 | 2.07E-01 |
| RECEPTOR_BINDING | 145 | 0.30 | 1.45 | 9.55E-03 | 2.38E-01 |
| RNA_CATABOLIC_PROCESS | 17 | 0.48 | 1.44 | 6.26E-02 | 2.46E-01 |
| INTRINSIC_TO_MEMBRANE | 492 | 0.26 | 1.44 | 0.00E+00 | 2.48E-01 |
| ORGANELLE_ORGANIZATION_AND_BIOGENESIS | 364 | 0.27 | 1.43 | 0.00E+00 | 2.49E-01 |
| INTEGRAL_TO_MEMBRANE | 483 | 0.26 | 1.43 | 0.00E+00 | 2.48E-01 |

GO terms are considered suggestive at FDR <25%. **Name:** name of the GO term of the gene set enriched, **Size**: total number of genes in the gene set, **ES:** Enrichment Score, **NES:** Normalized Enrichment Score, **NOM p-val** = nominal P value, **FDR q-val:** False Discovery Rate associated with the entry.

# Table R. IPA overrepresented molecular and cellular processes for BL rate

| **Category** | **B-H p-value** | **Molecules** |
| --- | --- | --- |
| Cellular Growth and Proliferation | 4.52E-02-1.06E-01 | *PTGFR,ADAM17,FBXL7,MX1,PRPF8,KIAA2022,TRIB1,NOTCH2,TNKS,GADD45A,CASQ1,NTRK1,CLMN,HEY2,ZFHX3,PAPPA,TNFAIP6,ERAP1,IFNAR2,P3H2,STC1,CTSL,RND3,DDX58,RGN,ADIPOR2,TMOD1,APOD,MAOA* |
| Cellular Development | 4.52E-02-1.14E-01 | *PTGFR,PAPPA,ADAM17,MEX3C,TNFAIP6,IL17RD,KIAA2022,STC1,TRIB1,TNKS,NOTCH2,RND3,RDH14,CASQ1,DDX58,NTRK1,RGN,DUSP7,BTBD7,HEY2,TMOD1,ITGB5,ZFHX3,MAOA,APOD* |
| Amino Acid Metabolism | 4.52E-02-8.21E-02 | *BCAT2,OAT,RGN,GOT1* |
| Gene Expression | 4.52E-02-1.06E-01 | *PTGFR,NOTCH2,BCAT2,GADD45A,DDX58,IRF9* |
| Cell Death and Survival | 4.52E-02-1.14E-01 | *PTGFR,ADAM17,MX1,IL17RD,PRPF8,IRF9,IFNAR2,STC1,TRIB1,TNKS,NOTCH2,CTSL,GADD45A,RND3,ARHGAP18,NTRK1,DDX58,SLC25A28,RGN,ITGB5,MAOA* |
| Carbohydrate Metabolism | 4.52E-02-1.14E-01 | *PTGFR,TNFAIP6,NTRK1,RGN,GOT1,APOD* |
| Cell Cycle | 4.52E-02-9.96E-02 | *TNKS,NOTCH2,FBXL7,ARHGAP18,GADD45A,NTRK1,IRF9* |
| Cell Morphology | 4.52E-02-1.06E-01 | *PTGFR,TRIB1,ADAM17,GADD45A,OAT,DDX58,NTRK1,SLC25A28,HEY2,TMOD1,MAOA* |
| Cell-To-Cell Signaling and Interaction | 4.52E-02-1.14E-01 | *DDX58,NTRK1,HEY2,ADIPOR2,ITGB5,MAOA* |
| Cellular Assembly and Organization | 4.52E-02-1.15E-01 | *STC1,TNKS,RND3,ARHGAP18,GADD45A,CASQ1,NTRK1,SLC25A28,TMOD1,ITGB5,MAOA* |
| Cellular Function and Maintenance | 4.52E-02-1.15E-01 | *NOTCH2,RND3,ARHGAP18,ERAP1,NTRK1,CASQ1,SLC25A28,HEY2,IFNAR2,TMOD1,ITGB5,MAOA,APOD* |
| Cellular Movement | 4.52E-02-1.14E-01 | *PAPPA,ADAM17,TNFAIP6,TRIB1,NOTCH2,CTSL,RND3,GADD45A,DDX58,NTRK1,RGN,BTBD7,HEY2,ITGB5,MAOA* |
| Drug Metabolism | 4.52E-02-9.65E-02 | *TNFAIP6,MAOA* |
| Energy Production | 4.52E-02-6.73E-02 | *STC1,MAOA* |
| Lipid Metabolism | 4.52E-02-1.11E-01 | *PTGFR,TRIB1,BCAT2,MX1,NTRK1,RGN,GOT1,ADIPOR2,APOD* |
| Molecular Transport | 4.52E-02-1.03E-01 | *STC1,PTGFR,TRIB1,BCAT2,NTRK1,RGN,ADIPOR2,MAOA,APOD* |
| Post-Translational Modification | 4.52E-02-1.17E-01 | *ADAM17,CTSL,ERAP1,OAT,MAOA* |
| RNA Post-Transcriptional Modification | 4.52E-02-4.52E-02 | *DDX58* |
| Small Molecule Biochemistry | 4.52E-02-1.14E-01 | *PTGFR,TNFAIP6,MX1,OAT,STC1,TRIB1,BCAT2,GADD45A,NTRK1,RGN,GOT1,ADIPOR2,APOD,MAOA* |
| DNA Replication, Recombination, and Repair | 5.49E-02-1.14E-01 | *NOTCH2,TNKS,GADD45A,NTRK1* |
| Cellular Compromise | 6.48E-02-1.01E-01 | *NOTCH2,CTSL,PRSS23,NTRK1,TMOD1* |
| Vitamin and Mineral Metabolism | 6.73E-02-6.73E-02 | *RGN* |
| Antigen Presentation | 7.9E-02-9.96E-02 | *CTSL,ERAP1* |
| Protein Synthesis | 7.9E-02-1.17E-01 | *ADAM17,CTSL,ERAP1* |
| Nucleic Acid Metabolism | 8.21E-02-8.21E-02 | *GADD45A* |
| Free Radical Scavenging | 8.85E-02-1.01E-01 | *STC1,PTGFR,PRSS23,RND3,MAOA* |
| Protein Degradation | 1.11E-01-1.17E-01 | *ADAM17,CTSL,ERAP1* |

# Table S. IPA overrepresented molecular and cellular processes for Kinetic

| **Category** | **B-H p-value** | **Molecules** |
| --- | --- | --- |
| Cellular Development | 2.62E-02-1.02E-01 | *OSTM1,STC1,PTGFR,GPRC5B,USP18,TNKS,NOTCH2,TNFAIP6,DDX58,RGN,PRDM1,BTBD7,HEY2,ITGB5,PARP14* |
| Cellular Function and Maintenance | 2.62E-02-8.96E-02 | *USP18,OAS1,FBXL7,SH3BP2,STC1,OSTM1,GPRC5B,NOTCH2,CTSL,SLC25A28,RGN,PRDM1,HEY2,TMOD1,ITGB5* |
| Cellular Growth and Proliferation | 2.62E-02-1.02E-01 | *STC1,PTGFR,USP18,NOTCH2,TNKS,FBXL7,CTSL,TNFAIP6,MX1,DDX58,RGN,SH3BP2,PRDM1,HEY2* |
| Gene Expression | 2.62E-02-9.82E-02 | *PTGFR,OAS1,USP18,NOTCH2,DDX58,PRDM1,HEY2* |
| Carbohydrate Metabolism | 2.62E-02-1.02E-01 | *OSTM1,TNFAIP6,RGN* |
| Cell Death and Survival | 2.62E-02-9.73E-02 | *PTGFR,USP18,OAS1,MX1,SH3BP2,REV3L,OSTM1,STC1,TNKS,NOTCH2,CTSL,DDX58,SLC25A28,RGN,PRDM1,ITGB5,PARP14* |
| Cell Morphology | 2.62E-02-9.82E-02 | *STC1,OSTM1,DDX58,SLC25A28,RGN,PRDM1,REV3L,HEY2,TMOD1* |
| Cell-To-Cell Signaling and Interaction | 2.62E-02-1.04E-01 | *STC1,OSTM1,NOTCH2,CTSL,DDX58,SH3BP2,PRDM1,HEY2,TMOD1,ITGB5* |
| Cellular Assembly and Organization | 2.62E-02-9.73E-02 | *STC1,OSTM1,TNKS,FBXL7,CHCHD10,SLC25A28,CHMP7,TMOD1,ITGB5,TMOD2* |
| Drug Metabolism | 2.62E-02-7E-02 | *TNFAIP6* |
| Energy Production | 2.62E-02-2.62E-02 | *STC1* |
| Lipid Metabolism | 2.62E-02-9.15E-02 | *MX1,RGN* |
| Molecular Transport | 2.62E-02-1.02E-01 | *OSTM1,STC1,RGN* |
| RNA Post-Transcriptional Modification | 2.62E-02-2.62E-02 | *DDX58* |
| Small Molecule Biochemistry | 2.62E-02-9.82E-02 | *STC1,OAS1,CHCHD10,TNFAIP6,MX1,RGN* |
| Cell Signaling | 3.64E-02-8.96E-02 | *STC1,OAS1,MX1,RGN* |
| Cellular Compromise | 3.64E-02-1E-01 | *OSTM1,NOTCH2,FBXL7,CTSL,PRSS23,SH3BP2,REV3L,TMOD1* |
| DNA Replication, Recombination, and Repair | 3.64E-02-7.94E-02 | *TNKS,REV3L* |
| Post-Translational Modification | 3.64E-02-9.82E-02 | *TNKS,USP18,CTSL* |
| Protein Trafficking | 3.64E-02-3.64E-02 | *OSTM1* |
| Cell Cycle | 4.17E-02-9.53E-02 | *TNKS,NOTCH2,SH3BP2* |
| Cellular Movement | 4.17E-02-9.91E-02 | *STC1,NOTCH2,CTSL,TNFAIP6,DDX58,RGN,BTBD7,PRDM1,HEY2,ITGB5* |
| Protein Folding | 4.17E-02-4.17E-02 | *USP18* |
| Vitamin and Mineral Metabolism | 4.66E-02-8.96E-02 | *STC1,RGN* |
| Protein Synthesis | 5.56E-02-9.82E-02 | *CTSL,REV3L* |
| Protein Degradation | 7.66E-02-9.82E-02 | *CTSL* |
| Free Radical Scavenging | 8.4E-02-8.4E-02 | *PTGFR* |
| Nucleic Acid Metabolism | 9.82E-02-9.82E-02 | *OAS1,CHCHD10* |

# Table T. IPA overrepresented molecular and cellular processes for Morphology

| **Category** | **B-H p-value** | **Molecules** |
| --- | --- | --- |
| Cellular Development | 6.2E-03-7.33E-02 | *LAMB2,STC1,TNFAIP6,RGN,HEY2,PARP14* |
| Cellular Growth and Proliferation | 6.2E-03-7.33E-02 | *LAMB2,STC1,TNFAIP6,MX1,RGN,HEY2* |
| Cell Signaling | 6.2E-03-6.7E-02 | *STC1,OAS1,MX1,RGN* |
| Carbohydrate Metabolism | 6.2E-03-7.28E-02 | *OAS1,TNFAIP6,RGN* |
| Cell Death and Survival | 6.2E-03-4.76E-02 | *LAMB2,STC1,OAS1,MX1,RGN,PARP14* |
| Cell Morphology | 6.2E-03-7.28E-02 | *STC1,LAMB2,RGN,HEY2* |
| Cell-To-Cell Signaling and Interaction | 6.2E-03-7.12E-02 | *STC1,LAMB2,RGN,HEY2* |
| Cellular Assembly and Organization | 6.2E-03-4.72E-02 | *STC1,LAMB2* |
| Drug Metabolism | 6.2E-03-7.27E-02 | *STC1,TNFAIP6* |
| Energy Production | 6.2E-03-4.37E-02 | *STC1* |
| Lipid Metabolism | 6.2E-03-7.27E-02 | *STC1,MX1,RGN* |
| Molecular Transport | 6.2E-03-6.7E-02 | *STC1,MX1,RGN* |
| Small Molecule Biochemistry | 6.2E-03-7.28E-02 | *STC1,OAS1,TNFAIP6,MX1,RGN* |
| Vitamin and Mineral Metabolism | 1.48E-02-7.27E-02 | *STC1,MX1,RGN* |
| Cellular Function and Maintenance | 1.63E-02-4.83E-02 | *LAMB2,STC1,RGN,HEY2* |
| Cellular Movement | 1.63E-02-4.44E-02 | *STC1,TNFAIP6,RGN,HEY2* |
| Gene Expression | 3.34E-02-6.7E-02 | *OAS1,HEY2* |
| Free Radical Scavenging | 3.73E-02-5.97E-02 | *STC1* |
| Nucleic Acid Metabolism | 4.37E-02-4.37E-02 | *STC1* |
| Amino Acid Metabolism | 5.73E-02-5.73E-02 | *RGN* |
