# Supplementary material for: Identification of potential biomarkers in donor cows for in vitro embryo production by granulosa cell transcriptomics
Source: PLoS One. 2017 Apr 12;12(4):e0175464. doi: 10.1371/journal.pone.0175464 (PMC5389837; doi:10.1371/journal.pone.0175464)
Supplement: S2 File — (DOCX) [file pone.0175464.s002.docx]

# S2 File

# Identification of potential biomarkers in donor cows for *in vitro* embryo production by granulosa cell transcriptomics

**G. Mazzoni, S.M. Salleh, K. Freude, H.S. Pedersen, L. Stroebech, H. Callesen, P. Hyttel and H.N. Kadarmideen^*^**

* Corresponding author: hajak@dtu.dk

This PDF includes:

**Supplementary Figures: A-D**

# Fig. A. Histogram of the statistics from the post-mapping quality control.

| 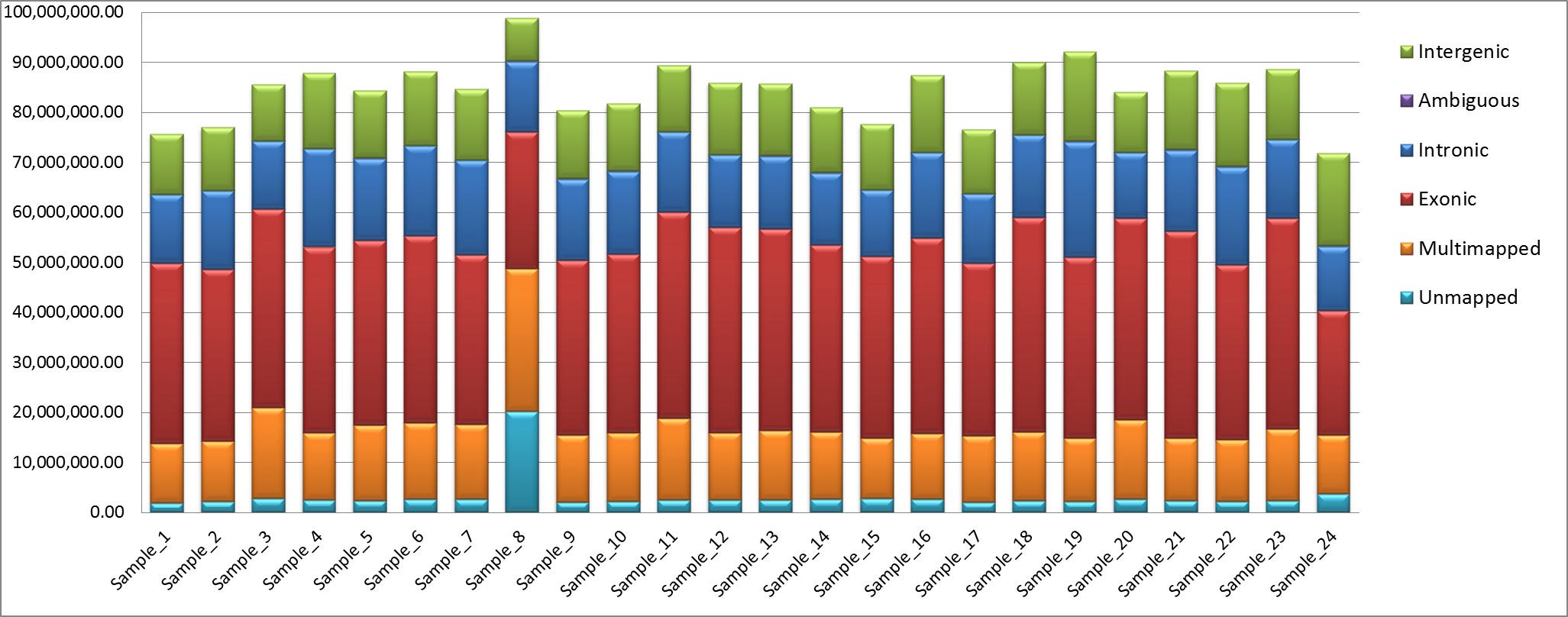 |
| --- |

Each column represents the total number of reads generated for each sample. The fractions of reads are represented in different colours: unmapped, multi-mapped, belonging to read pairs uniquely mapped to exonic, intergenic, intronic regions of the *Bos taurus* reference genome. Read pairs are classified as ambiguous when they map to both intronic and intergenic regions (statistics computed with *Qualimap v.2.0.2*).

# Fig. B. Preliminary analysis of the RNA samples (PCA).

| 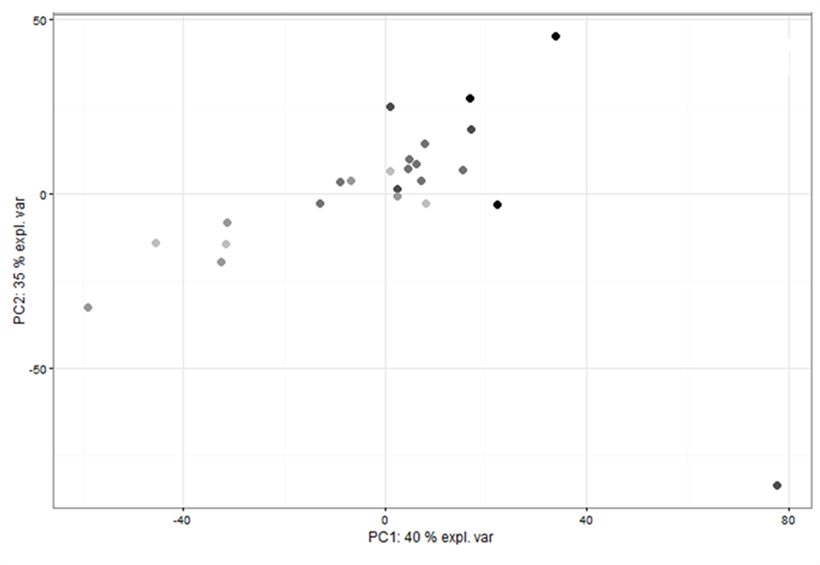 |
| --- |

PCA of the gene expression profiles after filtering low-count genes. Samples are coloured for RIN values. RIN values were divided into six classes and coloured using a scale of colours from black to grey to represent higher and lower RIN values, respectively.

# Fig. C. Preliminary analysis of the RNA samples (hierarchical clustering).

| 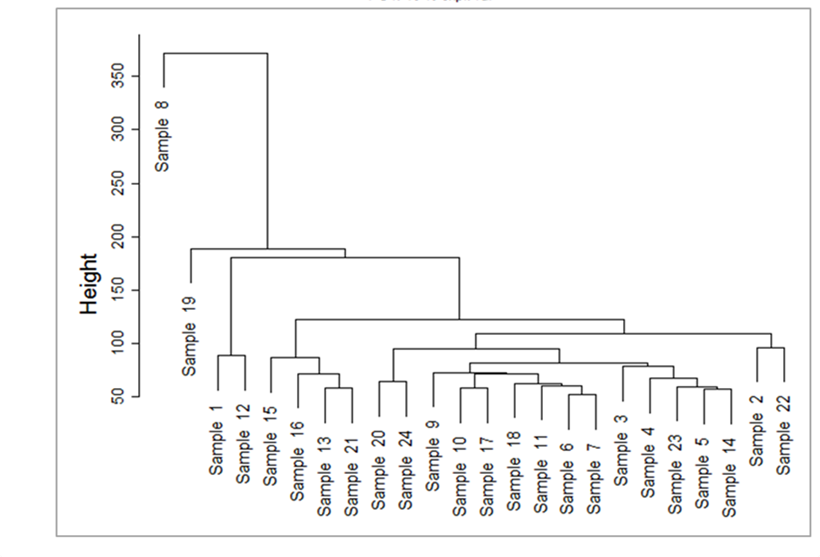 |
| --- |

Hierarchical clustering of the samples based on the entire expression profile after filtering low-count genes. Sample 8 clusters distantly from the other samples.

# Fig. D. Scatter plots for the genes identified as significantly associated in common for all the IVP parameters

| 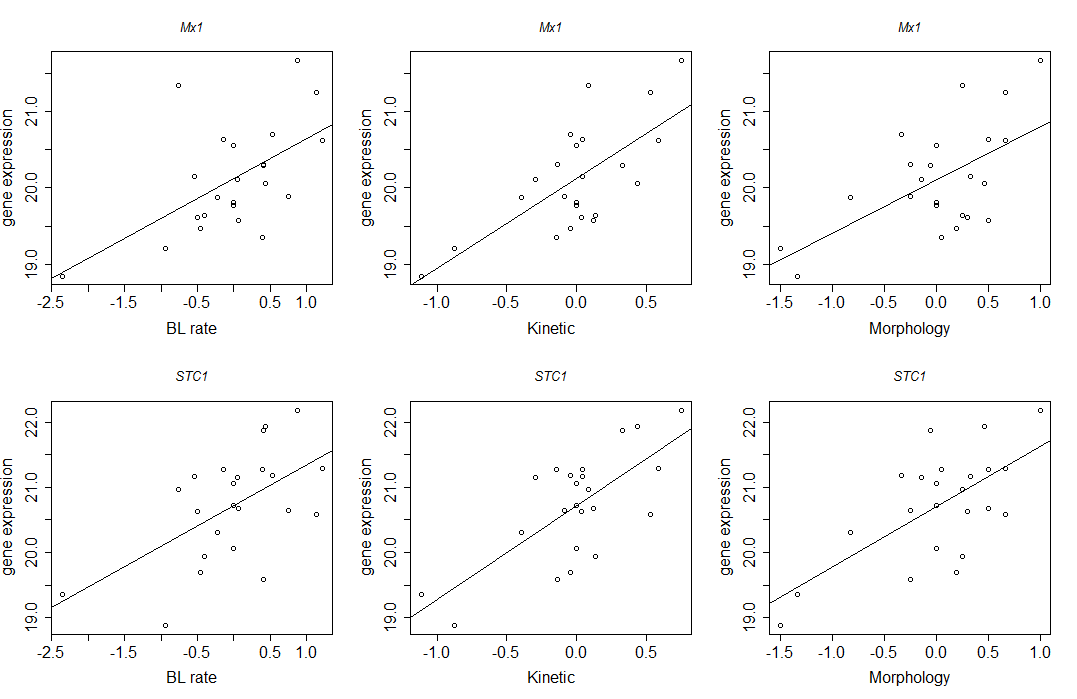 |
| --- |
| 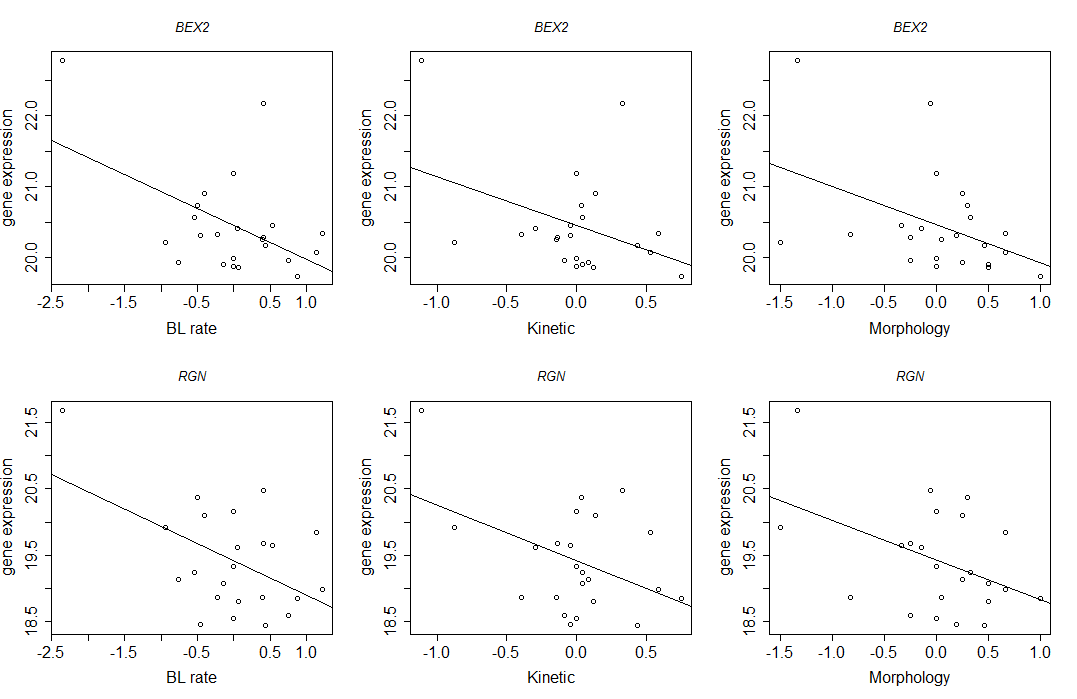 |
| 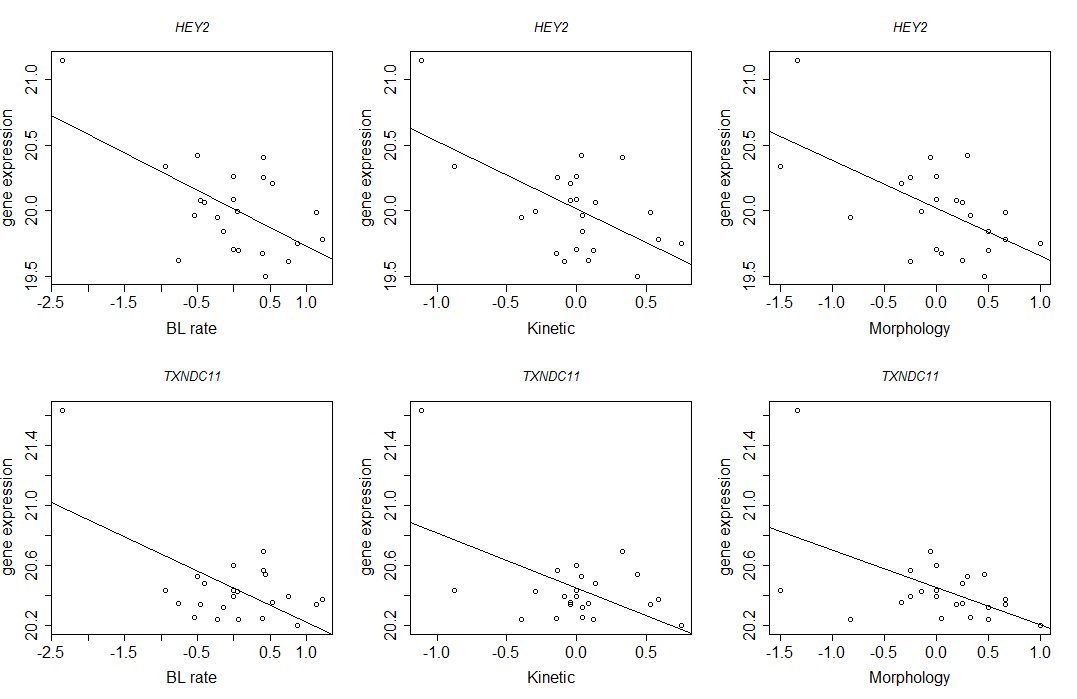 |
| 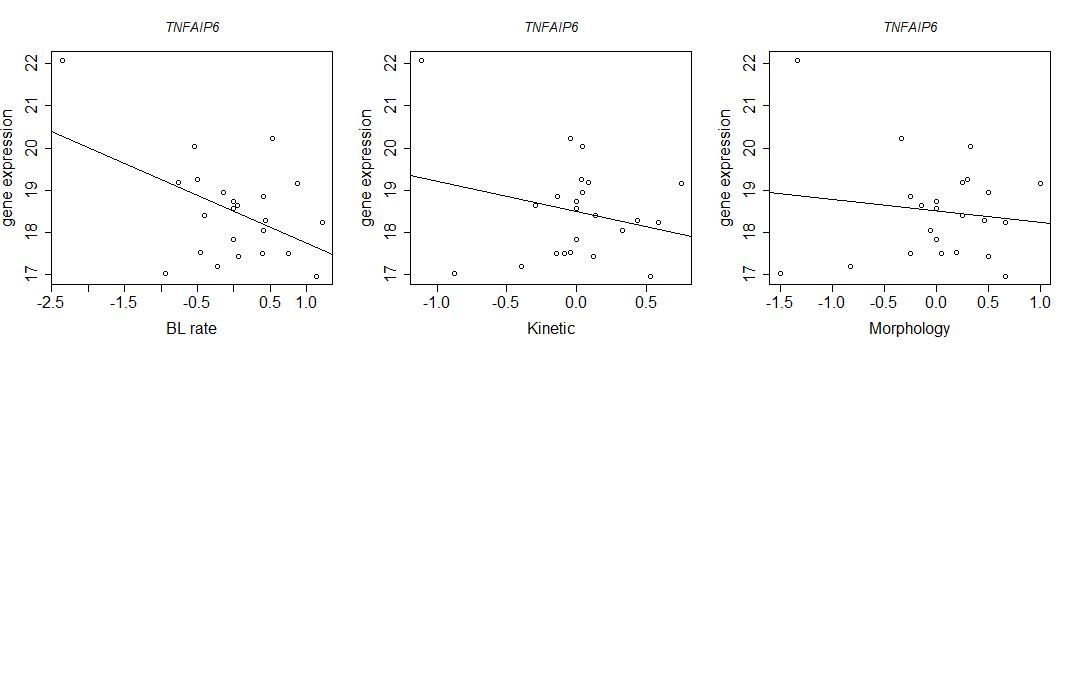 |

The figures represent the normalized gene expression log transformed (y axes) plotted against the three IVP parameters cleaned from the bull effect (x axes).

The line represents the linear regression between genes expression and trait.
