# Supplementary material for: Identification of potential biomarkers in donor cows for in vitro embryo production by granulosa cell transcriptomics
Source: PLoS One. 2017 Apr 12;12(4):e0175464. doi: 10.1371/journal.pone.0175464 (PMC5389837; doi:10.1371/journal.pone.0175464)
Supplement: S1 Text — (DOCX) [file pone.0175464.s003.docx]

# S1 Text

# Identification of potential biomarkers in donor cows for *in vitro* embryo production by granulosa cell transcriptomics

**G. Mazzoni^1^, S.M. Salleh^1^, K. Freude^2^, H.S. Pedersen^4^, L. Stroebech^2,3^, H. Callesen^4^, P. Hyttel^2^ and H.N. Kadarmideen^1*^**

* Corresponding author: hajak@sund.ku.dk

This PDF includes:

**Supplementary text for:**

**-IVP procedures**

**-Follicular cell purification and deep freezing procedure**

## IVP procedure

All media used for the IVP procedure were from IVF Bioscience, Falmouth, United Kingdom, petri dishes and four-well plates were from Nunc/Thermo Fisher Scientific, Roskilde, Denmark and centrifuge tubes from Almeco, Esbjerg, Denmark, unless otherwise stated.

### Cumulus Oocyte Complex (COC) and granulosa cell recovery

The ovaries were transported within 3 hours of retrieval in 0.9% physiological saline solution at 29–32 °C in a thermo container.

Immature COCs were retrieved from antral follicles (2–20 mm in diameter) using a vacuum pump (KNF-Neuberger N86 KN.18, VWR International, Copenhagen, Denmark) connected to an 18-gauge needle (18-G × 1.5, 1.2 × 40, VWR) and a 50 ml tube containing 140 μl heparin (5000 i.u./ml, Leo Pharma, Ballerup, Denmark). The aspirated fluid was transferred into a 10 cm squared gridded petri dishes with 6–8 ml BO-Wash medium (IVF Bioscience, UK). The COCs from each animal were collected and kept separately.

Oocytes without cumulus cells were removed and the aspirated fluids containing only follicular cells were collected in 15 ml TPP centrifuge tubes, one for each animal

#### IVM (in vitro maturation)

COCs were washed three times in BO-Wash medium and once in BO-IVM maturation medium. The COCs were transferred to 0.5 ml BO-IVM medium in four-well plates, pre-equilibrated in 6 % CO_2_ for at least 2 h, keeping all the COCs retrieved from one cow in the same well, and matured at 38.8 °C in 5.5–6 % CO_2_ with humidified atmospheric air (21% O_2_) for 20–24 hours.

#### IVF (in vitro fertilization)

All experiments were carried out using frozen–thawed semen from 19 Holstein chosen bulls with extreme NTM indexes – nine bulls with low (NTM) index (NTM range: 1–7) and 10 bulls with high NTM index (NTM range: 31–35) (Viking Genetics, Randers, Denmark).

Matured COCs were rinsed carefully once in IVF medium and then transferred to a four-well plate with 0.4 ml BO-IVF in each well (pre-equilibrated in a 6 % CO_2_ incubator for at least 2 h). Straws containing frozen semen from selected bulls were thawed at 37 °C. Sperm motility was evaluated for each straw with an optical microscope. Each semen dose was washed twice by centrifugation at 328 g for 5 min in BO-SemenPrep (warmed up to 37 °C) in 15 ml TPP centrifuge tubes (ALMECO), and finally resuspended in a total volume of 700 μl. All the samples were inseminated with 40 μl of the final semen solution, assuming the final sperm concentration to be a minimum of 2x10^6^/ml in the IVF wells, corresponding to the concentration in routine experiments in the University of Copenhagen IVF laboratory.

Insemination was performed according to the experimental design: each bull was used with three pools of oocytes, and for each experiment, half of the pools were inseminated with bulls with high NTM and half with low-NTM bulls. The spermatozoa were co-incubated with matured COCs in BO-IVF medium for 22–24 h without oil overlay at 38.8 ° C in 6% CO_2_ in ambient humidified air (21 % O_2_) overnight.

#### IVC (in vitro culture)

Presumed zygotes were transferred to BO-Wash medium and denuded of surrounding cumulus cells by using a denuding micropipette EZ-Squeeze 135 μm (Research Instruments, UK). Zygote numbers higher than 25 were vortexed for 2 minutes using polystyrene centrifuge tubes (Nunc™) in order not to delay handling thereby introducing potential bias. The denuded presumptive zygotes were washed in BO-Wash, rinsed in IVC culture medium and cultured in 500 μl BO-IVC culture medium with 400 μl BO-Oil overlay in low oxygen atmosphere, 6 % O_2,_ at 38.8 °C and 6 % CO_2_ overnight until BL stage day 8.

## Follicular cell purification and deep freezing procedure

Follicular cells were centrifuged at 300 RCF for 5 minutes followed by a second centrifugation at 2700 RCF for 1 minute and removal of the supernatant. The pellet was incubated with 200 µl Accutase (Gibco StemPro, Life Technologies) at 37 °C for 5 minutes in order to dissociate cells into single cells. The enzyme activity was interrupted by adding 200 µl BO-Wash medium. The samples were centrifuged again at 2700 RCF for 1 minute, and the supernatant removed and washed with 500 µl phosphate-buffered saline (PBS). The pellet was collected after centrifugation (at 2700 RCF for 1 minute).

We purified the cells to remove possible contamination from blood that may have occurred during the aspiration step and in particular to avoid contamination from leukocytes whose expression is found to be similar to granulosa cells in other species ^18^. The purification was done in two steps.

In the first step, we reduced the concentration of the leukocytes using a filtration method based on beads labelled with anti-bovine CD45 antibodies (pluriSelect S-pluriBead***^®^*** GmbH, Leipzig, Germany) following the manufacturer’s instructions. Briefly, the cells were incubated for 30 minutes in a rotator together with pluriSelect incubation buffers and 60 µl of the pluriBeads mixture. The samples were then filtered through the pluriSelect strainers, which enabled collection of the beads with bound leukocytes. The strainers are washed twice with buffer and the flow-through collected.

In the second step, we used erythrocytes lysis buffer to reduce the presence of erythrocytes ^36^. The pellets were collected and incubated with 150 µl of erythrocytes lysis buffer (Qiagen, Hilden, Germany) for 10 minutes in a cold bath at 4 °C. The erythrocytes lysis buffer was removed after centrifugation at 2700 RCF for 1 minute. The pellet was washed once with PBS, snap-frozen in liquid nitrogen and stored at -80 °C until RNA extraction.
